# The two‐component system CroRS acts as a master regulator of cell envelope homeostasis to confer antimicrobial tolerance in the bacterial pathogen *Enterococcus faecalis*

**DOI:** 10.1111/mmi.15128

**Published:** 2023-07-20

**Authors:** Francesca O. Todd Rose, Rachel L. Darnell, Sali M. Morris, Olivia E. Rose, Olivia Paxie, Georgia Campbell, Gregory M. Cook, Susanne Gebhard

**Affiliations:** ^1^ Department of Microbiology and Immunology University of Otago Dunedin New Zealand; ^2^ Maurice Wilkins Centre for Molecular Biodiscovery University of Otago Dunedin New Zealand; ^3^ Department of Life Sciences, Milner Centre for Evolution University of Bath Bath UK; ^4^ Institut für Molekulare Physiologie, Mikrobiologie und Biotechnologie, Johannes Gutenberg‐Universität Mainz Germany

**Keywords:** bacterial, cell wall, drug resistance, *Enterococcus faecalis*, isoprenoids, regulation, RNA‐seq, tolerance

## Abstract

Antimicrobial tolerance is the ability of a microbial population to survive, but not proliferate, during antimicrobial exposure. Significantly, it has been shown to precede the development of bona fide antimicrobial resistance. We have previously identified the two‐component system CroRS as a critical regulator of tolerance to antimicrobials like teixobactin in the bacterial pathogen *Enterococcus faecalis*. To understand the molecular mechanism of this tolerance, we have carried out RNA‐seq analyses in the *E. faecalis* wild‐type and isogenic ∆ *croRS* mutant to determine the teixobactin‐induced CroRS regulon. We identified a 132 gene CroRS regulon and demonstrate that CroRS upregulates biosynthesis of all major components of the enterococcal cell envelope in response to teixobactin. This suggests a coordinating role of this regulatory system in maintaining integrity of the multiple layers of the enterococcal envelope during antimicrobial stress, likely contributing to bacterial survival. Using experimental evolution, we observed that truncation of HppS, a key enzyme in the synthesis of the quinone electron carrier demethylmenaquinone, was sufficient to rescue tolerance in the *croRS* deletion strain. This highlights a key role for isoprenoid biosynthesis in antimicrobial tolerance in *E. faecalis*. Here, we propose a model of CroRS acting as a master regulator of cell envelope biogenesis and a gate‐keeper between isoprenoid biosynthesis and respiration to ensure tolerance against antimicrobial challenge.

## Graphical abstract

In *Enterococcus faecalis*, CroRS regulates the expression of cell envelope biosynthesis pathways to maintain homeostasis and confer tolerance upon antimicrobial challenge. Loss of function mutations in the respiratory branch of the isoprenoid pathway appears to rescue tolerance in a *croRS* deletion mutant.

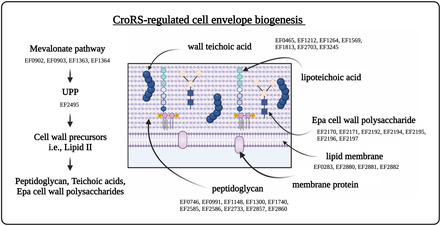

## 1 INTRODUCTION

The emergence of multidrug‐resistant bacterial pathogens has rendered standard treatments ineffective, allowing infections to persist and spread. Significantly, antimicrobial tolerance (AMT), that is, the ability of a bacterium to survive but not proliferate during antimicrobial exposure, has been shown to precede the development of bona fide antimicrobial resistance (Levin‐Reisman et al., 2017; Liu et al., 2020; Santi et al., 2021; Windels et al., 2020). Enterococci are a group of Gram‐positive bacteria that inhabit a wide variety of ecological niches such as food, fresh water and the gastrointestinal tract of humans, animals and insects (Van Tyne & Gilmore, 2014). Although primarily commensals, enterococci are also clinically significant opportunistic pathogens that can exploit a compromised host to cause diseases such as urinary tract infections, bacteraemia and endocarditis (Arias & Murray, 2012). *Enterococcus faecalis* and *Enterococcus faecium* are the most abundant enterococcal species in humans and a leading cause of hospital‐acquired infection (Moellering, 1992).

Two‐component systems constitute an important regulatory network of the cell envelope stress response in *Enterococcus faecalis*, with a number implicated in antimicrobial resistance, that is, cephalosporin resistance, CroRS and daptomycin resistance, LiaFSR (Arias et al., 2011; Comenge et al., 2003; Hancock & Perego, 2004; Kellogg et al., 2017; Muller et al., 2018). In addition, we have previously identified CroRS as a critical regulator of antimicrobial tolerance (Darnell et al., 2019). CroRS is encoded on a bicistronic operon with *croS* encoding the sensor kinase and *croR* its cognate response regulator (Comenge et al., 2003). Taken together, these studies demonstrate the crucial role of CroRS in mediating the response to antimicrobial attack in *E. faecalis*. However, the precise architecture of the response network and the molecular mechanism(s) conferring this tolerance require further elucidation.

Previous transcriptional analyses of the CroRS regulon have used RNA‐seq to identify genes differentially expressed in the presence and absence of antimicrobial stress (Muller et al., 2018; Timmler et al., 2022). Muller et al. (2018) identified 50 potential CroR‐regulated genes in the *E. faecalis* JH2‐2 strain in the absence of antimicrobial stress (Muller et al., 2018), while Timmler et al. (2022) identified 87 CroR‐regulated genes differentially expressed in the *E. faecalis* OG1RF strain in the presence of bacitracin‐induced antimicrobial stress. In the OG1RF strain, CroS has two cognate response regulators CroR and CisR, of which CisR is notably absent in the JH2‐2 strain (Kellogg & Kristich, 2016). In addition, while CroRS is known to respond to the presence of bacitracin, *E. faecalis* displays only low‐level bacitracin tolerance (Darnell et al., 2019; Timmler et al., 2022). Therefore, the tolerance‐inducing CroRS regulon remains undiscovered.

Teixobactin (TXB) represents a new class of antimicrobial with a unique chemical scaffold and lack of detectable resistance (Ling et al., 2015). It has proven efficacy against multidrug‐resistant pathogens such as enterococci, staphylococci and *Mycobacterium tuberculosis* and has been shown to induce cell lysis through binding of cell wall precursors lipid II and lipid III in *Staphylococcus aureus* (Homma et al., 2016; Ling et al., 2015). Further to this mechanism, recent studies suggest that TXB potency is amplified by the formation of TXB‐lipid II clusters (Shukla et al., 2020, 2022). The unique enduracididine C‐terminal headgroup of TXB specifically binds to the conserved pyrophosphate‐saccharide moiety of cell wall precursors such as lipid II and lipid III while the N‐terminus coordinates with a second pyrophosphate from another lipid II molecule (Shukla et al., 2022). Clustering of lipid II within this structure is thought to displace phospholipids and disrupt the membrane, generating a simultaneous action against cell wall synthesis and the cytoplasmic membrane to produce a highly effective antimicrobial (Shukla et al., 2022).

Antimicrobials are thought to kill bacteria through interaction with specific intracellular targets (Kohanski et al., 2010). Antimicrobial drug‐target interactions, and their respective direct effects, are generally well characterised. By contrast, the bacterial responses to antimicrobial treatments that contribute to cell death are not as well understood and have proven to be complex as they involve many genetic and biochemical pathways (Kohanski et al., 2010). It is currently unknown how CroRS protects against killing by cell wall‐targeting antimicrobials such as TXB and the glycopeptide vancomycin. However, by determining the antimicrobial‐induced CroRS regulon, we can gain a better understanding of the physiological processes this regulatory system controls, and begin to uncover its role in AMT. In this study, we determined the TXB‐induced CroRS regulon and identify key genes and pathways involved in mediating CroRS‐regulated AMT. We show CroRS regulates the expression of all major pathways of cell envelope biosynthesis and that AMT can be rescued in a *croRS* deletion strain through the loss of function of a heptaprenyl diphosphate synthase (*hppS*). As a consequence, we propose a revised model suggesting CroRS functions as a gatekeeper of isoprenoid flux between cell wall biosynthesis and respiratory energy metabolism to confer AMT in *E. faecalis*.

## 2 RESULTS AND DISCUSSION

### 2.1 Whole‐genome transcription profiling of the *E. faecalis* WT and ∆ *croRS* mutant in the presence and absence of teixobactin

To understand how CroRS contributes to TXB tolerance, we first aimed to identify which genes it controls in response to TXB exposure. Previous optimisation with the *E. faecalis* JH2‐2 wild‐type (WT) showed challenge with 0.5 μg/mL of TXB for 1 h on mid‐exponential phase cells was optimal for inducing a CroRS response without significantly impacting growth (Darnell et al., 2019). These conditions were also deemed appropriate for the ∆ *croRS* strain, with no difference in growth inhibition or cell viability observed between ∆ *croRS* and the WT under these conditions (Figure S1) (Darnell et al., 2019). To identify the TXB‐induced CroRS regulon, four different gene expression profiles (>1.0 fold‐log_2_) were generated following RNA‐seq: (1) WT untreated versus ∆ *croRS* untreated (Table S1), (2) WT treated versus ∆ *croRS* treated (Table S2), (3) WT treated versus untreated (Table S3) and (4) ∆ *croRS* treated versus untreated (Table S4). These four gene expression profiles were then fed into a filtering system to isolate the TXB‐induced CroRS regulon (Figure S2). This filtering system was deliberately chosen for its stringence to allow us to specifically identify genes controlled by CroRS in response to TXB and their role in TXB tolerance. However, it is important to note that here we treat the ‘CroRS regulon’ as an umbrella term for all genes under CroRS regulation, whether this be through direct binding or as a result of downstream regulation by genes CroRS controls, to include all genes that may contribute to CroRS‐mediated AMT.

### 2.2 The teixobactin‐induced CroRS regulon

A total of 538 genes were differentially expressed (>1.0 fold‐log_2_) in the WT versus ∆ *croRS* in the presence of TXB (Table S1). However, only 132 genes were considered to belong to the TXB‐induced CroRS regulon (Table S5). Of these 132 genes, 117 were upregulated and 15 were downregulated. To validate the RNA‐seq data, we performed qRT‐PCR to analyse the expression of a subset of 11 genes, five of which were identified in the CroRS regulon. For these experiments, the *E. faecalis* WT and ∆ *croRS* strains were grown to mid‐exponential phase and challenged in the presence and absence of TXB. Differential gene expression was consistent and comparable to the reported RNA‐seq data (Figure S3; Table S6). The regulatory networks in enterococci are not completely understood, and therefore, we could not map the 132 CroRS regulon genes against known regulons of other regulatory systems. Instead, we sought to identify metabolic pathways up‐ and downregulated by CroRS in response to TXB challenge. Genes were therefore categorised into KEGG gene ontologies using the well‐defined *E. faecalis* V583 strain as a reference to maintain continuity with published data.

**FIGURE 1:**
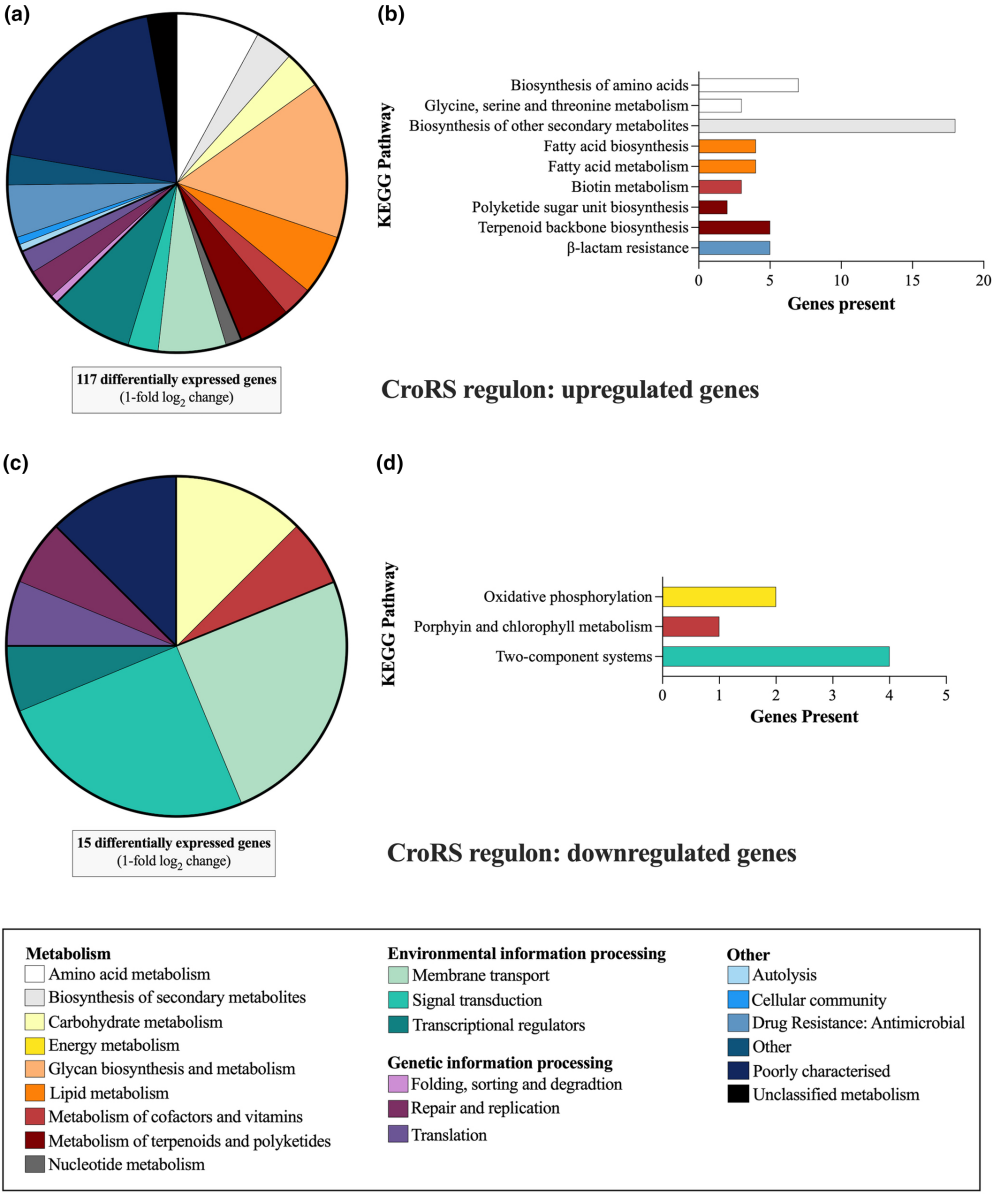
Functional classification and distribution of TXB‐induced CroRS‐regulated genes. Genes differentially expressed (>1‐fold log_2_) in the TXB‐induced CroRS regulon were assigned to the well‐defined *Enterococcus faecalis* V583 KEGG ontologies. These pie charts represent the distribution of these ontologies up and downregulated by the TXB‐induced CroRS regulon (a, c). In addition, hypergeometric testing was performed on the TXB‐induced CroRS regulon to identify ontologies significantly (*p* =< 0.05) up and downregulated (b, d).

**FIGURE 2:**
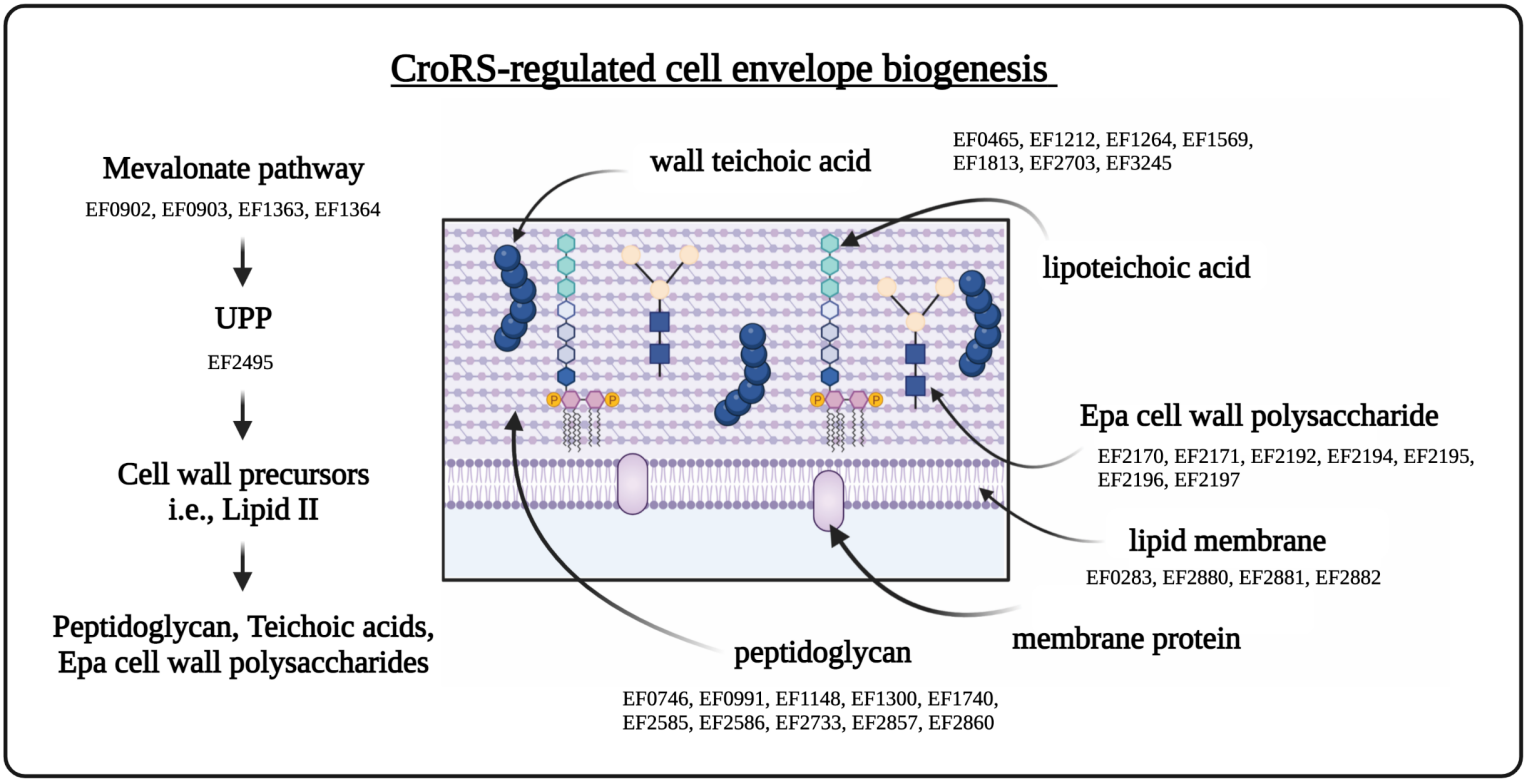
CroRS‐regulated pathways of the enterococcal cell envelope in response to TXB stress. The enterococcal cell envelope is composed of two main layers: a cytoplasmic lipid membrane, surrounded by a thick cell wall. The mevalonate pathway is essential for synthesis of UPP. UPP serves as a key membrane anchor and carrier of cell wall precursors required for peptidoglycan, wall teichoic acid and Epa biosynthesis. Peptidoglycan is the major component of the cell wall, with teichoic acids (wall and lipoteichoic) and the Epa (enterococcal polysaccharide antigen) cell wall polysaccharides as the two other major constituents. CroRS‐regulated expression of genes required for the biosynthesis of each of these components in response to teixobactin challenge is listed below each respective pathway. In the case of wall and lipoteichoic acid synthesis, CroRS‐regulated genes are located between these two pathways due to their shared role in these pathways.

Over 25% of genes upregulated by CroRS were involved in cell envelope biogenesis (Figure 1a). Strikingly, as detailed below, this included all layers of the enterococcal cell envelope, that is, the cytoplasmic membrane (lipid metabolism), the peptidoglycan cell wall (metabolism of terpenoids and polyketides and drug resistance), teichoic acids and cell wall polysaccharides (glycan biosynthesis and metabolism) (Figures 1a and 2). Other pathways highly upregulated included amino acid biosynthesis and membrane transport (Figure 1a). In comparison, carbon metabolism, membrane transport and signal transduction were downregulated by CroRS in response to TXB (Figure 1c). In addition, each ontology was analysed for significance, using hypergeometric testing to compare the number of genes with changed expression to the total number of genes within a ontology. This confirmed significant upregulation of cell envelope biogenesis, amino acid biosynthesis and metabolism pathways (Figure 1b), as well as downregulation of haem transport, oxidative phosphorylation and signal transduction pathways (Figure 1d).

### 2.3 CroRS regulates the expression of cell envelope biosynthesis pathways in response to TXB challenge

We have previously shown the induction of cell envelope stress by TXB in *E. faecalis* WT (Darnell et al., 2019). Here, we show that CroRS controls this response by upregulating genes involved in the biogenesis of all major components of the enterococcal cell envelope in response to TXB challenge (Table 1; Figure 2). A number of these genes have been shown previously to be upregulated by CroR in the *E. faecalis* strain OG1RF in response to antimicrobial challenge (Timmler et al., 2022). This includes the putative peptidoglycan‐modifying enzymes *murT* and *gatD*, *mvaS* of the mevalonate pathway, penicillin‐binding proteins *pbpA* and *pbp(6)*, the lipoteichoic acid synthases EF1264 and EF1813 and the putative *tagU* homologue EF3245 (Table 1) (Timmler et al., 2022). Interestingly, only one gene, *pbp(6)*, was homogenously identified between our RNA‐seq data and the previously reported *E. faecalis* JH2‐2 CroR regulon determined in the absence of antimicrobials (Muller et al., 2018). However, we also observe CroRS‐regulated expression of genes involved in lipid II biosynthesis, for example, *murAA*, *murAB*, *murC*, *murE* and *pbp4(5)*, through differential expression between the WT and ∆ *croRS* strain in the absence of TXB (Table S1). As a consequence, we hypothesise CroRS regulates different sets of genes to maintain cell envelope homeostasis under stressed (as presented here and by Timmler et al), and non‐stressed conditions (as presented by Muller et al) (Muller et al., 2018; Timmler et al., 2022).

**TABLE 1:** Differential gene expression in the *Enterococcus faecalis* WT versus ∆ *croRS* strain in the presence of TXB.

Pathway	Gene^a^	Name	F/C^b^	Function
Mevalonate pathway	EF0902	*mvaK*	2.9	Phosphomevalonate kinase
	EF0903	*mvaD*	3.7	Diphosphomevalonate decarboxylase
	EF1363	*mvaS*	4.4	HMG‐CoA synthase
	EF1364	*mvaE*	4.6	acetyl‐CoA acetyltransferase/HMG‐CoA reductase
UPP biosynthesis	EF2495	*uppS*	3.3	UDP‐diphosphate synthase
	EF0746	*pbp(6)*	5.4	Penicillin‐binding protein
Peptidoglycan biosynthesis and modification	EF0991	*pbpC*	2.8	Penicillin‐binding protein C
	EF1148	*pbp1A/ponA*	2.6	Penicillin‐binding protein 1A
	EF1300		2.9	Putative lipid II flippase
	EF1740	*pbp1B/pbpZ*	2.2	Penicillin‐binding protein 1B
	EF2585	*murT*	3.3	mur ligase
	EF2586	*gatD*	3.0	Glutamine amidotransferase
	EF2733	*murB*	2.7	UDP‐*N*‐acetylenolpyruvoylglucosamine reductase
	EF2857	*pbp2B/pbpA*	4.8	Penicillin‐binding protein 2B
	EF2860	*pbp*	2.5	Putative peptidoglycan transpeptidase
Epa polysaccharide biosynthesis	EF2170^c^	*epaX*	2.1	Glycosyl transferase group 2 family protein
	EF2171^c^	*epaW*	2.8	dTDP‐4‐dehydro‐6‐deoxy‐D‐glucose
	EF2192	*epaG*	2.2	dTDP‐glucose 4,6‐dehydratase
	EF2193	*epaF*	2.7	dTDP‐4‐dehydrorhamnose 3,5‐epimerase
	EF2194	*epaE*	2.6	Glucose‐1‐phosphate thymidylyltransferase
	EF2195	*epaD*	1.9	Glycosyl transferase group 2 family protein
	EF2196	*epaC*	1.9	Glycosyl transferase group 2 family protein
	EF2197	*epaB*	1.9	Glycosyl transferase group 2 family protein
Teichoic acids biosynthesis	EF0465		2.0	Polyisoprenyl‐teichoic acid‐‐peptidoglycan teichoic acid transferase
	EF1212		4.5	Lcp ligase
	EF1264		1.5	Lipoteichoic acid synthase
	EF1569		3.5	Lcp ligase
	EF1813		3.4	Lipoteichoic acid synthase
	EF2703		5.4	Lcp ligase
	EF3245		5.2	Cell‐envelope‐associated acid phosphatase
Fatty acid synthesis	EF0283	*fabF‐1/fabO*	1.6	3‐oxoacyl‐ACP synthase
	EF2880	*fabF‐2*	1.5	3‐oxoacyl‐ACP synthase
	EF2881	*fabGa*	1.8	3‐ketoacyl‐ACP reductase
	EF2882	*fabD*	1.4	ACP *S*‐malonyltransferase

^a^ *Enterococcus faecalis* V583 gene number is stated to maintain consistency with the literature.

^b^ Fold‐change log_2_.

^c^ Genes are also involved in wall teichoic acid biosynthesis.

Enterococci utilise the mevalonate (MVA) pathway to synthesise the isoprenoid precursors farnesyl pyrophosphate (FPP) and isopentenyl pyrophosphate (IPP) for generation of the essential cell wall lipid carrier undecaprenyl pyrophosphate (UPP), as well as quinones such as demethylmenaquinone (DMK) for electron transport. In enterococci, the MVA pathway consists of five genes, four of which were upregulated (2.9–4.6 fold‐log_2_) by CroRS in response to TXB (Table 1). The fifth, *mvk* (EF0904), looks to be in a conserved operon with *mvaD* and *mvaK* (Heuston et al., 2012), and fulfils all criteria for the TXB‐dependent CroRS regulon except that it was differentially expressed (−2.1 fold‐log_2_) in the WT versus ∆ *croRS* strain in the absence of TXB (Table S1). Interestingly, mutations in the MVA pathway in *E. faecalis*, *S. aureus* and *S. pneumoniae* have been shown to play a critical role in peptidoglycan biosynthesis, with suppressor mutations in *S. pneumoniae* decreasing peptidoglycan precursors and resensitising the bacterium to amoxicillin (Dewachter et al., 2022; Matsumoto et al., 2016).

Following synthesis and translocation of the cell wall biosynthesis intermediate lipid II across the membrane, the new peptidoglycan building block is integrated into the peptidoglycan matrix by penicillin‐binding proteins. We observed an upregulated expression of six (from a total of eight) penicillin‐binding proteins (PBP) by CroRS in response to TXB (Table 1). This included the transglycosylase genes *pbp1A* and *pbp1B*, the transpeptidase *pbp2B*, the unclassified *pbpC* and *pbp(6)* and the putative peptidoglycan transpeptidase EF2860 (Table 1). Targeted deletion of neither *pbp(6)* nor EF2860 conferred any change in tolerance to TXB or vancomycin (data not shown). However, *pbpC*, *pbp2B* and *pbp1A* have been shown to have an important role in enterococcal growth and cephalosporin resistance respectively (Arbeloa et al., 2004; Djorić et al., 2020; Gilmore et al., 2020; Moon et al., 2018). Interestingly, *pbp4(5)* (EF2476) which was previously isolated as a component of the CroRS‐regulon was not identified here (Timmler et al., 2022). This was because, while we observed a 2.0‐fold upregulation of *pbp4(5)* in the WT response to TXB, we also observed a 4.2‐fold upregulation in the WT compared to ∆ *croRS* in the presence of TXB, we also observed a 1.7‐fold increase in expression of *pbp4(5)* in the WT compared to ∆ *croRS* in the absence of TXB, disqualifying this gene from our assignment to the TXB‐induced CroRS regulon. While this suggests some CroRS‐regulated genes have been filtered out using our system, it supports our aim to isolate genes specifically induced under TXB‐challenge in a CroRS‐dependent manner to elucidate their role in AMT.

The enterococcal polysaccharide antigen (Epa) is another major component of the enterococcal cell envelope. Epa biosynthesis is encoded by two gene clusters, one conserved and one variable (Dale et al., 2015; Guerardel et al., 2020; Palmer et al., 2012; Rigottier‐Gois et al., 2015). The conserved gene cluster (EF2198‐EF2177; *epaA–epaR*) is responsible for the biosynthesis of the rhamnopolysaccharide backbone, and mutations in *epaB* and *epaE* have been shown to increase susceptibility to salt and cell envelope stress (Smith et al., 2019; Solheim et al., 2014). The variable gene cluster (EF2177‐EF2164) is responsible for the biosynthesis and assembly of the teichoic acids covalently linked to the conserved backbone (Guerardel et al., 2020; Smith et al., 2019). This variable region is responsible for conferring the major differences in Epa between *E. faecalis* isolates, and deletion of genes in this cluster, such as the *epaX*‐like gene (EF2170), has been shown to reduce peptidoglycan crosslinking and resistance to the autolytic compound lysozyme. In addition, mutations that result in a loss of Epa have been associated with altered susceptibility to antimicrobials (Korir et al., 2019; Singh & Murray, 2019; Solheim et al., 2014). Our data now show that CroRS regulates the expression of five genes in the conserved gene cluster (*epaB*, *C*, *D*, *E*, *F* and *G*), as well as the two variable genes, *epaW* and *epaX* (Table 1).

Taken together, our data suggest an involvement of CroRS‐regulation in the synthesis of all layers of the enterococcal cell envelope (Figure 2), with many of its target genes already identified as important for antimicrobial resistance in previous studies. The specific contribution of these genes to AMT remains to be tested experimentally, as most studies so far have exclusively focussed on resistance. However, the central role CroRS appears to play in controlling, and likely coordinating, the synthesis of the entire cell envelope under antimicrobial exposure offers a plausible explanation for the pleiotropic phenotype of the *croRS*‐deletion strain, including its loss of tolerance to cell envelope‐acting antimicrobials.

### 2.4 Experimental evolution of *E. faecalis* ∆ *croRS* for growth recovery

To identify focal pathways within the regulon controlled by CroRS, we next sought to isolate suppressor mutations via experimental evolution. Initially, we attempted to rescue the ∆ *croRS* strain for tolerance in the presence of TXB. However, this failed to produce suppressor mutants able to survive in the presence of TXB. Therefore, to reduce the selection pressure, we chose to exploit the known growth defect of the ∆ *croRS* strain, on the assumption that restoration of WT growth might also correct the physiological defect that confers a loss in AMT (Hancock & Perego, 2004; Le Breton et al., 2003). In brief, *E. faecalis* ∆ *croRS* was serially passaged into fresh liquid medium every 2 days to select for faster growing mutants in the population. High turbidity of an overnight growth culture was taken as indicative of restored WT‐like growth behaviour. After 10–14 days, five clones, one from each independently evolved line, were isolated for further characterisation and named 1BS to 5BS. Growth curves of these mutants demonstrated a decrease in both the lag phase and exponential phase doubling time compared to the ∆ *croRS* parent strain, albeit not completely reaching WT growth characteristics, thus confirming partial restoration of the growth defect (Figure S4).

CroRS is activated in the presence of a number of cell‐wall‐acting antimicrobials (Abranches et al., 2013; Darnell et al., 2019; Timmler et al., 2022). To determine whether the growth‐passaged (BS) mutants were also recovered for AMT, antimicrobial susceptibility assays were carried out for the CroRS‐activating cell wall antimicrobials, vancomycin, TXB and bacitracin (Table 2). Despite evolution in the absence of drugs, all mutants were rescued for TXB and vancomycin tolerance, as determined by MBC (minimum bactericidal concentration) values. Interestingly, the evolved strains showed a decrease in resistance against vancomycin and TXB (2‐fold), as determined by MIC values (Table 2). This suggests that resistance and tolerance are mechanistically different, and the mutation(s) that restore the growth defect only contribute to AMT.

**TABLE 2:** Antimicrobial susceptibility and whole‐genome sequence analysis of the evolved ∆ *croRS* mutants.

Strain	Vancomycin		Teixobactin		Bacitracin		Gene	SNP	Codon
	MIC^a^	MBC	MIC	MBC	MIC	MBC			
WT	1	>128	2	16–32	16	64			
Δ*croRS*	0.25	<4	2	<4	8–16	8–16			
1BS	0.125	128	1	8–16	16	32	Heptaprenyl diphosphate synthase; *hppS* (EF2057)	216_225del	K72
							*N*‐acetylmuramoyl‐L‐amidase; (EF2367)	52C>T	Q18*
2BS	0.125	128	1	16	16	32	Heptaprenyl diphosphate synthase; *hppS* (EF2057)	567T>A	Y189*
3BS	0.125	128	1–2	16	16	32	DNA‐directed RNA polymerase subunit delta; *rpoE* (EF1146)	584_585delTTinsAA	V195E
							Hypothetical protein; (EF0062)	3848A>T	K1283I
4BS	0.125	128	1	32	16	32	Heptaprenyl diphosphate synthase; *hppS* (EF2057)	91G>T	E31*
5BS	0.125	128	1	32	32	32	Phosphate transporter ATP‐binding protein; *pstBB* (EF1756)	661A>G	T221A
							*N*‐acetylmuramoyl‐L‐amidase; (EF2367)	482T>G	V161G
							Hypothetical protein; (EF0062)	3848A>T	K1283I

^a^ MICs and MBCs are given as the median range in μg/mL and are representative of at least biological triplicate.

Whole‐genome sequencing of these mutants revealed key mutations in a heptaprenyl diphosphate synthase, *hppS* (EF2057;1BS, 2BS, 4BS), the delta subunit of the DNA‐directed RNA polymerase, *rpoE* (EF1146; 3BS), an *N*‐acetylmuramoyl‐L‐amidase (EF2367; 1BS, 5BS), a hypothetical protein (EF0062; 3BS, 5BS), and *pstBB*, a phosphate ABC transporter ATP‐binding protein (EF1756; 5BS) (Table 2). HppS is a key enzyme in the biosynthesis of DMK, the quinone redox carrier for electron transport and catalyses the formation of heptaprenyl pyrophosphate (HepPP) from the isoprenoid precursors FPP and IPP (Desai et al., 2016). HepPP is then used as a substrate by the enzyme MenA (1,4‐dihydroxy‐2‐napthoate octaprenyl transferase) to catalyse the formation of DMK. Effectively, HppS is therefore the branching point in isoprenoid synthesis where the precursors are either directed towards synthesis of UPP for cell envelope biogenesis, or towards DMK synthesis for electron transport.

Of the three strains carrying *hppS* mutations, two, namely 2BS and 4BS, carried only these mutations (Figure S5a). In addition, three‐dimensional modelling of the 4BS mutation was carried out using a combination of Alphafold and PyMOL, and showed severe truncation of the HppS protein and likely a loss of function (Figure S5b). This provided strong evidence for loss of HppS being the causative agent for the rescued phenotype. To confirm this, we created a single gene knockout of *hppS* in the ∆ *croRS* strain to generate an *E. faecalis* ∆ *hppS* ∆ *croRS* double knockout. MIC and MBC assays subsequently demonstrated recovery of AMT in the ∆ *croRS* strain through a loss of HppS function; with a 1‐fold increase in vancomycin MIC and ≥5‐fold increase in MBC, as well as, a 2‐fold increase in bacitracin MIC and MBC compared to ∆ *croRS*, confirming that the *hppS* mutations were indeed responsible for the recovered phenotype in the suppressor mutants of the ∆ *croRS* strain. This apparent central role of HppS in the physiology of ∆ *croRS* correlates with our observations from the RNA‐seq experiments, which showed CroRS‐mediated induction of the MVA pathway as well as differential expression of the branching downstream pathways, cell wall biosynthesis (upregulated in the WT compared to ∆ *croRS*) and respiration (downregulated in the WT compared to ∆ *croRS*), in response to TXB.

Mutations in DNA‐directed RNA polymerase subunits have previously been associated with changes in antimicrobial susceptibility in enterococci (Du et al., 2014; Enne et al., 2004). Traditionally, mutations in *rpoB* are associated with rifampicin resistance, however, they, as well as mutations in *rpoC*, can also alter intrinsic resistance to cell wall‐targeting antimicrobials such as cephalosporins and daptomycin in *E. faecalis* (Du et al., 2014; Kristich & Little, 2012; Li et al., 2021). Uniquely, we have identified a mutation in *rpoE* in the 3BS mutant, and hypothesise that this may contribute to the recovery of tolerance to TXB and vancomycin in the ∆ *croRS* background (Table 2).

EF2367 encodes a putative *N*‐acetylmuramoyl‐L‐amidase autolysin. Autolysins play an essential role in normal cell wall turnover, and their absence has been associated with increased tolerance to the β‐lactam antimicrobials penicillin and amoxicillin in enterococci (Bravetti et al., 2009; Dubée et al., 2011; Hodges et al., 1992; Tomasz et al., 1970). In the RNA‐seq analyses above, we found that CroRS upregulates the expression of teichoic acid biosynthesis genes, a known target of TXB and regulator of cell wall autolytic activity in response to antimicrobial stress (Darnell et al., 2022; Fabretti et al., 2006). Therefore, it is tempting to speculate that in the absence of CroRS, teichoic acids are depleted and autolytic activity is dysregulated as a consequence. This may contribute to the observed increase in sensitivity to cell wall‐acting antimicrobials in the ∆ *croRS* strain. In this scenario, loss of function mutations in putative autolysins, such as those observed in EF2367 in the 1BS and 5BS mutants, may reduce autolytic activity and subsequently confer the decrease in antimicrobial susceptibility observed in these mutants compared to the parental ∆ *croRS* strain.

The phosphate ABC transporter, Pst, is a high‐affinity transport system with a putative role in the uptake of phosphate under nutrient stress (Moreno‐Letelier et al., 2011). In *E. faecalis*, the *pst* operon consists of five *pst* genes (*pstS*, *C*, *A*, *BA* and *BB*) and *phoU*. In *E. faecium*, a S199L amino acid substitution in PstBB confers protection against killing by vancomycin and chlorhexidine co‐treatment (Bhardwaj et al., 2021), while in *S. pneumoniae* deletion of *pstB* results in a decrease in uptake of phosphate and reduced autolysis, likely due to altered amidase activity (Novak et al., 1999). Perhaps, then it is not a coincidence that we observed an amino acid substitution in PstBB alongside a mutation in the putative autolysin, *N*‐acetylmuramoyl‐L‐amidase, as both may be working together to reduce autolytic activity and rescue tolerance in the ∆ *croRS* 5BS mutant (Table 2).

### 2.5 Antimicrobial susceptibility and cell physiology are intricately linked through the cell envelope stress response and isoprenoid biosynthesis in *E. faecalis*

**FIGURE 3:**
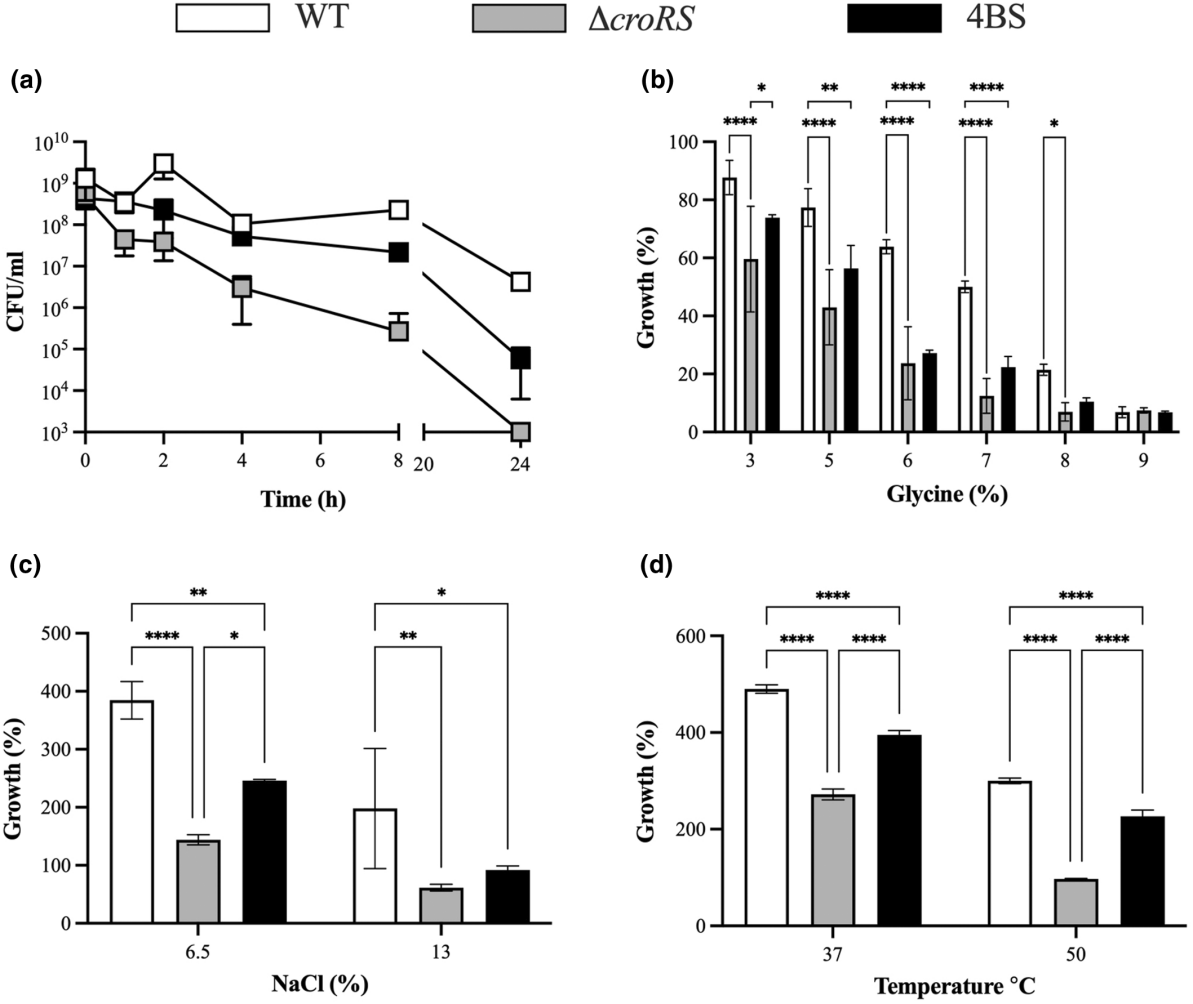
Phenotypic characterisation of the WT, ∆ *croRS* and 4BS strains. Cell stress assays were used to characterise the *Enterococcus faecalis* JH2‐2 WT (white), ∆ *croRS* (grey) and 4BS mutant (black). In the TXB time‐kill assay Strains were grown to mid‐exponential phase and challenged with teixobactin (25 × MIC) for 24 h. Cell survival was determined at time = 1, 2, 4, 8 and 24 h post‐challenge (a). (b) Overnight cultures were diluted 1/400 and challenged with a range of glycine concentrations (0, 3, 5, 6, 7, 8, 9%). Growth (%) was determined after 24 h and normalised to an untreated control. (c, d) Overnight cultures were diluted to an OD_600_ of 0.05 and challenged with osmotic (NaCl; 6.5% and 13%) (c) and temperature stress (37 and 50°C) (d). Growth (%) was determined after 24 h and normalised to an untreated control. All experiments were carried out in at least biological triplicate and are presented as the mean ± SD. A two‐way anova was used to determine statistical significance *p* =< 0.05 (*<0.05, ***<0.001, ****<0.0001).

Alterations in cell physiology are known to influence AMT of individual cells in heterogenous sub‐populations such as persisters (Brauner et al., 2016; Fridman et al., 2014; Levin‐Reisman et al., 2017). While mutations in *rpoE*, EF2367 and *pstBB* can be plausibly linked to the rescue of the ∆ *croRS* strain based on their predicted functions, causation cannot yet be established as they did not occur on their own in our suppressor mutants. However, the mutations observed in *hppS* did occur individually in two of our strains, allowing us to explore their role in AMT in more detail. Therefore, to determine the relationship between whole‐population tolerance and cell physiology, the *E. faecalis* WT, ∆ *croRS* and the HppS‐defective 4BS mutant strain were further characterised. A TXB time‐kill assay was carried out at 25 × MIC (50 μg/mL) over 24 h to quantify tolerance. After 24 h, strong killing (6‐log_10_ decrease) of the ∆ *croRS* strain was observed, while the WT and 4BS mutant were reduced only 2 and 4‐log_10_ respectively (Figure 3a). This suggests that the observed truncation of HppS in the 4BS mutant can partially rescue tolerance to TXB in the context of a ∆ *croRS* deletion strain. Interestingly, this intermediate phenotype was also observed in growth assays carried out in the presence of various cell envelope stressors, such as glycine stress (generating perturbation of the cell envelope), osmotic stress (NaCl) and temperature stress (50°C) (Figure 3b–d). These findings suggest a general role for isoprenoid biosynthesis in the cell envelope stress response.

**FIGURE 4:**
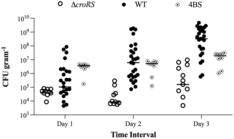
Colonisation of the *Manduca sexta* caterpillar by *Enterococcus faecalis* WT, ∆ *croRS* and 4BS strains. Each caterpillars was orally inoculated with 1 × 10^8^ CFU of *E. faecalis* and monitored for colonisation for 3 days using faecal pellets (viable counts expressed as CFU/g). Each data point is representative of an individual caterpillar with *n* ≥ 8 for each strain. Data are presented as an interleaved median scatter plot.

CroRS has been reported to have a potential defensive role against killing by antimicrobial peptides involved in insect immunity (Wadhawan et al., n.d.). To further investigate this, we placed the WT, ∆ *croRS* and 4BS strains into a lepidopteran gut colonisation model using caterpillars of the tobacco hornworm moth, *Manduca sexta*. It was shown previously that oral administration of *E. faecalis* to *M. sexta* led to the establishment of the bacteria in the insect gut without causing disease (Mason et al., 2011). We here exploited this observation by feeding the caterpillars with defined doses of each strain and monitoring enterococcal burdens in their faeces over time as an indication of colonisation success. We observed expansion of the WT strain over 3 days post‐inoculation, while the ∆ *croRS* strain established itself much more slowly (Figure 4). Importantly, while a slower increase in CFU/mL was to be expected given the slower growth rate of this strain, final numbers remained approximately 3‐log_10_ below those of the WT, indicating a severe defect in insect gut colonisation. As observed for the other stress phenotypes, the 4BS suppressor strain showed partially restored colonisation, intermediate of the ∆ *croRS* and WT strains (Figure 4). These data suggest that CroRS is indeed required for full colonisation of an insect gut and that the colonisation defect of the *croRS* mutant is at least in part due to issues with isoprenoid biosynthesis.

**FIGURE 5:**
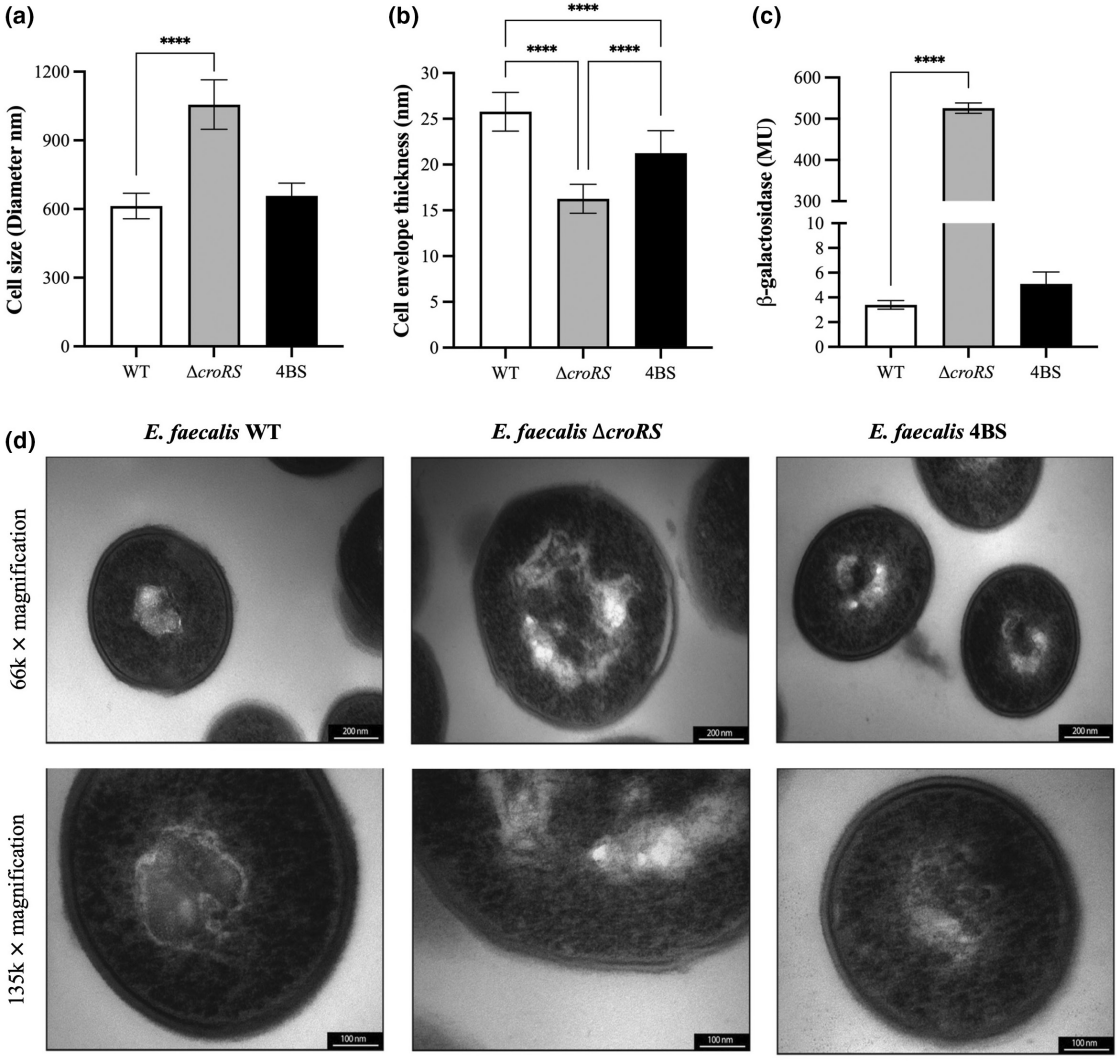
Analysis of the cell envelope stress response in the *Enterococcus faecalis* WT, ∆ *croRS* and 4BS strain using transmission electron microscopy and the PliaX reporter construct. Strains were grown to mid‐exponential phase under normal growth conditions and processed for transmission electron microscopy (TEM) (d). TEM images were captured at 66 or 135 k × magnification. Following TEM imaging cells were analysed for cell size (a) and cell envelope thickness (b) using ImageJ. The promoter region of LiaX (regulated by LiaFSR) was fused to *lacZ* and introduced into all three strains. Each strain was then assayed for *β*‐galactosidase activity in the absence of antimicrobial induction, expressed in Miller units (MU) (c). A one‐way anova was used to determine statistical significance; *p* = ****<0.001.

Given the regulatory role of CroRS on cell envelope biogenesis and the general sensitivity of the *croRS* deletion strain to cell wall‐compromising agents, we observed the WT, ∆ *croRS* and 4BS strains under the transmission electron microscope. Our first observation was that the ∆ *croRS* strain had a significantly larger cell size (46% larger than WT) but thinner cell envelope than the WT (Figure 5a,b,d). Shortages in peptidoglycan precursors have been shown to cause the elongation of cells, reminiscent of ∆ *croRS* (Figure 5d) (Dewachter et al., 2022). This supports our hypothesis that loss of CroRS impacts cell envelope integrity. Interestingly, the 4BS isolate showed a significant reduction in cell size (37% smaller than ∆ *croRS*) compared to the ∆ *croRS* strain, and was comparable to the WT (14% larger than WT) (Figure 5a), as well as a significant increase in cell envelope thickness compared to ∆ *croRS* (Figure 5b). However, the latter was not fully restored to WT, perhaps explaining the intermediate physiology observed in the stress analyses (Figures 3 and 5). Because of this observation, we also wanted to check whether this held true by analysing the CFU/mL/OD_600_ ratio for each strain. We observed a lower CFU/mL/OD_600_ ratio for the ∆ *croRS*, while the 4BS mutant displayed a similar ratio to WT, consistent with the larger size of the ∆ *croRS* strain (Figure S6). These data provide a critical link between CroRS, isoprenoid biosynthesis and cell envelope biogenesis, and offer a possible explanation for the impact of CroRS activity on the cell's physiology and the pleiotropic phenotype of the Δ*croRS* strain.

The transcriptomic and phenotypic evidence presented here suggests that the ∆ *croRS* strain is in a cell envelope‐stressed state even in the absence of antimicrobials, and that the compromised cell envelope integrity of the ∆ *croRS* strain can be rescued by a loss of HppS function (Figures 3 and 5 and Figure S3). If this hypothesis is correct, we should be able to detect this using a reporter assay for cell envelope stress. LiaFSR is an alternative two‐component system in *E. faecalis*, which is well‐known to respond to general perturbations in the cell envelope (Hancock & Perego, 2004). To test for cell envelope stress, a transcriptional reporter of its target promoter, P_*liaX*_, and the *lacZ* gene were developed and used to transform the ∆ *croRS* strain alongside the WT and 4BS suppressor mutant (Khan et al., 2019). In the absence of antimicrobial stress, the WT strain displayed very low P_*liaX*_‐*lacZ* activity, as expected, while the ∆ *croRS* strain showed very high constitutive activity (>150‐fold increase) (Figure 5c), a clear indicator that this strain is indeed in a state of constant cell envelope stress due to the loss of CroRS. Interestingly, truncation of HppS in the 4BS suppressor mutant was sufficient to reduce this stress to near WT levels (Figure 5c). Taken together these results highlight the integral role of CroRS as a master regulator of cell envelope biosynthesis to maintain cell envelope integrity and confer AMT.

## 3 CONCLUSION

CroRS is a two‐component system that regulates the cell envelope stress response in enterococci, and we have previously identified CroRS as an essential regulator of AMT in *E. faecalis* (Darnell et al., 2019). Here, we have carried out whole‐genome transcriptomics to identify key genes and pathways differentially expressed by CroRS in response to antimicrobial challenge and begin to unravel the role of CroRS in AMT as well as normal cell function. We show that CroRS plays a critical role in maintaining cell homeostasis, acting as a master regulator of cell envelope biosynthesis. We observe CroRS‐mediated upregulation of lipid II biosynthesis during normal cell growth (i.e. absence of TXB), and upregulated expression of genes involved in the biosynthesis of lipid II precursors, that is, the mevalonate pathway, polymerisation and integration of lipid II/peptidoglycan into the peptidoglycan matrix, that is, penicillin‐binding proteins, synthesis of cell wall polysaccharides (Epa) and teichoic acids, as well as the cell membrane, that is, fatty acids, in response to TXB challenge. As a consequence, loss of CroRS compromises cell envelope integrity, resulting in enlarged cell size, constant cell envelope stress and a dramatic increase in susceptibility to all tested cell‐envelope stressing agents. This is an interesting observation as CroRS is notably absent in other Gram‐positive species such as *S. aureus* and *B. subtilis*; but does have sequence similarity (30%) to WalRK/VicRK, an essential two‐component system known to have a major role in cell envelope maintenance that is found in *E. faecalis* as well as *S. aureus* and *B. subtilis* (Dubrac et al., 2007, 2008; Takada et al., 2018). Therefore, perhaps the addition of CroRS in *E. faecalis* allows for a higher degree of resistance and tolerance to cell wall‐acting antimicrobials that is not observed in these other species.

**FIGURE 6:**
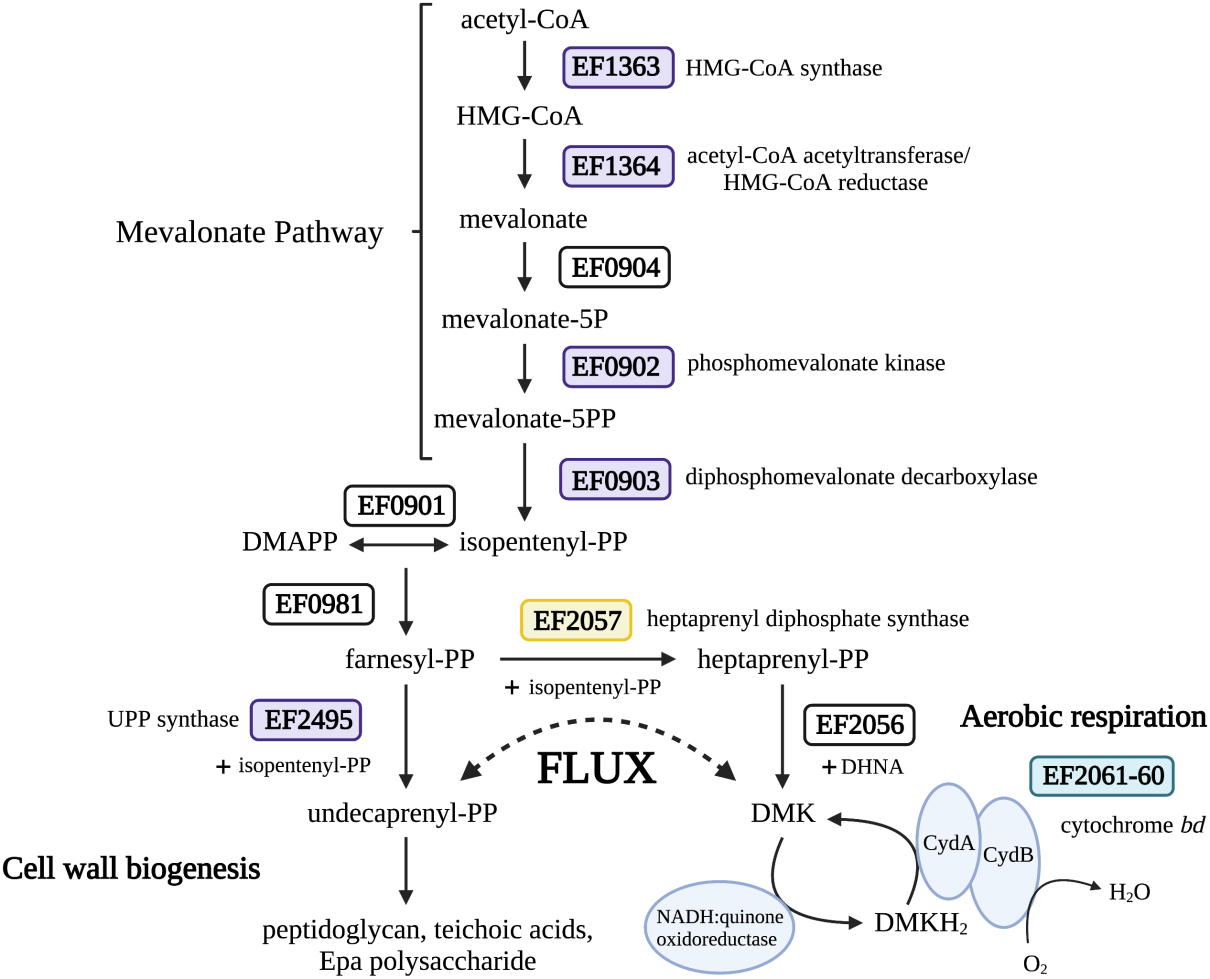
CroRS regulates isoprenoid flux between cell wall biogenesis and aerobic respiration to maintain cell wall homeostasis in response to antimicrobial stress. In wild‐type *Enterococcus faecalis* CroRS confers tolerance to TXB‐induced cell killing by regulating isoprenoid flux using a dual mechanism. In response to TXB challenge, we hypothesise CroRS simultaneously reduces the capacity for aerobic respiration, and thus the demand for isoprenoids/demethylmenaquinone (DMK), by decreasing the expression of cytochrome *bd* (green box; CydA, CydB), while upregulating genes (purple boxes) involved in isoprenoid biosynthesis (i.e. mevalonate pathway and UPP synthase) and cell wall biogenesis (i.e. peptidoglycan, teichoic acids and Epa polysaccharide). In the absence of *croRS*, *E. faecalis* is susceptible to killing by TXB. However, proposed loss of function mutations in a heptaprenyl diphosphate synthase (yellow box; HppS) can partially rescue this phenotype. This supports our hypothesis that TXB tolerance can be conferred in *E. faecalis* through the control of isoprenoid flux from aerobic respiration to cell wall biogenesis.

In addition, we show that a loss of function in a heptaprenyl diphosphate synthase can recover all facets of the ∆ *croRS* phenotype, with the exception of resistance, and we propose a model where CroRS acts as a gate‐keeper between the two branches of isoprenoid biosynthesis, controlling the flux of isoprenoids needed for cell wall synthesis and respiration, to maintain cell wall homeostasis upon antimicrobial challenge (Figure 6). We hypothesise that CroRS upregulates MVA pathway flux to provide sufficient production of the precursors FPP and IPP for synthesis of the essential lipid carrier UPP and to support the increased need for cell wall biosynthesis. Conversely, we hypothesise CroRS downregulates cytochrome *bd* expression, as evidenced in the transcriptome data, to reduce the demand for DMK and its precursors FPP and IPP, thereby driving isoprenoid flux towards UPP biosynthesis upon antimicrobial stress. In this light, rescue of the ∆ *croRS* phenotype through suppressor mutations in or deletion of *hppS*, encoding a key enzyme in DMK biosynthesis, illustrates the integral role of isoprenoid flux in CroRS‐mediated AMT. In the absence of the CroRS‐mediated adjustments to UPP synthesis and cytochrome *bd* expression proposed above, loss of HppS activity may lead to similar overall effects by directing isoprenoid flux away from DMK synthesis and towards UPP production. Interestingly, while mutations in *hppS* corrected for defects in growth and survival against agents that compromise the cell envelope (i.e. AMT), they could not counteract the inhibitory effects of antimicrobials on cell proliferation (i.e. antimicrobial resistance). While we currently cannot explain the increased sensitivity to growth inhibition, an answer might be found in previous observations that a decrease in lipid II‐content of the cell can lead to modest increases in vancomycin resistance, because less target is available for the antibiotic (Lee & Helmann, 2013). In fact, it has been proposed that minimising target exposure during cell wall synthesis can lead to intrinsic antibiotic resistance (Piepenbreier et al., 2019). In this light, one might speculate that the increased isoprenoid flux towards UPP could lead to an overall increase in lipid II cycle intermediates, and thus offer a potential explanation for the increased sensitivity of the HppS‐deficient strains compared to their CroRS‐negative parent. Taken together, the observed difference in recovery of tolerance but not resistance in the HppS‐deficient strains highlights a fundamental link between cell homeostasis and killing by antimicrobials, and by extension suggests that mechanisms of antimicrobial resistance and tolerance are functionally separate. Future work will endeavour to further deconstruct the relationship between tolerance and resistance and to identify the molecular role of isoprenoids in AMT in *E. faecalis*.

## 4 EXPERIMENTAL PROCEDURES

### 4.1 Bacterial growth/growth curves

All *E. faecalis* strains were routinely grown in BHI broth and agar overnight at 37°C with no agitation unless otherwise stated. All *Escherichia coli* strains were routinely grown in LB broth and agar at 37°C (200 r.p.m), unless otherwise stated. Cultures for RNA sequencing and optimisation were grown as previously described (Darnell et al., 2019). Growth was determined by optical density at 600 nm (OD_600_). TXB stocks were made with fresh dimethyl sulfoxide (DMSO) and stored at −20°C. All bacterial strains used in this study are listed in Table S7.

### 4.2 Antimicrobial susceptibility assays

Minimum inhibitory (MIC) and bactericidal (MBC) concentrations were carried out in cation‐adjusted Mueller‐Hinton broth and BHI agar as previously described, aligning with CLSI guidelines (Darnell et al., 2019). Time‐dependent kill assays were carried out to determine cell death kinetics over time as previously described (Darnell et al., 2019).

### 4.3 RNA extraction and preparation of RNA samples for sequence analysis

*Enterococcus faecalis* JH2‐2 and Δ*croRS* were grown in biological triplicate to mid‐exponential phase (OD_600_ of 0.5) at 37°C, 130 r.p.m. Cultures were subsequently split to produce two sets of biological triplicates. One set was challenged with 0.5 g/mL (0.25 × MIC) of TXB, while the other set was untreated controls. Cultures were grown for a further 1 h and harvested for RNA extraction. Total RNA was isolated using TRIzol‐chloroform extraction as previously described (Darnell et al., 2019). RNA samples were purified using a Zymo RNA clean and concentrate kit as per the manufacturer's instructions. RNA concentration and integrity (RIN > 8) were determined by bioanalyser.

### 4.4 RNA sequencing and gene expression analysis

#### 4.4.1 cDNA library preparation and sequencing of the *E. faecalis* JH2‐2 and ΔcroRS transcriptomes

RNA libraries were prepped using the Zymo‐Seq RiboFree Total RNA‐seq Library Kit. Sequencing was completed using an Illumina MiSeq (v3) system generating 150 bp paired‐end reads

#### 4.4.2 Analysis of RNA sequencing data

Adapter sequences were removed from raw fastq files using bbduk. Reads were aligned to the *E. faecalis* JH2‐2 genome (GenBank accession numbers NZ_KI1518257.1 and NZ_KI1518256.1) using Bowtie2. Statistical and principle‐component analyses were performed using the Bioconductor DESeq2 package. Parameters considered during analysis were the fold change (≥1.0‐fold log_2_), the mean number of reads (>50) and the adjusted *P* value (*p*_adj_ < 0.1). Genes were also annotated with the *E. faecalis* V583 reference (GenBank accession number NC_004668.1) gene homologue and ontology using KEGGRest. Gene annotations and ontology assignments were complemented with NCBI BLAST and literature searches when necessary

#### 4.4.3 Hypergeometric testing to determine gene ontology enrichment

Following the assignment of differentially expressed genes to KEGG pathways, a hypergeometric test was performed by comparing the number of genes within the regulon to the total number of genes within the respective *E. faecalis* V583 KEGG pathway. Pathways with a *p* < 0.05 were deemed significantly enriched.

### 4.5 qRT‐PCR of *E. faecalis* genes in response to TXB

RNA‐seq data were validated by qRT‐PCR with primers specific to 12 genes, 11 differentially expressed and 1 consitutively expressed gene, EF0013 (*dnaB*). Of the 11 differentially expressed, five were identified as CroRS‐regulated. Gene‐specific primers were designed using Primer 3 (v0.4.0) software (http://primer3.ut.ee/). Primer sequences can be found in Table S7, and primer concentrations were optimised prior to final validation. Following RNA extraction and DNAse treatment, cDNA was synthesised using a Superscrpit™ III IV VILO™ Master Mix (Invitrogen) according to the manufacturer's instructions. cDNA was subsequently purified using the Zymo DNA Clean and Concentrator® kit and stored at −80°C. qRT‐PCR was carried out using a ViiA 6 real‐time PCR system with the PowerUp™ SYBR™ Green Master Mix (Applied Biosystems) in accordance with the manufacturer's instructions. Differential gene expression was determined using the change in threshold cycle (Δ*C*_*T*_) values and normalised using the constitutively expressed EF0013.

### 4.6 Serial passaging of ∆croRS for wild‐type growth

To isolate suppressor mutants with improved fitness compared to the *croRS* deletion strain, we utilised a serial passaging method. Five independent overnight cultures were inoculated 1:1000 into 10 mL BHI medium and incubated at 37°C with no aeration. Cultures were passaged every 48 h under the same conditions. Cells were serially diluted and plated every three passages onto BHI and bile‐esculin medium to ensure no contamination had occurred. The experiment was concluded upon visible confirmation of improved fitness, assessed as increased turbidity of overnight cultures, which appeared within 10–14 days.

### 4.7 Generation of an *E. faecalis* ∆hppS ∆croRS double mutant

To generate the *E. faecalis* ∆ *hppS* ∆ *croRS* double mutant, we first had to generate the hppS_pIMAYZ knockout plasmid. Flanking regions up‐ and downstream of the *hppS* gene (EF2057) were amplified using the primers EF2057_AF, BR, CF and DR (Table S7) and cloned into the pIMAYZ knockout vector as previously described (Darnell et al., 2019). The pIMAYZ_EF2057 knockout plasmid was transformed into electrocompetent *E. faecalis* ∆ *croRS* and the *hppS* gene subsequently knocked out through homologous recombination (Darnell et al., 2019). Double deletion mutants were confirmed by whole‐genome sequencing (WGS).

### 4.8 Whole‐genome sequencing analysis

#### 4.8.1 Serial passaging

Genomic DNA was extracted from *E. faecalis* strains using the GeneJET Genomic DNA Purification Kit. Genomic DNA was WGS by SeqCenter (formerly MiGs) in Pittsburgh, PA, USA. Sample libraries were prepared using the Illumina DNA Prep kit and IDT 10 bp UDI indices and sequenced on an Illumina NextSeq 2000, producing 2 × 151 bp reads at ~60 × coverage. Demultiplexing, quality control and adapter trimming were performed with bcl‐convert (v3.9.3). Sequences were assembled de novo using Spades version 3.15.3, available at https://github.com/ablab/spades. Single‐nucleotide polymorphisms were called using Snippy version 4.5.0, available at https://github.com/tseeman/snippy/, with default parameters, through the Cloud Infrastructure for Microbial Bioinformatics (CLIMB), using the *E. faecalis* Δ*croRS* sequence as a reference index. Gene function predictions were obtained using NCBI BlastN (Altschup et al., 1990) against the *E. faecalis* JH2‐2 wild‐type sequence, annotated using Prokka version 1.14.5, available at https://githib.com/tseeman/prokka. In the absence of annotation, a BlastP search of the sequence against the NCBI database was performed.

### 4.9 Confirmation of the hppS gene knockout in the *E. faecalis* ΔcroRS strain

Genomic DNA was extracted from the *E. faecalis* ∆ *hppS* ∆ *croRS* double knockout and sent for WGS at SeqCenter. WGS was performed as written above. WGS analysis was carried out using Geneious Prime 2022.0.2 (https://www.geneious.com) software. Illumina reads were paired using the inbuilt ‘set‐paired reads’ function, and then trimmed using the ‘bbduk’ plugin in Geneious. Reads were error corrected and normalised using the inbuilt ‘Error correct and normalise’ function to reduce file size and increase mapping efficiency in Geneious. Reads were mapped to the *E. faecalis* JH2‐2 (NZ_KI18256 and NZ_KI18257) wild‐type reference sequence using the mapper plugin Bowtie2 within the Geneious function ‘Map to Reference’. Gene deletions were visually confirmed via lack of reads aligning to the *croR*, *croS* and *hppS* genes. The inbuilt Geneious function ‘Find Variations and SNPS’ was also used to ensure no additional variations/SNPs had been produced during mutant construction.

### 4.10 3D modelling of the 4BS HppS mutation in PyMOL

To visualise the impact of the E31* truncation in HppS in the 4BS mutant, we generated a putative structure of HppS (EF2057; UniProt ID: Q833B0) using Alphafold (Jumper et al., 2021; Varadi et al., 2021). The predicted structure was then downloaded as a PBP file and analysed in PyMOL (The PyMOL Molecular Graphics System, Version 2.0 Schrödinger, LLC; https://pymol.org/2/support.html).

### 4.11 General stress testing

#### 4.11.1 Temperature stress

In biological triplicate, overnight cultures were normalised to an OD_600_ of 0.5 and inoculated 1/10 into fresh BHI broth to a final OD_600_ 0.05. Cells were grown at 37 and 50°C overnight with no aeration. Following incubation, cell growth was measured by optical density (600 nm) and calculated as a percentage comparison of before and after temperature challenge.

#### 4.11.2 Osmotic (NaCl) stress

In biological triplicate, overnight cultures were pelleted by centrifugation (10,000 ***g*** for 5 min) and washed in 1 × phosphate saline buffer. Cells were resuspended in BHI + NaCl (6.5% or 13%) to an OD_600_ of 0.05 and grown overnight at 37°C with no aeration. Following incubation, cell growth was measured by optical density (600 nm) and calculated as a percentage comparison of before and after osmotic challenge.

#### 4.11.3 Cell wall (glycine) stress

In biological triplicate, overnight cultures were diluted 1/200 in M17 medium with 0.5 M sucrose broth with glycine at concentrations of 0, 3, 5, 6, 7, 8 and 9% (w/v). Cultures were grown overnight at 37°C with no agitation. Cell growth was measured by optical density (600 nm) and calculated as a percentage comparison to the untreated control (0% glycine).

### 4.12 Colonisation of the *Manduca sexta* caterpillar by *E. faecalis*

*Manduca sexta* caterpillars were grown by the University of Bath *Manduca sexta* facility to the early fifth instar and subsequently inoculated, by direct pipetting into the mouth opening, with 10 μL of 1 × 10^10^ CFU/mL *E. faecalis* suspended in sterile saline, to achieve an inoculum per caterpillar of 1 × 10^8^ CFU. A total of at least 8 individual caterpillars were inoculated for each strain of *E. faecalis*. *M. sexta* were kept in individual containers at 37°C with a 12 h light/dark photoperiod for 3 days post‐inoculation. The weight of the caterpillars was monitored daily to ensure health, and data from caterpillars who stopped gaining weight were excluded from further analysis as this indicated disease. To monitor colonisation over time, faecal pellets were cleared from the caterpillar containers each morning, and fresh pellets were collected approximately 4–6 h later to define a consistent time window. Faecal pellets were weighed and resuspended in 10 volumes of sterile saline. Serial dilutions were then plated onto Bile Esculin agar (Merck), incubated overnight at 37°C and enterococci enumerated by counting of black colonies. Colonisation was reported as CFU enterococci per gram faecal pellets.

### 4.13 Transmission electron microscopy of *E. faecalis* WT, ∆croRS and 4BS strains

*Enterococcus faecalis* strains were grown to mid‐exponential phase (OD_600_ of 0.5, 1 × 10^8^ CFU ml^−1^) in fresh BHI broth and harvested by centrifugation (3000 r.p.m, 4°C, 6 min). Cell pellets were resuspended in a primary fixative solution (5% glutaraldehyde in 0.1 M cacodylate buffer) for 30 min at room temperature. Cells were subsequently washed 3 × in 0.1 M cacodylate buffer and incubated with a secondary fixative solution (1% osmium and 1% potassium ferricyanide in 0.1 M cacodylate buffer) for 30 min. Following the application of the secondary fixative, cells were washed 3 × in 0.1 M cacodylate buffer and stored in the refrigerator for further processing. Fixed cells were washed with ddH_2_O and dehydrated with ethanol at progressively increasing concentrations: 30% ethanol for 5 min, 50% ethanol for 5 min, 70% ethanol for 5 min and 95% ethanol for 5 min, followed by three treatments with 100% ethanol for 10, 15 and 20 min respectively. The dehydration process was completed by 2 × 15 min treatments with propylene oxide. Finally, cells were treated for 2 h with a 1:1 mixture of resin and propylene oxide, then left in resin overnight. Following this incubation, cells were embedded in fresh EmBed 812 resin and polymerised at 60°C for 48 h. Ultrathin sectioning (85 nm) was carried out using a diamond knife on a Leica EM UC7 ultramicrotome and collected on formvar‐coated copper grids. Sections were stained with uranyl acetate and lead citrate and investigated using a Philips CM100 BioTWIN transmission electron microscope (Philips/FEI Corporation) fitted with a MegaView III digital camera (Soft Imaging System GmbH).

### 4.14 Construction of the liaRS promoter‐lacZ plasmid

The *liaX* transcriptional promoter fusion to *lacZ* in *E. faecalis* was constructed in the vector pTCVlac (Poyart & Trieu‐Cuot, 1997). The *liaX* promoter fragment was amplified with the primers PliaX_F and PliaX_R (Table S7). The fragment was then inserted into pTCVlac via the EcoRI and BamHI restriction sites. The resulting plasmid pTCVlac_PliaX was transformed into electrocompetent *E. faecalis* WT, ∆ *croRS* and 4BS strains by electroporation (Table S7).

### 4.15 *β*‐galactosidase assays

To quantitatively assess the induction of the P*liaX*‐ reporter construct in *E. faecalis*, cells were grown to mid‐exponential phase (OD_600_ 0.4–0.5) in BHI medium. Cells were harvested via centrifugation and stored at −20°C. *β*‐galactosidase activity was assayed in permeabilised cells and expressed in Miller units (MU). For this, cells were resuspended and normalised to an OD_600_ of 0.5 in Z‐buffer. From these cells, were diluted 1/5 and 1/2.5 in Z‐buffer and lysed by vortexing with 0.1% SDS (w/v) and chloroform. Cells were left to rest at room temperature for 5–10 min. Reactions were initiated by the addition of ONPG (*o*‐nitrophenyl‐*β*‐D‐galactopyranoside; 4 mg/mL in Z buffer) and monitored for yellow colouration. Reactions were monitored for colour change for a maximum of 20 min. Upon colour change, reactions were stopped by adding 1 M sodium carbonate and reaction time was noted. Absorbance at 420 nm (A420) was the read and MU was calculated using the following equation.

$$\text{Miller units}\;\left(\mathrm{MU}\right)=\frac{A420\times 1000}{\text{Time}\;\left(\text{minutes}\right)\times \text{volume of cells}\;\left(\text{in}\;\mathrm{mL}\right)\times \mathrm{OD}600}$$

#### ACCESSION NUMBER

The data from this article may be found at ArrayExpress under the accession number E‐MTAB‐12526.

## AUTHOR CONTRIBUTIONS

**Francesca O. Todd Rose:** Investigation; data curation; formal analysis. **Rachel L. Darnell:** investigation; data curation; formal analysis; conceptualization; supervision; funding acquisition; writing ‐ original draft; project administration. **Olivia E. Rose:** Investigation. **Olivia Paxie:** Investigation. **Georgia Campbell:** Methodology. **Gregory M. Cook:** conceptualization; supervision; funding acquisition. **Susanne Gebhard:** Conceptualization; investigation; supervision; resources; funding acquisition; writing – original draft; project administration.

## ETHICS STATEMENT

This article does not contain any studies with human participation or animals.

## Supporting information


Appendix S1.

## Data Availability

The data that support the findings of this study are openly available in Biostudies, ArrayExpress at https://www.ebi.ac.uk/biostudies/arrayexpress/studies/, reference number E‐MTAB‐12526.

## References

[mmi15128-bib-0001] Abranches, J. , Tijerina, P. , Avilés‐Reyes, A. , Gaca, A.O. , Kajfasz, J.K. & Lemos, J.A. (2013) The cell wall‐targeting antibiotic stimulon of *Enterococcus faecalis* . PLoS One, 8(6), e64875. Available from: 10.1371/journal.pone.0064875 23755154 PMC3670847

[mmi15128-bib-0002] Altschup, S.F. , Gish, W. , Miller, W. , Myers, E.W. & Lipman, D.J. (1990) Basic local alignment search tool. Journal of Molecular Biology, 215, 403–410. 2231712 10.1016/S0022-2836(05)80360-2

[mmi15128-bib-0003] Arbeloa, A. , Segal, H. , Hugonnet, J.E. , Josseaume, N. , Dubost, L. , Brouard, J.P. et al. (2004) Role of class A penicillin‐binding proteins in PBP5‐mediated beta‐lactam resistance in *Enterococcus faecalis* . Journal of Bacteriology, 186(5), 1221–1228. Available from: 10.1128/JB.186.5.1221 14973044 PMC344401

[mmi15128-bib-0004] Arias, C.A. , Panesso, D. , McGrath, D.M. , Qin, X. , Mojica, M.F. , Miller, C. et al. (2011) Genetic basis for *in vivo* daptomycin resistance in enterococci. New England Journal of Medicine, 365(10), 892–900. Available from: 10.1056/NEJMoa1011138 21899450 PMC3205971

[mmi15128-bib-0005] Arias, C.A. & Murray, B.E. (2012) The rise of the *Enterococcus*: beyond vancomycin resistance. Nature Reviews Microbiology, 10(4), 266–278. Available from: 10.1038/nrmicro2761 22421879 PMC3621121

[mmi15128-bib-0006] Bhardwaj, P. , Islam, M.Z. , Kim, C. , Nguyen, U.T. & Palmer, K.L. (2021) *ddcP*, *pstB*, and excess D‐lactate impact synergism between vancomycin and chlorhexidine against *Enterococcus faecium* 1,231,410. PLoS One, 16(4), e0249631. Available from: 10.1371/JOURNAL.PONE.0249631 33831063 PMC8031426

[mmi15128-bib-0007] Brauner, A. , Fridman, O. , Gefen, O. & Balaban, N.Q. (2016) Distinguishing between resistance, tolerance and persistence to antibiotic treatment. Nature Reviews Microbiology, 14, 320–330. Available from: 10.1038/nrmicro.2016.34 27080241

[mmi15128-bib-0008] Bravetti, A.‐L. , Mesnage, S. , Lefort, A. , Chau, F. , Eckert, C. , Garry, L. et al. (2009) Contribution of the autolysin AtlA to the bactericidal activity of amoxicillin against *Enterococcus faecalis* JH2‐2. Antimicrobial Agents and Chemotherapy, 53(4), 1667–1669. Available from: 10.1128/AAC.00692-08 19188384 PMC2663090

[mmi15128-bib-0009] Le Breton, Y. , Boël, G. , Benachour, A. , Prévost, H. , Auffray, Y. & Rincé, A. (2003) Molecular characterization of *Enterococcus faecalis* two‐component signal transduction pathways related to environmental stresses. Environmental Microbiology, 5(5), 329–337. Available from: 10.1046/j.1462-2920.2003.00405.x 12713459

[mmi15128-bib-0010] Comenge, Y. , Quintiliani, R., Jr. , Li, L. , Dubost, L. , Brouard, J.P. , Hugonnet, J.E. et al. (2003) The CroRS two‐component regulatory system is required for intrinsic beta‐lactam resistance in *Enterococcus faecalis* . Journal of Bacteriology, 185(24), 7184–7192. Available from: 10.1128/JB.185.24.7184-7192.2003 14645279 PMC296236

[mmi15128-bib-0011] Dale, J.L. , Cagnazzo, J. , Phan, C.Q. , Barnes, A.M.T. & Dunny, G.M. (2015) Multiple roles for *Enterococcus faecalis* glycosyltransferases in biofilm‐associated antibiotic resistance, cell envelope integrity, and conjugative transfer. Antimicrobial Agents and Chemotherapy, 59(7), 4094–4105. Available from: 10.1128/AAC.00344-15 25918141 PMC4468649

[mmi15128-bib-0012] Darnell, R.L. , Knottenbelt, M.K. , Todd Rose, F.O. , Monk, I.R. , Stinear, T.P. & Cook, G.M. (2019) Genomewide profiling of the *Enterococcus faecalis* transcriptional response to teixobactin reveals CroRS as an essential regulator of antimicrobial tolerance. mSphere, 4(3), e00228–e00219. Available from: 10.1128/mSphere.00228-19 31068434 PMC6506618

[mmi15128-bib-0013] Darnell, R.L. , Paxie, O. , Rose, F.O.T. , Morris, S. , Krause, A.L. , Monk, I.R. et al. (2022) Antimicrobial tolerance and its role in the development of resistance: lessons from enterococci. Advances in Microbial Physiology, 81, 25–65. Available from: 10.1016/BS.AMPBS.2022.06.004 36167442

[mmi15128-bib-0014] Desai, J. , Liu, Y.L. , Wei, H. , Liu, W. , Ko, T.P. , Guo, R.T. et al. (2016) Structure, function, and inhibition of *Staphylococcus aureus* heptaprenyl diphosphate synthase. ChemMedChem, 11, 1915–1923. Available from: 10.1002/cmdc.201600311 27457559 PMC5012948

[mmi15128-bib-0015] Dewachter, L. , Dénéréaz, J. , Liu, X. , de Bakker, V. , Costa, C. , Baldry, M. et al. (2022) Amoxicillin‐resistant *Streptococcus pneumoniae* can be resensitized by targeting the mevalonate pathway as indicated by sCRilecs‐seq. eLife, 11, e75607. Available from: 10.7554/ELIFE.75607 35748540 PMC9363119

[mmi15128-bib-0016] Djorić, D. , Little, J.L. & Kristich, C.J. (2020) Multiple low‐reactivity class B penicillin‐binding proteins are required for cephalosporin resistance in enterococci. Antimicrobial Agents and Chemotherapy, 64(4), e02273–e02219. Available from: 10.1128/AAC.02273-19 32041714 PMC7179317

[mmi15128-bib-0017] Du, X. , Hua, X. , Qu, T. , Jiang, Y. , Zhou, Z. & Yu, Y. (2014) Molecular characterization of Rifr mutations in *Enterococcus faecalis* and *Enterococcus faecium* . Journal of Chemotherapy, 26(4), 217–221. Available from: 10.1179/1973947813Y.0000000137 24070269

[mmi15128-bib-0018] Dubée, V. , Chau, F. , Arthur, M. , Garry, L. , Benadda, S. , Mesnage, S. et al. (2011) The in vitro contribution of autolysins to bacterial killing elicited by amoxicillin increases with inoculum size in *Enterococcus faecalis* . Antimicrobial Agents and Chemotherapy, 55(2), 910–912. Available from: 10.1128/AAC.01230-10 21098238 PMC3028817

[mmi15128-bib-0019] Dubrac, S. , Boneca, I.G. , Poupel, O. & Msadek, T. (2007) New insights into the WalK/WalR (YycG/YycF) essential signal transduction pathway reveal a major role in controlling cell wall metabolism and biofilm formation in *Staphylococcus aureus* . Journal of Bacteriology, 189(22), 8257–8269. Available from: 10.1128/JB.00645-07/ASSET/B68A5266-A7A1-41C1-B9E5-E8E91BACE82A/ASSETS/GRAPHIC/ZJB0220772950010.JPEG 17827301 PMC2168699

[mmi15128-bib-0020] Dubrac, S. , Bisicchia, P. , Devine, K.M. & Msadek, T. (2008) A matter of life and death: cell wall homeostasis and the WalKR (YycGF) essential signal transduction pathway. Molecular Microbiology, 70(6), 1307–1322. Available from: 10.1111/J.1365-2958.2008.06483.X 19019149

[mmi15128-bib-0021] Enne, V.I. , Delsol, A.A. , Roe, J.M. & Bennett, P.M. (2004) Rifampicin resistance and its fitness cost in *Enterococcus faecium* . The Journal of Antimicrobial Chemotherapy, 53(2), 203–207. Available from: 10.1093/JAC/DKH044 14688044

[mmi15128-bib-0022] Fabretti, F. , Theilacker, C. , Baldassarri, L. , Kaczynski, Z. , Kropec, A. , Holst, O. et al. (2006) Alanine esters of enterococcal lipoteichoic acid play a role in biofilm formation and resistance to antimicrobial peptides. Infection and Immunity, 74(7), 4164–4171. Available from: 10.1128/IAI.00111-06/ASSET/AA98DE38-8559-4645-8821-A60FB6B4368B/ASSETS/GRAPHIC/ZII007066036006B.JPEG 16790791 PMC1489678

[mmi15128-bib-0023] Fridman, O. , Goldberg, A. , Ronin, I. , Shoresh, N. & Balaban, N.Q. (2014) Optimization of lag time underlies antibiotic tolerance in evolved bacterial populations. Nature, 513(7518), 418–421. Available from: 10.1038/nature13469 25043002

[mmi15128-bib-0024] Gilmore, M.S. , Salamzade, R. , Selleck, E. , Bryan, N. , Mello, S.S. , Manson, A.L. et al. (2020) Genes contributing to the unique biology and intrinsic antibiotic resistance of *Enterococcus faecalis* . mBio, 11(6), 1–28. Available from: 10.1128/MBIO.02962-20 PMC7701990 33234689

[mmi15128-bib-0025] Guerardel, Y. , Sadovskaya, I. , Maes, E. , Furlan, S. , Chapot‐Chartier, M.P. , Mesnage, S. et al. (2020) Complete structure of the enterococcal polysaccharide antigen (EPA) of vancomycin‐resistant *Enterococcus faecalis* V583 reveals that EPA decorations are teichoic acids covalently linked to a rhamnopolysaccharide backbone. mBio, 11(2), e00277–e00220. Available from: 10.1128/MBIO.00277-20/SUPPL_FILE/MBIO.00277-20-ST003.PDF 32345640 PMC7188991

[mmi15128-bib-0026] Hancock, L.E. & Perego, M. (2004) Systematic inactivation and phenotypic characterization of two‐component signal transduction systems of *Enterococcus faecalis* V583. Journal of Bacteriology, 186(23), 7951–7958. Available from: 10.1128/JB.186.23.7951 15547267 PMC529088

[mmi15128-bib-0027] Heuston, S. , Begley, M. , Gahan, C.G.M. & Hill, C. (2012) Isoprenoid biosynthesis in bacterial pathogens. Microbiology, 158, 1389–1401. Available from: 10.1099/mic.0.051599-0 22466083

[mmi15128-bib-0028] Hodges, T.L. , Zighelboim‐Daum, S. , Eliopoulos, G.M. , Wennersten, C. & Moellering, R.C., Jr. (1992) Antimicrobial susceptibility changes in *Enterococcus faecalis* following various penicillin exposure regimens. Antimicrobial Agents and Chemotherapy, 36(1), 121–125. Available from: 10.1128/AAC.36.1.121 1590676 PMC189238

[mmi15128-bib-0029] Homma, T. , Nuxoll, A. , Gandt, A.B. , Ebner, P. , Engels, I. , Schneider, T. et al. (2016) Dual targeting of cell wall precursors by teixobactin leads to cell lysis. Antimicrobial Agents and Chemotherapy, 60, 6510–6517. Available from: 10.1128/AAC.01050-16 27550357 PMC5075054

[mmi15128-bib-0030] Jumper, J. , Evans, R. , Pritzel, A. , Green, T. , Figurnov, M. , Ronneberger, O. et al. (2021) Highly accurate protein structure prediction with AlphaFold. Nature, 596, 583–589. Available from: 10.1038/s41586-021-03819-2 34265844 PMC8371605

[mmi15128-bib-0031] Kellogg, S.L. , Little, J.L. , Hoff, J.S. & Kristich, C.J. (2017) Requirement of the CroRS two‐ component system for resistance to cell wall‐targeting antimicrobials in *Enterococcus faecium* . Antimicrobial Agents Chemotherapy, 61(5), e02461–e02416. Available from: 10.1128/AAC.02461-16 28223383 PMC5404561

[mmi15128-bib-0032] Kellogg, S.L. & Kristich, C.J. (2016) Functional dissection of the CroRS two‐component system required for resistance to cell wall stressors in *Enterococcus faecalis* . Journal of Bacteriology, 198(8), 1326–1336. Available from: 10.1128/JB.00995-15/ASSET/043B3C2C-B280-4578-BDCD-A480F1FE6324/ASSETS/GRAPHIC/ZJB9990940110007.JPEG 26883822 PMC4859583

[mmi15128-bib-0033] Khan, A. , Davlieva, M. , Panesso, D. , Rincon, S. , Miller, W.R. , Diaz, L. et al. (2019) Antimicrobial sensing coupled with cell membrane remodeling mediates antibiotic resistance and virulence in *Enterococcus faecalis* . Proceedings of the National Academy of Sciences of the United States of America, 116(52), 26925–26932. Available from: 10.1073/PNAS.1916037116/SUPPL_FILE/PNAS.1916037116.SAPP.PDF 31818937 PMC6936494

[mmi15128-bib-0034] Kohanski, M.A. , Dwyer, D.J. & Collins, J.J. (2010) How antibiotics kill bacteria: from targets to networks. Nature Reviews Microbiology, 8, 423–435. Available from: 10.1038/nrmicro2333 20440275 PMC2896384

[mmi15128-bib-0035] Korir, M.L. , Dale, J.L. & Dunny, G.M. (2019) Role of *epaQ*, a previously uncharacterized *Enterococcus faecalis* gene, in biofilm development and antimicrobial resistance. Journal of Bacteriology, 201, e00078–e00019. Available from: 10.1128/JB.00078-19 30910809 PMC6707930

[mmi15128-bib-0036] Kristich, C.J. & Little, J.L. (2012) Mutations in the β subunit of RNA polymerase alter intrinsic cephalosporin resistance in enterococci. Antimicrobial Agents and Chemotherapy, 56(4), 2022–2027. Available from: 10.1128/AAC.06077-11/ASSET/E61899FB-0EDB-42BE-80A8-989641F83C8A/ASSETS/GRAPHIC/ZAC9991007380001.JPEG 22290974 PMC3318385

[mmi15128-bib-0037] Lee, Y.H. & Helmann, J.D. (2013) Reducing the level of undecaprenyl pyrophosphate synthase has complex effects on susceptibility to cell wall antibiotics. Antimicrobial Agents and Chemotherapy, 57(9), 4267–4275. Available from: 10.1128/AAC.00794-13/FORMAT/EPUB 23796923 PMC3754353

[mmi15128-bib-0038] Levin‐Reisman, I. , Ronin, I. , Gefen, O. , Braniss, I. , Shoresh, N. & Balaban, N.Q. (2017) Antibiotic tolerance facilitates the evolution of resistance. Science, 355, 826–830. Available from: 10.1126/science.aaj2191 28183996

[mmi15128-bib-0039] Li, L. , Higgs, C. , Turner, A.M. , Nong, Y. , Gorrie, C.L. , Sherry, N.L. et al. (2021) Daptomycin resistance occurs predominantly in vanA‐type vancomycin‐resistant *Enterococcus faecium* in Australasia and is associated with heterogeneous and novel mutations. Frontiers in Microbiology, 12, 749935. Available from: 10.3389/FMICB.2021.749935 34745054 PMC8564391

[mmi15128-bib-0040] Ling, L.L. , Schneider, T. , Peoples, A.J. , Spoering, A.L. , Engels, I. , Conlon, B.P. et al. (2015) A new antibiotic kills pathogens without detectable resistance. Nature, 517(7535), 455–459. Available from: 10.1038/nature14098 25561178 PMC7414797

[mmi15128-bib-0041] Liu, J. , Gefen, O. , Ronin, I. , Bar‐Meir, M. & Balaban, N.Q. (2020) Effect of tolerance on the evolution of antibiotic resistance under drug combinations. Science, 367(6474), 200–204. Available from: 10.1126/science.aay3041 31919223

[mmi15128-bib-0042] Mason, K.L. , Stepien, T.A. , Blum, J.E. , Holt, J.F. , Labbe, N.H. , Rush, J.S. et al. (2011) From commensal to pathogen: translocation of *Enterococcus faecalis* from the midgut to the hemocoel of manduca sexta. mBio, 2(3), e00065–e00011. Available from: 10.1128/MBIO.00065-11/-/DCSUPPLEMENTAL/MBIO.00065-11-SF02.EPS 21586646 PMC3101781

[mmi15128-bib-0043] Matsumoto, Y. , Yasukawa, J. , Ishii, M. , Hayashi, Y. , Miyazaki, S. & Sekimizu, K. (2016) A critical role of mevalonate for peptidoglycan synthesis in *Staphylococcus aureus* . Scientific Reports, 6(1), 1–14. Available from: 10.1038/srep22894 26961421 PMC4790635

[mmi15128-bib-0044] Moellering, R. C. (1992) ‘Emergence of *Enterococcus* as a significant pathogen.’, Clinical Infectious Diseases, 14(6), pp. 1173–6. http://www.ncbi.nlm.nih.gov/pubmed/1623072 (Accessed 5 May 2017). 1623072 10.1093/clinids/14.6.1173

[mmi15128-bib-0045] Moon, T.M. , D'Andréa, É.D. , Lee, C.W. , Soares, A. , Jakoncic, J. , Desbonnet, C. et al. (2018) The structures of penicillin‐binding protein 4 (PBP4) and PBP5 from enterococci provide structural insights into β‐lactam resistance. The Journal of Biological Chemistry, 293(48), 18574–18585. Available from: 10.1074/JBC.RA118.006052 30355734 PMC6290140

[mmi15128-bib-0046] Moreno‐Letelier, A. , Olmedo, G. , Eguiarte, L.E. , Martinez‐Castilla, L. & Souza, V. (2011) Parallel evolution and horizontal gene transfer of the *pst* operon in firmicutes from oligotrophic environments. International Journal of Evolutionary Biology, 2011, 781642. Available from: 10.4061/2011/781642 21461370 PMC3065170

[mmi15128-bib-0047] Muller, C. , Massier, S. , le Breton, Y. & Rincé, A. (2018) The role of the CroR response regulator in resistance of *Enterococcus faecalis* to D‐cycloserine is defined using an inducible receiver domain. Molecular Microbiology, 107(3), 416–427. Available from: 10.1111/mmi.13891 29205552

[mmi15128-bib-0048] Novak, R. , Cauwels, A. , Charpentier, E. & Tuomanen, E. (1999) Identification of a *Streptococcus pneumoniae* gene locus encoding proteins of an ABC phosphate transporter and a two‐component regulatory system. Journal of Bacteriology, 181(4), 1126–1133. Available from: 10.1128/JB.181.4.1126-1133.1999/ASSET/E735B237-FF22-40D7-B0F5-09EF5ED9DF9C/ASSETS/GRAPHIC/JB0491194004.JPEG 9973337 PMC93488

[mmi15128-bib-0049] Palmer, K.L. , Godfrey, P. , Griggs, A. , Kos, V.N. , Zucker, J. , Desjardins, C. et al. (2012) Comparative genomics of enterococci: variation in *Enterococcus faecalis*, clade structure in *E. faecium*, and defining characteristics of *E*. *gallinarum* and *E. casseliflavus*’. mBio, 3(1), 1–11. Available from: 10.1128/MBIO.00318-11 PMC3374389 22354958

[mmi15128-bib-0050] Piepenbreier, H. , Diehl, A. & Fritz, G. (2019) Minimal exposure of lipid II Cycle intermediates triggers cell wall antibiotic resistance. Nature Communications, 10(1), 1–13. Available from: 10.1038/s41467-019-10673-4 PMC6588590 31227716

[mmi15128-bib-0051] Poyart, C. & Trieu‐Cuot, P. (1997) A broad‐host‐range mobilizable shuttle vector for the construction of transcriptional fusions to beta‐galactosidase in gram‐positive bacteria. FEMS Microbiology Letters, 156(2), 193–198. Available from: 10.1111/J.1574-6968.1997.TB12726.X 9513264

[mmi15128-bib-0052] Rigottier‐Gois, L. , Madec, C. , Navickas, A. , Matos, R.C. , Akary‐Lepage, E. , Mistou, M.Y. et al. (2015) The surface rhamnopolysaccharide Epa of *Enterococcus faecalis* is a key determinant of intestinal colonization. The Journal of Infectious Diseases, 211(1), 62–71. Available from: 10.1093/INFDIS/JIU402 25035517

[mmi15128-bib-0053] Santi, I. , Manfredi, P. , Maffei, E. , Egli, A. & Jenal, U. (2021) Evolution of antibiotic tolerance shapes resistance development in chronic *Pseudomonas aeruginosa* infections. MBio, 12(1), 1–17. Available from: 10.1128/MBIO.03482-20/SUPPL_FILE/MBIO.03482-20-ST004.DOCX PMC7885114 33563834

[mmi15128-bib-0054] Shukla, R. , Medeiros‐Silva, J. , Parmar, A. , Vermeulen, B.J.A. , Das, S. , Paioni, A.L. et al. (2020) Mode of action of teixobactins in cellular membranes. Nature Communications, 11(1), 2848. Available from: 10.1038/s41467-020-16600-2 PMC7275090 32503964

[mmi15128-bib-0055] Shukla, R. , Lavore, F. , Maity, S. , Derks, M.G.N. , Jones, C.R. , Vermeulen, B.J.A. et al. (2022) Teixobactin kills bacteria by a two‐pronged attack on the cell envelope. Nature, 608, 390–396. Available from: 10.1038/s41586-022-05019-y 35922513 PMC9365693

[mmi15128-bib-0056] Singh, K.V. & Murray, B.E. (2019) Loss of a major enterococcal polysaccharide antigen (Epa) by *Enterococcus faecalis* is associated with increased resistance to ceftriaxone and carbapenems. Antimicrobial Agents and Chemotherapy, 63(5), e00481–e00419. Available from: 10.1128/AAC.00481-19 30858216 PMC6496094

[mmi15128-bib-0057] Smith, R.E. , Salamaga, B. , Szkuta, P. , Hajdamowicz, N. , Prajsnar, T.K. , Bulmer, G.S. et al. (2019) Decoration of the enterococcal polysaccharide antigen EPA is essential for virulence, cell surface charge and interaction with effectors of the innate immune system. PLoS Pathogens, 15(5), e1007730. Available from: 10.1371/JOURNAL.PPAT.1007730 31048927 PMC6497286

[mmi15128-bib-0058] Solheim, M. , la Rosa, S.L. , Mathisen, T. , Snipen, L.G. , Nes, I.F. & Brede, D.A. (2014) Transcriptomic and functional analysis of NaCl‐induced stress in *Enterococcus faecalis* . PLoS One, 9, 1–13. Available from: 10.1371/journal.pone.0094571 PMC3995695 24755907

[mmi15128-bib-0059] Takada, H. , Shiwa, Y. , Takino, Y. , Osaka, N. , Ueda, S. , Watanabe, S. et al. (2018) Essentiality of *walRK* for growth in *Bacillus subtilis* and its role during heat stress. Microbiology (United Kingdom), 164(4), 670–684. Available from: 10.1099/MIC.0.000625/CITE/REFWORKS 29465029

[mmi15128-bib-0060] Timmler, S.B. , Kellogg, S.L. , Atkinson, S.N. , Little, J.L. , Djorić, D. & Kristich, C.J. (2022) CroR regulates expression of *pbp4(5)* to promote cephalosporin resistance in *Enterococcus faecalis* . mBio, 13(4), e0111922. Available from: 10.1128/MBIO.01119-22 35913163 PMC9426447

[mmi15128-bib-0061] Tomasz, A. , Albino, A. & Zanati, E. (1970) Multiple antibiotic resistance in bacterium with supressed autolytic system. Nature, 227, 138–140. 4393335 10.1038/227138a0

[mmi15128-bib-0062] Van Tyne, D. & Gilmore, M.S. (2014) Friend turned foe: evolution of enterococcal virulence and antibiotic resistance. Annual Review of Microbiology, 68, 337–356. Available from: 10.1146/annurev-micro-091213-113003 PMC4384341 25002090

[mmi15128-bib-0063] Varadi, M. , Anyango, S. , Deshpande, M. , Nair, S. , Natassia, C. , Yordanova, G. et al. (2021) AlphaFold protein structure database: massively expanding the structural coverage of protein‐sequence space with high‐accuracy models. Nucleic Acids Research, 50, D439–D444. Available from: 10.1093/nar/gkab1061 PMC8728224 34791371

[mmi15128-bib-0064] Wadhawan, A. , Simoes da Silva, C.J. , Nunes, C.D. , Edwards, A.M. & Dionne, M.S. *E. faecalis* acquires resistance to antimicrobials and insect immunity via common mechanisms. 10.1101/2022.08.17.504265 bioRxiv Preprint.

[mmi15128-bib-0065] Windels, E.M. , Van Den Bergh, B. & Michiels, J. (2020) Bacteria under antibiotic attack: different strategies for evolutionary adaptation. PLoS Pathogens, 16(5), 1–8. Available from: 10.1371/journal.ppat.1008431 PMC7205213 32379814

