# Supplementary material for: The two‐component system CroRS acts as a master regulator of cell envelope homeostasis to confer antimicrobial tolerance in the bacterial pathogen Enterococcus faecalis
Source: Mol Microbiol. 2023 Jul 20;120(3):408–24. doi: 10.1111/mmi.15128 (PMC10952268; doi:10.1111/mmi.15128)
Supplement: Supplementary file 1 — Appendix S1. [file MMI-120-408-s001.pdf]

Supplementary Tables

Contents:

Contents:	Page No.
**Table S1. Genes differentially expressed in *E. faecalis* wild-type versus Δ*croRS* in the absence of TXB.**	2 - 25
**Table S2. Genes differentially expressed in *E. faecalis* wild-type versus Δ*croRS* in the presence of TXB.**	26 - 44
**Table S3. Genes differentially expressed in *E. faecalis* wild-type in the presence versus absence of TXB.**	45 - 75
**Table S4. Genes differentially expressed in *E. faecalis* Δ*croRS* in the presence versus absence of TXB.**	76 - 92
**Table S5. The TXB-induced CroRS regulon in *E. faecalis* wild-type.**†	93 - 98
**Table S6. A comparison of RNAseq and qRT-PCR differential gene expression in the *E. faecalis* WT and Δ*croRS* strains in the presence and absence of TXB.**	99
**Table S7. Bacterial strains, plasmids and primers used in this study.**	100 - 102
**Figure S1. Optimisation of teixobactin concentration for RNA sequencing.**	103
**Figure S2. Determing the TXB-induced CroRS regulon.**	104
**Figure S3. Differential gene expression in the *E. faecalis* WT and Δ*croRS* in the presence and absence of teixobactin.**	105
**Figure S4. Growth curve of the evolved Δ*croRS* mutants 1BS – 5BS alongside the isogenic Δ*croRS* mutant and WT (JH2-2).**	106
**Figure S5. A 3D model and amino acid sequence comparison of the putative heptaprenyl diphosphate synthase identifying mutations in the gain of tolerance Δ*croRS* mutants.**	107
**Figure S6. Growth curve of the *E. faecalis* wild-type, Δ*croRS*, 4BS and Δ*croRS*_Δ*hppS* strains.**	108

Table S1. Genes differentially expressed in *E. faecalis* wild-type versus Δ*croRS* in the absence of TXB.

JH2-2 Locus Tag	V583 Gene	Gene Name	F/C (log_2)	Gene Function	P_{adj}
**Amino acid metabolism**					
O994_RS13680	EF0097	*pfoR*	3.0	regulatory protein	2.40E-29
O994_RS13675	EF0098	*sdhB-1*	2.8	L-serine dehydratase, iron-sulfur-dependent subunit β	5.20E-21
O994_RS13670	EF0099	*sdhA-1*	2.6	iron-sulfur-dependent L-serine dehydratase subunit α	2.20E-16
O994_RS13645	EF0104	*arcA*	9.3	arginine deiminase	4.60E-193
O994_RS13640	EF0105	*argF-1*	9.0	ornithine carbamoyltransferase	1.20E-152
O994_RS13600	EF0115		2.0	endoribonuclease L-PSP	2.10E-04
O994_RS02700	EF0784	*metK*	1.4	S-adenosylmethionine synthetase	8.80E-02
O994_RS06735	EF1584	*cysK*	1.9	cysteine synthase A	4.00E-06
O994_RS09975	EF2421	*thrC*	1.8	threonine synthase	3.40E-03
O994_RS09980	EF2422	*hom*	1.7	homoserine dehydrogenase	2.40E-02
O994_RS09990	EF2424		1.6	pyrroline-5-carboxylate reductase	1.30E-02
O994_RS10505	EF2568		2.8	class V aminotransferase	1.10E-09
O994_RS01470	EF2983		1.8	glutamyl-tRNA amidotransferase subunit A	2.00E-03
O994_RS00525	EF3178	*dapE*	2.5	succinyl-diaminopimelate desuccinylase	8.60E-13
O994_RS14060	EF0014	*purA*	-4.2	adenylosuccinate synthetase	8.90E-47
O994_RS13940	EF0038	*proB*	-2.9	γ-glutamyl kinase	2.20E-06
O994_RS12655	EF0368		-2.0	aspartate kinase	3.30E-03
O994_RS01925	EF0634		-4.3	tyrosine decarboxylase	1.10E-48
O994_RS02475	EF0737		-1.8	amidase	5.50E-03
O994_RS06620	EF1561	*aroE*	-2.0	shikimate 5-dehydrogenase	8.40E-06
O994_RS07365	EF1719	*pyrB*	-1.7	aspartate carbamoyltransferase catalytic subunit	7.30E-02
O994_RS07420	EF1731	*aroD*	-2.3	3-dehydroquinase	7.80E-09
O994_RS09560	EF2361	*purB*	-1.8	adenylosuccinate lyase	1.10E-04
**Autolysis**					
O994_RS05530	EF1348		1.7	glucan 1,6-α-glucosidase	1.30E-03
O994_RS11610	EF2863		2.3	endo-β-*N*-acetylglucosaminidase	1.70E-09

O994_RS06730	EF1583		-1.6	*N*-acetylmuramoyl-L-alanine amidase	2.40E-02
**Carbohydrate metabolism**					
O994_RS13795	EF0069		2.5	*N*-acetylmannosamine-6-phosphate 2-epimerase	6.00E-10
O994_RS13385	EF0174	*deoC*	2.1	2-deoxyribose-5-phosphate aldolase	2.00E-06
O994_RS13345	EF0185	*deoB*	1.8	phosphopentomutase	7.60E-04
O994_RS13025	EF0253		3.3	aldehyde dehydrogenase	3.50E-18
O994_RS12860	EF0271	*arb*	2.0	6-phospho-β-glucosidase	1.10E-04
O994_RS12440	EF0413	*mtlD*	5.2	mannitol-1-phosphate 5-dehydrogenase	6.50E-15
O994_RS09760	EF0423	*eda-1*	3.0	keto-hydroxyglutarate-aldolase/keto-deoxy-phosphogluconate aldolase	8.30E-10
O994_RS02145	EF0677		2.6	phosphoglucomutase/phosphomannomutase	1.00E-14
O994_RS02625	EF0769		1.5	PEP phosphonomutase	7.90E-02
O994_RS03215	EF0900	*adhE*	1.5	bifunctional acetaldehyde-CoA/alcohol dehydrogenase	3.10E-02
O994_RS03485	EF0955		1.4	aldose 1-epimerase	3.70E-02
O994_RS03490	EF0956	*pgmB*	1.7	β-phosphoglucomutase	6.50E-05
O994_RS03495	EF0957		2.0	maltose phosphorylase	9.60E-08
O994_RS03805	EF1020		2.0	glycosyl hydrolase	2.80E-05
O994_RS03830	EF1024	*ppdK*	2.3	pyruvate phosphate dikinase	7.80E-05
O994_RS04145	EF1068	*galM*	4.7	aldose 1-epimerase	5.80E-55
O994_RS04150	EF1069	*galK*	3.6	galactokinase	1.10E-20
O994_RS04155	EF1070	*galE-1*	3.5	UDP-glucose 4-epimerase	2.60E-19
O994_RS04160	EF1071	*galT*	2.4	galactose-1-phosphate uridylyltransferase	2.10E-08
O994_RS04330	EF1108	*lutA*	2.6	oxidoreductase	6.50E-16
O994_RS04340	EF1110		2.1	lactate utilisation protein C	1.90E-08
O994_RS04475	EF1138		1.6	aldo/keto reductase family oxidoreductase	1.20E-02
O994_RS09520	EF1213	*alsS*	1.9	acetolactate synthase	6.00E-03
O994_RS05535	EF1349		1.7	glycosyl hydrolase	2.40E-03
O994_RS05555	EF1354	*pdhB*	1.5	pyruvate dehydrogenase complex, E1 component subunit β	9.10E-02
O994_RS05560	EF1355	*aceF*	1.8	adihydrolipoamide acetyltransferase	1.00E-03
O994_RS05565	EF1356	*lpdA*	1.6	dihydrolipoamide dehydrogenase	3.20E-03
O994_RS05575	EF1358	*gldA*	3.5	glycerol dehydrogenase	5.20E-28

O994_RS05740	EF1390	*fdhA*	1.9	NAD-dependent formate dehydrogenase subunit α	1.10E-02
O994_RS06455	EF1526	*gap-1*	2.4	glyceraldehyde 3-phosphate dehydrogenase	7.60E-09
O994_RS06810	EF1602		4.9	glycosyl hydrolase	1.90E-68
O994_RS06860	EF1612	*pflA*	1.3	pyruvate formate-lyase activating enzyme	8.80E-02
O994_RS06865	EF1613	*pflB*	1.8	formate acetyltransferase	2.30E-06
O994_RS06945	EF1630	*eutA*	1.8	ethanolamine utilisation protein	4.50E-03
O994_RS06965	EF1635	*pduQ*	2.2	propanol dehydrogenase	6.00E-05
O994_RS06975	EF1638	*pduV*	1.6	propanediol utilization protein	3.40E-02
O994_RS07090	EF1661	*bkdD*	4.5	branched-chain α-keto acid dehydrogenase	4.20E-14
O994_RS07095	EF1663	*ptb*	6.3	branched-chain phosphotransacylase	5.30E-13
O994_RS07115	EF1667		2.2	short chain dehydrogenase	2.00E-06
O994_RS07780	EF1805		3.1	glycosyl hydrolase	1.50E-09
O994_RS07785	EF1806		1.7	hexose kinase	2.20E-02
O994_RS01710	EF1807	*lacD-2*	2.7	tagatose 1,6-diphosphate aldolase	3.90E-07
O994_RS01700	EF1834	*lacB*	3.5	galactose-6-phosphate isomerase subunit	2.40E-24
O994_RS01695	EF1835	*lacA*	3.7	galactose-6-phosphate isomerase subunit	2.90E-31
O994_RS08210	EF1918	*pglA*	2.6	hypothetical protein	3.10E-18
O994_RS08345	EF1950		4.0	glucosamine-fructose-6-phosphate aminotransferase	5.10E-19
O994_RS08350	EF1951		4.7	phosphosugar-binding protein	6.70E-26
O994_RS08965	EF2073	*prsA-1*	1.5	ribose-phosphate pyrophosphokinase	5.60E-02
O994_RS09890	EF2401	*acyP*	2.0	acylphosphatase	1.30E-04
O994_RS09995	EF2425		1.6	phosphoglucomutase/phosphomannomutase	2.30E-03
O994_RS10460	EF2559		1.7	pyruvate flavodoxin/ferredoxin oxidoreductase family protein	3.00E-03
O994_RS12005	EF2961	*rbsK*	1.5	ribokinase	6.30E-02
O994_RS00695	EF3140		3.2	iron-containing alcohol dehydrogenase	2.40E-21
O994_RS00690	EF3141		3.7	D-isomer specific 2-hydroxyacid dehydrogenase family protein	5.70E-29
O994_RS00685	EF3142	*gnd*	4.2	6-phosphogluconate dehydrogenase	4.40E-37
O994_RS00300	EF3235		1.8	gluconate kinase	3.40E-05
O994_RS14240	EF3310		6.6	sorbitol-6-phosphate 2-dehydrogenase	2.70E-38
O994_RS14210	EF3316	*maeE*	1.7	malic enzyme family protein	2.30E-03
O994_RS14205	EF3317	*citM*	2.9	oxaloacetate decarboxylase	7.60E-14

O994_RS14170	EF3324		4.3	sodium ion-translocating decarboxylase subunit β	9.20E-46
O994_RS14165	EF3325		4.8	acetyl-CoA carboxylase biotin carboxyl carrier protein subunit	6.10E-53
O994_RS13850	EF0059	*glmU*	-1.6	UDP-*N*-acetylglucosamine pyrophosphorylase	1.00E-02
O994_RS03195	EF0895		-1.8	glycerol dehydrogenase	5.30E-02
O994_RS03460	EF0949	*eutD*	-1.9	phosphotransacetylase	2.10E-05
O994_RS06885	EF1617	*eutQ*	-3.2	hypothetical protein	1.40E-08
O994_RS06890	EF1618	*eutH*	-3.6	ethanolamine utilization protein	1.50E-17
O994_RS06905	EF1621		-3.5	phosphate propanoyltransferase	2.30E-11
O994_RS00600	EF3163	*prsA-2*	-3.6	ribose-phosphate pyrophosphokinase	2.30E-27
**Cellular community**					
O994_RS00880	EF3106	*opp2A*	2.9	oligopeptide ABC transporter substrate-binding protein	2.10E-19
O994_RS00875	EF3107	*opp2C*	2.8	oligopeptide ABC transporter permease	4.10E-15
**Energy metabolism**					
O994_RS10470	EF2561		2.4	ferredoxin-NADP(+) reductase subunit α	8.80E-09
O994_RS01165	EF3037	*pepA*	3.0	glutamyl-aminopeptidase	1.10E-17
O994_RS10465	EF2560	*gltA*	2.1	oxidoreductase	5.90E-06
O994_RS10685	EF2607	*atpC*	1.5	F_0F_1 ATP synthase subunit ε	1.50E-02
O994_RS10690	EF2608	*atpD*	1.8	F_0F_1 ATP synthase subunit β	2.60E-03
O994_RS06270	EF1492		-2.9	V-type ATPase subunit F	3.40E-22
O994_RS06275	EF1493		-2.2	V-type ATP synthase subunit I	5.00E-11
O994_RS06280	EF1494		-1.5	V-type ATP synthase subunit K	2.10E-02
O994_RS07325	EF1711		-1.8	carbonic anhydrase	5.70E-03
O994_RS00200	EF3257		-2.1	pyridine nucleotide-disulfide family oxidoreductase	3.20E-05
**Folding, sorting and degradation**					
O994_RS07025	EF1646	*hslU*	-2.6	heat shock protein	1.00E-11
O994_RS12640	EF0371		-3.3	class V aminotransferase	3.20E-28
O994_RS02950	EF0846		-1.7	DEAD/DEAH box helicase	6.30E-04
O994_RS04635	EF1170	*rho*	-2.1	transcription termination factor	4.20E-07
O994_RS08950	EF2070	*mnmA*	-2.7	tRNA-specific 2-thiouridylase	1.50E-16

O994_RS09650	EF2380	*rseP*	-1.5	membrane-associated zinc metalloprotease	2.50E-02
O994_RS10750	EF2620	*secG*	-2.8	preprotein translocase subunit	4.00E-11
O994_RS11250	EF2730	*secE*	-3.8	preprotein translocase subunit	2.60E-14
O994_RS11970	EF2934	*thiI*	-2.8	thiazole biosynthesis protein	4.20E-11
O994_RS01035	EF3064	*pnpA*	-2.7	polynucleotide phosphorylase/polyadenylase	3.10E-13
**Glycan biosynthesis and metabolism**					
O994_RS13605	EF0114		1.6	glycosyl hydrolase	5.20E-03
O994_RS06105	EF1460		2.2	LysM domain-containing protein	2.30E-05
O994_RS07305	EF1707		2.2	glycosyl hydrolase	2.00E-06
O994_RS10235	EF2476	*pbp4(5)*	1.7	penicillin-binding protein 4	2.10E-04
O994_RS10850	EF2644		1.7	diacylglycerol kinase catalytic subunit	3.60E-04
O994_RS02100	EF0668	*murE*	-2.0	UDP-*N*-acetylmuramoylalanyl-D-glutamate--L-lysine ligase	1.60E-05
O994_RS02105	EF0669		-2.6	polysaccharide biosynthesis family protein	1.20E-11
O994_RS04630	EF1169	*murAB*	-2.5	UDP-*N*-acetylglucosamine 1-carboxyvinyltransferase	1.10E-09
O994_RS04645	EF1172		-4.2	teichoic acid biosynthesis protein B	5.30E-17
O994_RS08165	EF1908	*murC*	-2.9	UDP-*N*-acetylmuramate-L-alanine ligase	5.70E-18
O994_RS08320	EF1945		-2.3	putative polyglycerophosphate lipoteichoic acids	4.10E-06
O994_RS09160	EF2172	*ispD*	-2.2	2-C-methyl-D-erythritol 4-phosphate cytidylyltransferase	6.30E-07
O994_RS09175	EF2175		-2.2	licD-like protein	6.60E-06
O994_RS09275	EF2198	*tagO*	-2.0	glycosyl transferase family protein	3.70E-06
O994_RS10675	EF2605	*murAA*	-2.8	UDP-*N*-acetylglucosamine 1-carboxyvinyltransferase 1	1.20E-19
O994_RS10920	EF2658		-2.6	FemAB family protein	5.70E-14
O994_RS11345	EF2750	*dltX*	-1.6	teichoic acid D-ala incorporation-associated protein	5.00E-02
O994_RS01055	EF3060		-2.2	peptidoglycan DL-endopeptidase	1.10E-07
**Lipid Metabolism**					
O994_RS04675	EF1359		1.8	hypothetical protein	2.30E-05
O994_RS05580	EF1359	*dhaM*	3.3	hypothetical protein	3.00E-22
O994_RS05585	EF1360	*dhaK*	3.1	dihydroxyacetone kinase	5.90E-19
O994_RS05590	EF1361	*dhaL*	2.7	dihydroxyacetone kinase	3.60E-12
O994_RS08255	EF1928	*glpO*	2.9	α-glycerophosphate oxidase	4.40E-18

O994_RS08260	EF1929	*glpK*	3.2	glycerol kinase	5.00E-18
O994_RS01370	EF3005		2.7	choloylglycine hydrolase	4.10E-15
O994_RS06665	EF1570		-2.2	DegV family protein	9.40E-09
O994_RS06995	EF1643	*plsY*	-2.9	hypothetical protein	1.90E-09
O994_RS08150	EF1904		-1.7	glycerophosphoryl diester phosphodiesterase family protein	7.30E-02
O994_RS09115	EF2163		-2.1	glycerophosphoryl diester phosphodiesterase	5.10E-03
O994_RS11070	EF2691	*plsC*	-1.6	1-acyl-sn-glycerol-3-phosphate acyltransferase	6.20E-02
O994_RS11705	EF2883	*fabK*	-1.8	enoyl-ACP reductase	6.10E-05
O994_RS11715	EF2885	*fabH*	-1.8	3-oxoacyl-ACP synthase III	1.60E-05
**Membrane transport**					
O994_RS14035	EF0019	*mptA*	1.4	PTS system transporter subunit IIB	4.60E-02
O994_RS14030	EF0020	*mptAB*	2.1	PTS system mannose-specific transporter subunit IIAB	5.70E-11
O994_RS14025	EF0021	*mptC*	2.1	PTS system mannose-specific transporter subunit IIC	8.60E-12
O994_RS14020	EF0022	*mptD*	1.8	PTS system mannose-specific transporter subunit IID	2.40E-07
O994_RS13625	EF0108	*arcD*	6.5	C4-dicarboxylate transporter	1.20E-26
O994_RS13375	EF0176		2.0	hypothetical protein	3.20E-04
O994_RS13370	EF0177		1.9	hypothetical protein	1.10E-06
O994_RS13365	EF0178		1.7	ABC transporter ATP-binding protein	4.60E-04
O994_RS13360	EF0179		1.6	ABC transporter permease	7.20E-03
O994_RS12865	EF0270		2.1	PTS system β-glucoside-specific transporter subunit IIABC	1.10E-07
O994_RS12575	EF0385		3.5	major facilitator family transporter	2.60E-15
O994_RS12525	EF0396		1.5	AI-2E family transporter	4.90E-02
O994_RS12450	EF0411		6.5	PTS system mannitol-specific transporter subunit IIBC	1.20E-26
O994_RS12445	EF0412	*mltF*	5.5	PTS system mannitol-specific transporter subunit IIA	6.70E-17
O994_RS02205	EF0688		1.7	ABC transporter ATP-binding protein	1.10E-03
O994_RS02230	EF0693	*fruK-1*	1.7	1-phosphofructokinase	4.50E-02
O994_RS02235	EF0694		2.3	PTS system fructose-specific transporter subunit IIBC	7.80E-04
O994_RS03040	EF0862		1.7	glycine betaine/carnitine/choline ABC transporter permease	5.10E-04
O994_RS03045	EF0863		2.0	glycine betaine/carnitine/choline ABC transporter	1.20E-05
O994_RS03050	EF0864		2.1	glycine betaine/carnitine/choline ABC transporter permease	3.40E-06

O994_RS03055	EF0865		1.6	glycine betaine/carnitine/choline transporter ATP-binding protein	1.10E-02
O994_RS03075	EF0871		1.4	cation transporter E1-E2 family ATPase	6.80E-02
O994_RS03095	EF0875		2.0	copper-translocating P-type ATPase	3.30E-06
O994_RS03275	EF0912		1.4	peptide ABC transporter ATP-binding protein	6.70E-02
O994_RS03405	EF0938	*ugpC*	2.6	sugar ABC transporter ATP-binding protein	4.10E-21
O994_RS03500	EF0958		1.7	PTS system transporter subunit IIABC	2.00E-05
O994_RS03765	EF1012		2.1	PTS system transporter subunit IIB	1.10E-03
O994_RS03770	EF1013		2.1	PTS system transporter subunit IIC	1.70E-05
O994_RS03790	EF1017		1.9	PTS system transporter subunit IIB	3.40E-07
O994_RS03795	EF1018		2.0	PTS system transporter subunit IIA	5.30E-08
O994_RS01715	EF1018		2.5	PTS system transporter subunit IIA	3.20E-05
O994_RS03800	EF1019		2.0	PTS system transporter subunit IIC	2.00E-07
O994_RS04585	EF1160		3.3	PTS system cellobiose-specific transporter subunit IIC	5.50E-11
O994_RS04940	EF1232		2.2	ABC transporter permease	2.40E-05
O994_RS04945	EF1233		1.8	ABC transporter permease	1.70E-02
O994_RS04950	EF1234		2.0	ABC transporter substrate-binding protein	1.30E-04
O994_RS05420	EF1320		2.0	ABC transporter ATP-binding protein	2.00E-06
O994_RS05425	EF1321		1.7	permease domain-containing protein	2.50E-04
O994_RS06470	EF1529		2.1	PTS system transporter subunit IIC	3.90E-04
O994_RS06805	EF1601		4.3	PTS system transporter subunit IIABC	7.40E-36
O994_RS07765	EF1802		2.1	PTS system transporter subunit IID	4.30E-03
O994_RS07770	EF1803		2.6	PTS system transporter subunit IIC	1.10E-06
O994_RS07775	EF1804		2.5	PTS system transporter subunit IIB	8.70E-05
O994_RS09755	EF1836		2.3	PTS system transporter subunit IIA	1.70E-04
O994_RS09745	EF1838		1.7	PTS system transporter subunit IIC	2.90E-02
O994_RS08220	EF1920		4.0	C4-dicarboxylate anaerobic carrier	7.00E-23
O994_RS08250	EF1927	*glpF*	2.4	glycerol uptake facilitator protein	9.70E-08
O994_RS08355	EF1952		5.5	PTS system transporter subunit IID	2.00E-21
O994_RS08360	EF1953		5.5	PTS system transporter subunit IIC	8.50E-34
O994_RS09350	EF2213		2.7	PTS system transporter subunit IIBC	1.40E-16
O994_RS09395	EF2222		2.0	ABC transporter permease	1.00E-03

O994_RS09400	EF2223		2.3	ABC transporter permease	3.70E-08
O994_RS10665	EF2603		1.4	PTS system transporter subunit IIA	9.90E-02
O994_RS10865	EF2647	*grtP*	2.2	gluconate-proton symporter	6.40E-05
O994_RS12020	EF2964	*ulaA*	3.1	PTS system ascorbate-specific transporter subunit IIC	2.60E-13
O994_RS12025	EF2965		3.3	hypothetical protein	2.50E-19
O994_RS09765	EF2986		3.6	ABC transporter ATP-binding protein	5.10E-24
O994_RS01360	EF3007		1.7	chloride ion channel protein	1.20E-03
O994_RS01205	EF3029		1.9	PTS system transporter subunit IID	2.00E-03
O994_RS01200	EF3030		2.1	PTS system transporter subunit IIC	1.10E-05
O994_RS01195	EF3031		2.1	PTS system transporter subunit IIB	2.80E-04
O994_RS01185	EF3033		2.0	PTS system transporter subunit IIA	9.50E-03
O994_RS00870	EF3108	*opp2B*	2.2	oligopeptide ABC transporter permease	1.00E-09
O994_RS00865	EF3109	*opp2F*	1.4	oligopeptide ABC transporter ATP-binding protein	8.60E-02
O994_RS00715	EF3136		2.1	PTS system transporter subunit IIA	3.10E-03
O994_RS00710	EF3137		2.3	PTS system transporter subunit IIB	6.30E-05
O994_RS00705	EF3138		2.7	PTS system transporter subunit IID	6.90E-08
O994_RS00700	EF3139		3.0	PTS system transporter subunit IIC	8.00E-14
O994_RS00375	EF3210		4.9	PTS system transporter subunit IIA	2.40E-60
O994_RS00370	EF3211		5.1	PTS system transporter subunit IIB	1.70E-52
O994_RS00365	EF3212		4.9	PTS system transporter subunit IIC	7.70E-49
O994_RS00360	EF3213		4.2	PTS system transporter subunit IID	8.60E-39
O994_RS14260	EF3306		3.6	PTS system sorbitol-specific transporter subunit IIBC	2.00E-13
O994_RS14255	EF3307	*srlA*	3.5	PTS system sorbitol-specific transporter subunit IIC	8.60E-11
O994_RS14155	EF3327		4.9	citrate transporter	4.30E-83
O994_RS14045	EF0017		-2.7	ABC-F family ATPase	1.70E-19
O994_RS13695	EF0094		-2.3	formate/nitrite transporter family protein	5.30E-05
O994_RS13100	EF0238	*cbiO*	-1.5	cobalt transporter ATP-binding protein	5.90E-02
O994_RS01930	EF0635		-4.3	amino acid permease	1.20E-41
O994_RS01935	EF0636	*nhaC*	-3.7	Na^+/H^+ antiporter	2.40E-27
O994_RS02260	EF0699		-1.6	AI-2E family transporter	1.50E-02

O994_RS02395	EF0720		-2.8	voltage-gated chloride channel family protein	3.00E-11
O994_RS02485	EF0739	*pnuC*	-3.5	nicotinamide mononucleotide transporter	1.40E-25
O994_RS02510	EF0744		-1.7	sodium/dicarboxylate symporter family protein	1.60E-03
O994_RS02575	EF0758		-2.4	cadmium-translocating P-type ATPase	8.00E-07
O994_RS02585	EF0760		-1.7	amino acid ABC transporter ATP-binding protein	5.50E-02
O994_RS02590	EF0761		-3.2	amino acid ABC transporter amino acid-binding/permease	2.50E-20
O994_RS02720	EF0789		-2.3	ABC transporter ATP-binding protein	1.90E-04
O994_RS03030	EF0860		-2.6	APC family permease	1.30E-10
O994_RS03085	EF0872		-2.2	potassium uptake protein	4.80E-08
O994_RS03355	EF0928		-3.8	glucose uptake protein	4.60E-19
O994_RS03360	EF0929		-5.0	amino acid permease	3.40E-24
O994_RS04190	EF1078	*emeA*	-2.5	multidrug efflux MFS transporter	3.50E-09
O994_RS04465	EF1135	*mscS*	-1.5	mechanosensitive ion channel protein	7.70E-02
O994_RS05495	EF1341	*macA*	-1.8	ABC transporter ATP-binding protein/permease	1.20E-02
O994_RS05545	EF1352	*mgtA*	-1.4	magnesium-translocating P-type ATPase	6.60E-02
O994_RS05790	EF1400		-2.8	cadmium-translocating P-type ATPase	1.20E-14
O994_RS06425	EF1519		-2.7	cation transporter E1-E2 family ATPase	2.10E-16
O994_RS06525	EF1541		-1.5	riboflavin transporter	9.00E-02
O994_RS06685	EF1574		-1.6	Na^+/H^+ antiporter	5.20E-03
O994_RS06835	EF1607		-1.6	PTS transporter subunit EIIC	2.80E-02
O994_RS07050	EF1654	*ylqF*	-3.2	GTPase	4.10E-13
O994_RS07370	EF1720		-2.4	uracil permease	2.00E-04
O994_RS07430	EF1733		-2.8	ABC transporter ATP-binding protein/peptidase	1.30E-13
O994_RS07565	EF1758	*pstC*	-1.6	phosphate ABC transporter permease	1.30E-02
O994_RS07570	EF1759		-1.6	phosphate ABC transporter substrate-binding protein	9.50E-03
O994_RS07970	EF1867		-1.9	ABC transporter permease	9.50E-03
O994_RS08840	EF2047		-4.3	amino acid permease	6.80E-30
O994_RS09065	EF2153		-2.0	ABC transporter ATP-binding protein	2.20E-05
O994_RS09215	EF2183		-2.2	ABC transporter permease	3.30E-05
O994_RS10175	EF2460	*typA*	-3.6	GTP-binding protein	2.40E-33
O994_RS10340	EF2498	*metN-2*	-2.1	ABC transporter ATP-binding protein	1.80E-05

O994_RS10880	EF2650		-1.6	spermidine/putrescine ABC transporter permease	5.30E-02
O994_RS10885	EF2651		-1.9	spermidine/putrescine ABC transporter permease	5.70E-03
O994_RS10890	EF2652		-2.2	spermidine/putrescine ABC transporter ATP-binding protein	7.80E-07
O994_RS10925	EF2659		-1.8	QueT transporter family protein	2.80E-03
O994_RS11655	EF2873	*yqeH*	-2.2	GTP-binding protein	4.40E-08
O994_RS11975	EF2935		-2.5	xanthine/uracil permeases family protein	4.20E-07
O994_RS01375	EF3004		-2.3	sulfate transporter family protein	6.60E-08
O994_RS01240	EF3022		-3.3	sodium:dicarboxylate symporter family protein	2.70E-13
O994_RS01150	EF3041		-1.6	pheromone binding protein	3.40E-02
O994_RS01010	EF3069		-4.2	formate/nitrite transporter family protein	1.30E-13
**Metabolism of cofactors and vitamins**					
O994_RS12270	EF0449	*menH*	1.5	2-succinyl-6-hydroxy-2,4-cyclohexadiene-1-carboxylate synthase	3.70E-02
O994_RS10510	EF2569		3.1	hypothetical protein	1.30E-12
O994_RS02965	EF0848	*acpS*	-1.8	holo-ACP synthase	1.00E-03
O994_RS05245	EF1295	*ribF*	-2.1	riboflavin biosynthesis protein	7.60E-03
O994_RS08435	EF1969		-1.5	phosphomethylpyrimidine kinase	7.00E-02
O994_RS08880	EF2056	*ubiA*	-1.7	1,4-dihydroxy-2-naphthoate octaprenyltransferase	4.00E-04
**Metabolism of other amino acids**					
O994_RS09845	EF2392		1.6	class V aminotransferase	1.00E-03
O994_RS10500	EF2567	*selD*	2.9	selenide, water dikinase	3.20E-12
O994_RS10565	EF2581		3.7	selenate reductase subunit	2.20E-23
O994_RS11290	EF2738		1.7	thioredoxin reductase/glutathione-like protein	2.40E-04
O994_RS03365	EF0930	*metG*	-2.2	methionyl-tRNA synthetase	4.10E-08
**Metabolism of terpenoids and polyketides**					
O994_RS13875	EF0051	*ipk*	-2.4	4-diphosphocytidyl-2C-methyl-D-erythritol kinase	4.70E-08
O994_RS03235	EF0904	*mvk*	-2.1	mevalonate kinase	5.90E-04
**Nucleotide metabolism**					
O994_RS13845	EF0062		2.3	5'-nucleotidase	6.90E-09
O994_RS13635	EF0106	*arcC-1*	8.3	carbamate kinase	1.50E-110

O994_RS13390	EF0173	*pyn*	1.7	pyrimidine-nucleoside phosphorylase	3.20E-03
O994_RS13380	EF0175	*cdd*	2.3	cytidine deaminase	6.20E-07
O994_RS12570	EF0386	*arcC-2*	3.0	carbamate kinase	4.10E-03
O994_RS02910	EF0837		2.3	dihydroorotase	1.00E-04
O994_RS03890	EF1036	*ndk*	1.7	nucleoside diphosphate kinase	3.00E-03
O994_RS08225	EF1921	*rihC*	3.9	ribonucleoside hydrolase	8.60E-28
O994_RS10560	EF2580	*hydA*	3.1	phenylhydantoinase	7.50E-10
O994_RS14325	EF3293	*guaB*	1.6	inosine 5'-monophosphate dehydrogenase	2.40E-04
O994_RS13855	EF0058	*purR*	-3.3	pur operon repressor	6.70E-23
O994_RS13400	EF0171	*adD*	-2.0	adenosine deaminase	3.40E-03
O994_RS12975	EF0264	*hpt*	-1.7	hypoxanthine-guanine phosphoribosyltransferase	1.30E-02
O994_RS12155	EF0471	*nrdE*	-1.4	ribonucleotide-diphosphate reductase subunit α	5.50E-02
O994_RS02845	EF0825	*udk*	-4.3	uridine kinase	2.90E-22
O994_RS04520	EF1147	*pyrG*	-4.9	CTP synthetase	2.50E-57
O994_RS07375	EF1721	*pyrR*	-4.4	bifunctional pyrimidine regulatory protein	1.20E-14
O994_RS09565	EF2362	*purK-2*	-2.0	5-(carboxyamino)imidazole ribonucleotide synthase	1.40E-05
O994_RS09575	EF2364		-2.9	xanthine permease	6.70E-11
O994_RS09580	EF2365	*xpt*	-3.3	xanthine phosphoribosyltransferase	6.70E-10
O994_RS10445	EF2555	*tdk*	-2.7	thymidine kinase	2.00E-13
O994_RS00755	EF3127	*gmk*	-3.3	guanylate kinase	4.00E-26
O994_RS14320	EF3293	*guaB*	-2.5	inosine 5'-monophosphate dehydrogenase	1.10E-10
**Other**					
O994_RS13790	EF0071		2.5	lipoprotein	3.60E-11
O994_RS13770	EF0076		2.5	short chain dehydrogenase/reductase family oxidoreductase	1.60E-06
O994_RS13765	EF0077	*amaP*	1.9	alkaline shock response membrane anchor protein	6.20E-04
O994_RS13755	EF0079		1.6	gls24 protein	1.00E-02
O994_RS13750	EF0080		1.6	Asp23/Gls24 family envelope stress response protein	9.20E-03
O994_RS13745	EF0081		1.5	GlsB/YeaQ/YmgE family stress response membrane protein	8.20E-02
O994_RS12830	EF0277	*ogt*	3.1	methylated-DNA--protein-cysteine *S*-methyltransferase	6.30E-13
O994_RS12250	EF0453		2.2	organic hydroperoxide resistance protein	6.20E-09

O994_RS02150	EF0678		2.2	GNAT family *N*-acetyltransferase	1.70E-07
O994_RS01630	EF0706	*clpE*	3.0	ATP-dependent Clp protease, ATP-binding protein	2.10E-22
O994_RS02610	EF0766	*rapZ*	2.1	RNase adapter	2.00E-07
O994_RS02815	EF0809		1.8	GlsB/YeaQ/YmgE family stress response membrane protein	1.90E-03
O994_RS03105	EF0877		1.9	aldo/keto reductase family oxidoreductase	4.60E-05
O994_RS03870	EF1032	*drrC*	2.1	excinuclease ABC subunit	2.10E-05
O994_RS03880	EF1034		2.1	aminoglycoside phosphotransferase family protein	9.10E-05
O994_RS04185	EF1077		2.2	GNAT family *N*-acetyltransferase	7.20E-04
O994_RS04250	EF1092	*ebpB*	1.8	cell wall surface anchor family protein	6.70E-03
O994_RS04255	EF1093	*ebpC*	1.9	cell wall surface anchor family protein	9.70E-04
O994_RS04570	EF1157		1.8	Sapep family Mn(2^+)-dependent dipeptidase	2.40E-03
O994_RS04575	EF1158		3.8	N4-(β-*N*-acetylglucosaminyl)-L-asparaginase	1.30E-15
O994_RS04680	EF1180		2.0	CsbD family protein	5.80E-07
O994_RS04830	EF1211	*npr*	2.3	NADH peroxidase	7.40E-14
O994_RS05190	EF1284		3.9	structural protein	2.80E-25
O994_RS05195	EF1285		3.6	major tail protein	1.60E-20
O994_RS05465	EF1327		1.9	BadF/BadG/BcrA/BcrD ATPase	4.40E-06
O994_RS06080	EF1455	*terL*	2.1	phage terminase large subunit	1.40E-04
O994_RS06090	EF1457		1.6	minor head protein	5.40E-02
O994_RS06215	EF1481		2.0	tail fiber protein	1.90E-03
O994_RS07260	EF1698		1.8	NADPH-dependent FMN reductase domain-containing protein	6.00E-04
O994_RS07735	EF1796		1.5	lipoprotein	9.90E-02
O994_RS08500	EF1982	*uspA*	3.0	universal stress protein	3.40E-22
O994_RS09370	EF2217		3.1	α-1,2-mannosidase	1.70E-29
O994_RS09835	EF2390	*sufB*	1.4	Fe-S cluster assembly protein	8.70E-02
O994_RS09965	EF2419		1.9	kinase/pyrophosphorylase	1.00E-03
O994_RS10475	EF2562		2.8	flavodoxin	4.80E-10
O994_RS10555	EF2579	*dpaL*	2.7	diaminopropionate ammonia-lyase	4.40E-02
O994_RS11060	EF2689	*sbcC*	1.5	exonuclease	9.10E-02
O994_RS11065	EF2690	*sbcD*	1.5	exonuclease	9.50E-02
O994_RS11295	EF2739	*ahpC*	1.5	alkyl hydroperoxide reductase subunit C	3.10E-03

O994_RS11510	EF2785		1.9	oxidoreductase	4.90E-04
O994_RS01520	EF2972		1.7	amidinotransferase	5.50E-03
O994_RS01190	EF3032	*btpA*	2.5	photosystem I biogenesis protein	8.80E-09
O994_RS01175	EF3035		2.7	universal stress protein	4.60E-11
O994_RS01170	EF3036		2.8	thioredoxin family protein	9.40E-13
O994_RS00310	EF3233		1.6	Dps family protein	1.20E-02
O994_RS14275	EF3303		1.9	myosin-cross-reactive antigen	9.60E-06
O994_RS13065	EF0245		-2.2	decarboxylase	6.40E-05
O994_RS12965	EF0266		-3.6	chaperonin	1.30E-16
O994_RS12150	EF0472	*nrdI*	-1.4	ribonucleotide reductase stimulatory protein	6.10E-02
O994_RS12145	EF0473	*nrdH*	-1.7	ribonucleoside-diphosphate reductase 2	1.10E-03
O994_RS12125	EF0479		-2.1	phage integrase family site specific recombinase	4.20E-07
O994_RS02265	EF0700		-2.2	hemolysin	4.70E-09
O994_RS02325	EF0704		-1.5	lipoprotein	4.80E-02
O994_RS02370	EF0715	*tig*	-2.1	trigger factor	9.40E-08
O994_RS02490	EF0740		-3.0	deoxynucleoside kinase	4.00E-11
O994_RS02695	EF0783		-2.1	acetyltransferase	2.10E-06
O994_RS03015	EF0858	*pip*	-1.9	phage infection protein	2.40E-04
O994_RS04480	EF1139		-2.0	glutamine amidotransferase	1.30E-02
O994_RS04650	EF1173		-4.7	WecB/TagA/CpsF family glycosyl transferase	4.70E-05
O994_RS05010	EF1247		-2.5	iron-sulfur cluster biosynthesis family protein	1.70E-05
O994_RS05250	EF1296		-2.1	*N*-acetyltransferase	1.00E-03
O994_RS05490	EF1340		-2.9	pheromone cAM373 lipoprotein	2.90E-07
O994_RS06510	EF1538	*scpA*	-2.7	segregation and condensation protein A	1.80E-15
O994_RS06515	EF1539	*scpB*	-2.4	SMC-Scp complex subunit	1.00E-09
O994_RS06895	EF1619		-3.8	carbon dioxide concentrating mechanism protein	4.70E-10
O994_RS09290	EF2201		-2.2	flavodoxin	5.00E-06
O994_RS09535	EF2352	*lepA*	-2.3	GTP-binding protein	7.70E-11
O994_RS10090	EF2444		-3.2	acyl-CoA thioesterase	1.90E-17
O994_RS10310	EF2492		-1.6	glycosyl transferase group 2 family protein	2.40E-02

O994_RS10435	EF2553	*hemK*	-1.6	hemK protein	4.50E-02
O994_RS10440	EF2554	*prfA*	-1.7	peptide chain release factor 1	2.80E-04
O994_RS10655	EF2601		-2.9	acyl carrier protein	3.60E-14
O994_RS10930	EF2661		-4.1	diacylglycerol kinase catalytic subunit	3.50E-15
O994_RS10940	EF2664		-1.9	phosphoglycerate mutase	1.70E-02
O994_RS11240	EF2728		-2.3	lipoprotein	1.90E-05
O994_RS11300	EF2740		-2.3	*O*-methyltransferase	7.20E-07
O994_RS11660	EF2874		-2.8	haloacid dehalogenase (HAD) superfamily	6.70E-15
O994_RS11800	EF2899		-3.0	pyridine nucleotide-disulfide family oxidoreductase	6.30E-08
O994_RS01075	EF3056		-1.7	class A sortase	1.90E-02
O994_RS01045	EF3061	*mreD*	-3.0	rod shape-determining protein	5.00E-10
O994_RS00465	EF3191		-1.8	α/β hydrolase	8.10E-03
O994_RS14305	EF3296	*ychF*	-2.0	GTP-dependent nucleic acid-binding protein	1.40E-06
O994_RS14295	EF3298		-2.4	ParB family chromosome partitioning protein	4.90E-08
O994_RS14290	EF3299		-3.1	ParaA family ATPase	7.00E-17
**Poorly characterised**					
O994_RS13760	EF0078		1.9	DUF2273 domain-containing protein	8.10E-04
O994_RS12870	EF0269		2.3	PRD domain-containing protein	1.40E-11
O994_RS12820	EF0279		1.6	HD domain-containing protein	8.20E-02
O994_RS12580	EF0383		3.9	hypothetical protein	1.40E-27
O994_RS14960	EF0383		4.8	hypothetical protein	1.70E-64
O994_RS12545	EF0392		2.4	WxL domain-containing protein	9.40E-06
O994_RS12520	EF0397		1.6	DUF308 domain-containing protein	5.70E-03
O994_RS12475	EF0405		2.3	HAD superfamily hydrolase	2.80E-09
O994_RS02080	EF0665		1.5	hypothetical protein	4.60E-02
O994_RS02615	EF0767		1.5	YvcK family protein	4.40E-02
O994_RS02630	EF0770		1.4	DsbA family protein	8.10E-02
O994_RS02820	EF0819		1.7	DUF960 domain-containing protein	7.30E-02
O994_RS02865	EF0829		2.7	PRD domain-containing protein	2.80E-07
O994_RS02870	EF0830		2.7	glycine-rich SFCGS family protein	8.30E-07
O994_RS02880	EF0832		2.5	DUF4311 domain-containing protein	1.10E-04

O994_RS02885	EF0833	2.2	DUF4310 family protein	1.50E-03
O994_RS02920	EF0839	2.0	KDGP aldolase family protein	2.20E-02
O994_RS03430	EF0943	2.3	hypothetical protein	1.50E-06
O994_RS03740	EF1006	2.1	DUF4828 domain-containing protein	4.10E-06
O994_RS03775	EF1014	2.0	hypothetical protein	1.30E-04
O994_RS03780	EF1015	2.0	DUF3188 domain-containing protein	1.10E-03
O994_RS03810	EF1021	1.9	GNAT family *N*-acetyltransferase	5.00E-06
O994_RS03885	EF1035	2.7	DUF4767 domain-containing protein	9.10E-13
O994_RS04175	EF1074	2.3	hypothetical protein	5.90E-06
O994_RS04180	EF1075	2.7	*N*-acetyltransferase	1.10E-10
O994_RS04335	EF1109	2.4	iron-sulfur cluster binding protein	1.90E-11
O994_RS05050	EF1200	1.9	threonine/serine exporter family protein	2.60E-02
O994_RS05200	EF1286	3.7	hypothetical protein	1.60E-16
O994_RS05210	EF1288	4.3	hypothetical protein	1.30E-13
O994_RS05595	EF1362	1.7	hypothetical protein	4.40E-03
O994_RS05630	EF1368	2.5	hypothetical protein	1.50E-14
O994_RS06085	EF1456	2.2	DUF1073 domain-containing protein	1.00E-05
O994_RS06100	EF1459	2.0	hypothetical protein	1.00E-03
O994_RS06110	EF1461	2.3	DUF2213 domain-containing protein	4.90E-08
O994_RS06115	EF1462	2.2	hypothetical protein	1.00E-06
O994_RS06120	EF1463	2.5	DUF2184 domain-containing protein	1.80E-08
O994_RS06125	EF1464	2.6	hypothetical protein	1.20E-05
O994_RS06135	EF1465	2.2	hypothetical protein	1.70E-06
O994_RS06140	EF1467	2.1	hypothetical protein	2.10E-05
O994_RS06145	EF1468	2.2	hypothetical protein	2.10E-06
O994_RS06150	EF1469	2.1	DUF3383 family protein	6.00E-07
O994_RS06155	EF1470	1.8	hypothetical protein	1.40E-02
O994_RS06160	EF1471	2.4	hypothetical protein	5.70E-05
O994_RS06615	EF1560	1.8	hypothetical protein	3.10E-04
O994_RS07065	EF1657	3.9	hypothetical protein	5.50E-22
O994_RS07165	EF1677	2.6	hypothetical protein	8.70E-08

O994_RS07310	EF1708		1.8	putative glycosyl hydrolase	9.60E-05
O994_RS07730	EF1794		1.5	DUF2188 domain-containing protein	7.30E-02
O994_RS09310	EF2205		2.3	hypothetical protein	9.70E-06
O994_RS09330	EF2209		1.9	hypothetical protein	3.30E-04
O994_RS09480	EF2239		2.1	hypothetical protein	3.60E-04
O994_RS09840	EF2391		1.6	SUF system NifU family Fe-S cluster assembly protein	1.00E-02
O994_RS09850	EF2393	*sufD*	1.5	Fe-S cluster assembly protein	5.20E-03
O994_RS10075	EF2441		1.6	DUF47 family protein	1.90E-02
O994_RS10240	EF2477		1.6	SPFH domain-containing protein	4.90E-03
O994_RS10480	EF2563		2.4	selenium-dependent molybdenum hydroxylase system protein	6.20E-05
O994_RS10485	EF2564		2.3	hydroxylase accessory protein	1.10E-05
O994_RS10490	EF2565		2.3	DUF3343 domain-containing protein	6.60E-04
O994_RS10495	EF2566		2.6	hypothetical protein	2.40E-07
O994_RS10515	EF2570		3.3	aldehyde oxidoreductase	4.10E-21
O994_RS10570	EF2582		4.2	chlorohydrolase/aminohydrolase	7.30E-30
O994_RS11115	EF2700		1.7	MutT/nudix family protein	1.20E-03
O994_RS11135	EF2702		1.6	hypothetical protein	7.90E-02
O994_RS11180	EF2713		2.0	cell wall surface anchor family protein	5.30E-06
O994_RS11515	EF2786		2.3	DUF488 domain-containing protein	6.90E-09
O994_RS11615	EF2864		1.4	putative lipoprotein	9.00E-02
O994_RS01245	EF3021	*gst*	1.6	DinB family glutathione transferase	4.10E-02
O994_RS00985	EF3075		1.6	WxL domain-containing protein	1.80E-02
O994_RS00745	EF3130		1.5	hypothetical protein	2.20E-02
O994_RS00735	EF3132		1.5	hypothetical protein	8.50E-02
O994_RS14160	EF3326		4.7	hypothetical protein	1.40E-58
O994_RS12990	EF0261		-2.5	hypothetical protein	2.30E-15
O994_RS12650	EF0369		-1.8	HAD superfamily hydrolase	1.70E-03
O994_RS12205	EF0462		-2.1	hypothetical protein	3.00E-03
O994_RS12170	EF0468		-2.2	LemA family protein	3.00E-05
O994_RS12165	EF0469		-2.3	hypothetical protein	1.10E-07

O994_RS02605	EF0764		-3.8	hypothetical protein	9.00E-09
O994_RS03010	EF0857		-2.8	hypothetical protein	1.50E-06
O994_RS03245	EF0906		-1.8	FUSC family protein	2.50E-02
O994_RS03305	EF0918		-2.6	DUF1304 domain-containing protein	6.80E-06
O994_RS03475	EF0953		-3.1	hypothetical protein	3.70E-10
O994_RS03945	EF1047		-1.9	DUF177 domain-containing protein	3.00E-05
O994_RS04510	EF1145		-2.2	DUF1934 domain-containing protein	2.80E-06
O994_RS04615	EF1166		-2.0	YitT family protein	2.10E-04
O994_RS04985	EF1241		-3.0	DUF5067 domain-containing protein	3.30E-08
O994_RS05005	EF1246		-1.8	DUF441 domain-containing protein	4.50E-02
O994_RS05015	EF1248		-2.0	hypothetical protein	2.10E-06
O994_RS05095	EF1265		-4.7	RNA binding protein	3.10E-52
O994_RS05260	EF1298		-1.8	DUF1048 domain-containing protein	8.00E-03
O994_RS05395	EF1315		-1.9	hypothetical protein	8.60E-03
O994_RS05615	EF1365		-2.0	AI-2E family transporter	1.40E-04
O994_RS05620	EF1366		-2.2	hypothetical protein	6.00E-06
O994_RS05850	EF1412		-2.1	hypothetical protein	6.40E-04
O994_RS05885	EF1419		-1.8	hypothetical protein	3.90E-03
O994_RS05890	EF1420		-2.0	hypothetical protein	3.30E-05
O994_RS05895	EF1421		-2.7	ImmA/IrrE family metallo-endopeptidase	4.10E-11
O994_RS06500	EF1536		-2.1	α/β hydrolase	4.20E-05
O994_RS06530	EF1542		-2.2	hypothetical protein	2.30E-04
O994_RS06845	EF1609		-2.2	DUF2179 domain-containing protein	9.80E-04
O994_RS06900	EF1620		-3.4	hypothetical protein	3.80E-09
O994_RS07415	EF1730		-1.8	EbsC protein	3.10E-03
O994_RS07435	EF1734		-3.2	YneF family protein	2.80E-15
O994_RS07620	EF1771		-1.8	yigZ family protein	6.50E-03
O994_RS07625	EF1772		-1.8	DUF2829 domain-containing protein	4.70E-02
O994_RS08200	EF1916	*engB*	-3.2	GTP-binding protein	3.10E-13
O994_RS08925	EF2065		-4.2	DUF1033 family protein	2.30E-15
O994_RS09340	EF2211		-2.1	yxeA family protein	4.00E-04

O994_RS09345	EF2212		-1.8	nucleoid-associated protein	4.30E-03
O994_RS09180	EF2492		-2.0	glycosyl transferase group 2 family protein	1.20E-05
O994_RS10345	EF2499		-2.8	hypothetical protein	2.00E-12
O994_RS10680	EF2606		-3.2	DUF1146 domain-containing protein	4.90E-14
O994_RS11775	EF2893		-2.6	hypothetical protein	5.50E-08
O994_RS01260	EF3018		-2.2	hypothetical protein	5.10E-06
O994_RS01020	EF3067		-2.1	HAD superfamily hydrolase	3.60E-05
O994_RS00975	EF3078		-1.9	DUF1054 domain-containing protein	1.00E-02
O994_RS00970	EF3079		-1.8	GNAT family *N*-acetyltransferase	1.00E-02
O994_RS00915	EF3091		-1.8	YitT family protein	1.20E-02
O994_RS00670	EF3146		-3.2	hypothetical protein	3.30E-14
O994_RS00610	EF3160		-2.7	hypothetical protein	3.40E-06
O994_RS00535	EF3176		-3.9	YccF domain-containing protein	8.60E-11
O994_RS00530	EF3177		-1.9	hypothetical protein	7.00E-03
O994_RS00240	EF3247		-2.1	putative holin-like toxin	3.60E-03
O994_RS00190	EF3259		-1.6	hypothetical protein	3.30E-02
O994_RS14310	EF3295		-1.6	DUF1129 domain-containing protein	3.30E-03
O994_RS14300	EF3297		-2.4	DUF951 domain-containing protein	1.30E-08
**Replication and repair**					
O994_RS12825	EF0278	*tag-1*	2.7	DNA-3-methyladenine glycosylase	4.90E-09
O994_RS03575	EF0972		1.7	DNA repair exonuclease	3.30E-02
O994_RS08485	EF1979		1.7	recombination factor protein	3.30E-03
O994_RS14085	EF0008	*ssb-1*	-1.5	Single-strand binding protein	2.50E-02
O994_RS13810	EF0066	*ruvA*	-2.0	Holliday junction DNA helicase	2.90E-03
O994_RS04595	EF1162		-1.5	helicase	4.10E-02
O994_RS05415	EF1319		-1.6	DNA/RNA non-specific endonuclease	8.30E-02
O994_RS06460	EF1527	*obgE*	-2.5	GTPase	2.60E-10
O994_RS06875	EF1615	*parE*	-1.5	DNA topoisomerase IV subunit B	7.40E-02
O994_RS07030	EF1650	*topA*	-2.1	DNA topoisomerase I	1.10E-05
O994_RS07040	EF1652		-2.3	DNA-protecting protein	3.30E-04

O994_RS07045	EF1653	*rnhB*	-3.0	ribonuclease HII	5.00E-12
O994_RS10935	EF2663		-1.5	recombinase D	7.00E-02
O994_RS01015	EF3068		-2.5	DNA alkylation repair protein	6.60E-08
**Signal transduction**					
O994_RS04810	EF1206		3.6	malate dehydrogenase, decarboxylating	2.10E-17
O994_RS04815	EF1207	*maeP*	4.9	CCS family citrate carrier protein	2.30E-44
O994_RS01215	EF3027	*htrA*	1.9	serine protease	1.90E-06
O994_RS00455	EF3193	*lrgB*	6.0	antiholin-like protein	2.70E-88
O994_RS00450	EF3194	*lrgA*	6.7	murein hydrolase regulator	2.20E-92
O994_RS14335	EF3289	*croR*	4.5	DNA-binding response regulator	5.80E-45
O994_RS14330	EF3290	*croS*	4.7	sensor histidine kinase	9.00E-50
O994_RS14200	EF3318	*citX*	3.3	2-(5''-triphosphoribosyl)-3'-dephosphocoenzyme-A synthase	3.90E-18
O994_RS14195	EF3319	*citF*	3.7	citrate lyase subunit α	1.30E-21
O994_RS14190	EF3320	*citE*	3.8	citrate lyase subunit β	4.60E-07
O994_RS14185	EF3321	*citD*	4.1	citrate lyase subunit γ	4.60E-30
O994_RS14180	EF3322	*citC*	4.2	[citrate (pro-3S)-lyase] ligase	1.60E-43
O994_RS03005	EF0856		-2.3	aldo/keto reductase	2.40E-05
O994_RS03960	EF1050		-2.2	DNA-binding response regulator	1.20E-06
O994_RS03965	EF1051		-2.2	sensor histidine kinase	2.60E-05
O994_RS08855	EF2050		-2.6	ABC transporter ATP-binding protein	2.40E-07
O994_RS09095	EF2159	*glnA*	-2.3	glutamine synthetase, type I	4.80E-13
O994_RS09375	EF2218		-1.6	AraC family transcriptional regulator	5.30E-02
**Transcription**					
O994_RS04515	EF1146	*rpoE*	-1.8	DNA-directed RNA polymerase subunit δ	1.50E-04
O994_RS11875	EF2914	*greA*	-2.8	transcription elongation factor	2.60E-13
O994_RS00760	EF3126	*rpoZ*	-2.6	DNA-directed RNA polymerase subunit ω	8.20E-08
**Transcriptional regulators**					
O994_RS13630	EF0107		7.4	Crp/Fnr family transcriptional regulator	2.90E-29
O994_RS02070	EF0663		2.3	YebC/PmpR family DNA-binding transcriptional regulator	4.00E-13
O994_RS02450	EF0731		1.9	LuxR family transcriptional regulator	3.50E-04

O994_RS02860	EF0828		2.3	transcription antiterminator	6.10E-06
O994_RS03125	EF0881	*nrdR*	1.5	transcriptional regulator	1.50E-02
O994_RS04565	EF1156		2.1	GntR family transcriptional regulator	1.90E-05
O994_RS04820	EF1209		2.5	sensory box histidine kinase	1.00E-10
O994_RS04825	EF1210		2.4	response regulator	1.80E-06
O994_RS05570	EF1357		2.2	AraC family transcriptional regulator	3.40E-05
O994_RS06760	EF1591		2.4	AraC family transcriptional regulator	4.40E-10
O994_RS07060	EF1656		4.7	LysR family transcriptional regulator	1.40E-32
O994_RS07315	EF1709		2.0	GntR family transcriptional regulator	4.70E-05
O994_RS01690	EF1839	*lacR*	1.9	lactose phosphotransferase system repressor	1.00E-04
O994_RS08375	EF1955		1.9	σ-54 dependent DNA-binding response regulator	8.70E-05
O994_RS12030	EF2966		3.8	BglG family transcriptional antiterminator	1.30E-31
O994_RS00355	EF3216		2.3	σ-54-interacting transcriptional regulator	1.00E-08
O994_RS14250	EF3308	*srlR*	4.2	transcriptional regulator	2.20E-16
O994_RS14245	EF3309	*srlM*	5.2	transcriptional activator	4.60E-40
O994_RS14150	EF3328	*citO*	3.3	GntR family transcriptional regulator	2.80E-25
O994_RS13780	EF0074		-4.1	Crp/Fnr family transcriptional regulator	3.80E-13
O994_RS12485	EF0403		-1.9	MarR family transcriptional regulator	1.10E-02
O994_RS12400	EF0421		-3.6	MerR family transcriptional regulator	1.60E-09
O994_RS12395	EF0422		-3.1	IclR family transcriptional regulator	1.10E-10
O994_RS12190	EF0465		-1.5	transcriptional regulator	4.40E-02
O994_RS03735	EF1005		-1.9	metal-dependent transcriptional regulator	7.10E-04
O994_RS05255	EF1297		-1.9	PadR family transcriptional regulator	7.00E-03
O994_RS05500	EF1342		-2.8	MarR family transcriptional regulator	7.70E-07
O994_RS05900	EF1422		-2.6	Cro/CI family transcriptional regulator	1.00E-06
O994_RS06450	EF1525		-1.6	FUR family transcriptional regulator	2.60E-02
O994_RS06660	EF1569	*psr*	-1.9	transcriptional regulator	2.30E-04
O994_RS07485	EF1741	*ccpA*	-2.5	catabolite control protein A	3.50E-13
O994_RS07955	EF1864		-3.3	DNA-binding response regulator	4.30E-10
O994_RS09100	EF2160	*glnR*	-2.4	regulatory protein	2.30E-12

O994_RS10010	EF2428		-1.5	PadR family transcriptional regulator	8.50E-02
O994_RS10895	EF2653		-2.6	Cro/CI family transcriptional regulator	1.60E-09
O994_RS11245	EF2729	*nusG*	-3.0	transcription antitermination protein	2.50E-15
O994_RS11460	EF2774		-1.9	helix-turn-helix transcriptional regulator	5.80E-03
O994_RS11720	EF2886		-1.8	MarR family transcriptional regulator	1.80E-05
O994_RS12010	EF2962		-1.8	LacI family sugar-binding transcriptional regulator	1.30E-02
**Translation**					
O994_RS13665	EF0100	*serS-1*	2.4	seryl-tRNA synthetase	2.10E-12
O994_RS02825	EF0820	*rplY*	3.4	50S ribosomal protein L25	5.90E-34
O994_RS02915	EF0838		2.0	DgaE family pyridoxal phosphate-dependent ammonia lyase	3.90E-03
O994_RS07595	EF1764	*yfiA*	2.1	ribosomal subunit interface protein	1.40E-08
O994_RS14090	EF0007	*rpsF*	-1.6	30S ribosomal protein S6	1.50E-02
O994_RS12985	EF0262		-2.9	RNA binding protein	1.10E-18
O994_RS12960	EF0267		-2.5	zinc-binding NifR3 family TIM-barrel protein	3.10E-09
O994_RS01920	EF0633	*tryS-1*	-1.5	tyrosyl-tRNA synthetase	5.00E+00
O994_RS02155	EF0679		-2.0	RluA family pseudouridine synthase	1.50E-03
O994_RS02220	EF0691	*trmB*	-1.9	tRNA (guanine-N(7)-)-methyltransferase	2.40E-03
O994_RS02270	EF0701	*prfc*	-2.9	peptide chain release factor 3	2.30E-13
O994_RS02830	EF0821		-2.3	16S rRNA pseudouridine(516) synthase	2.80E-06
O994_RS03065	EF0868	*queA*	-1.9	S-adenosylmethionine--tRNA ribosyltransferase-isomerase	1.30E-05
O994_RS03200	EF0896	*tgt*	-3.3	queuine tRNA-ribosyltransferase	7.30E-24
O994_RS03285	EF0914	*infC*	-3.6	translation initiation factor IF-3	1.20E-32
O994_RS03290	EF0915	*rpmI*	-2.7	50S ribosomal protein L35	3.40E-14
O994_RS03295	EF0916	*rplT*	-2.2	50S ribosomal protein L20	1.40E-08
O994_RS03555	EF0968	*rplU*	-1.8	50S ribosomal protein L21	4.20E-04
O994_RS03560	EF0969		-1.7	ribosomal-processing cysteine protease	7.30E-04
O994_RS03565	EF0970	*rpmA*	-1.5	50S ribosomal protein L27	1.00E-02
O994_RS03950	EF1048	*rpmF-3*	-1.6	50S ribosomal protein L32	1.80E-02
O994_RS06520	EF1540		-2.1	rRNA pseudouridine synthase	1.10E-06
O994_RS07465	EF1739	*tryS-2*	-1.9	tyrosyl-tRNA synthetase	4.30E-05

O994_RS08120	EF1898	*rplS*	-1.5	50S ribosomal protein L19	5.80E-02
O994_RS08125	EF1899	*trmD*	-2.0	tRNA (guanine-N1)-methyltransferase	1.10E-05
O994_RS08130	EF1900	*rimM*	-2.1	16S rRNA processing protein	3.50E-05
O994_RS08845	EF2048	*rlmN*	-3.3	23S rRNA (adenine(2503)-C(2))-methyltransferase	2.60E-13
O994_RS09875	EF2398	*rpsB*	-1.6	30S ribosomal protein S2	4.40E-03
O994_RS09955	EF2416	*rpsU*	-1.5	30S ribosomal protein S21	2.60E-02
O994_RS10085	EF2443	*rpsT*	-1.9	30S ribosomal protein S20	8.30E-05
O994_RS10220	EF2473	*rimI*	-1.7	ribosomal-protein-alanine acetyltransferase	2.20E-02
O994_RS10225	EF2474	*rimI*	-1.7	ribosomal-protein-alanine acetyltransferase	9.20E-03
O994_RS10230	EF2475	*tsaB*	-1.6	tRNA (adenosine(37)-N6)-threonylcarbamoyltransferase	6.00E-02
O994_RS10945	EF2665	*trmL*	-2.2	RNA methyltransferase	1.90E-07
O994_RS11010	EF2679	*trpS*	-2.5	tryptophanyl-tRNA synthetase	1.30E-08
O994_RS11255	EF2731	*rpmG*	-3.9	50S ribosomal protein L33	1.80E-16
O994_RS11575	EF2856	*rpmG*	-2.5	50S ribosomal protein L33	3.40E-11
O994_RS01030	EF3065	*rpsO*	-3.1	30S ribosomal protein S15	1.70E-15
O994_RS01005	EF3070	*rpsD*	-3.0	30S ribosomal protein S4	1.10E-17
O994_RS00810	EF3116	*rpmB*	-2.1	50S ribosomal protein L28	7.80E-04
O994_RS00390	EF3207		-3.3	dihydrouridine synthase	2.90E-16
O994_RS00320	EF3230	*rpsI*	-1.6	30S ribosomal protein S9	1.50E-03
O994_RS00315	EF3231	*rplM*	-2.0	50S ribosomal protein L13	3.10E-07
O994_RS14285	EF3300	*gidB*	-3.9	16S rRNA methyltransferase	3.30E-21
O994_RS14125	EF3333	*rpmH*	-2.8	50S ribosomal protein L34	1.50E-09
**Unclassified genetic information processing**					
O994_RS05625	EF1367		-1.8	cold-shock protein	2.60E-02
**Xenobiotics biodegradation and metabolism**					
O994_RS02835	EF0822		-2.0	HAD superfamily hydrolase	8.20E-04
O994_RS10615	EF2591		-2.0	glyoxalase	1.50E-06
**JH2-2 Specific Genes**					
O994_RS01325			1.9	hypothetical protein	8.90E-03
O994_RS01355			3.4	hypothetical protein	8.80E-14
O994_RS02075			4.1	hypothetical protein	6.60E-36

O994_RS03970	*lacC*	3.0	tagatose-6-phosphate kinase	9.20E-13
O994_RS05055		3.4	hypothetical protein	2.20E-33
O994_RS05240		5.6	PTS sugar transporter subunit IIB	5.20E-28
O994_RS06725		5.5	PTS fructose transporter subunit IIA	4.40E-21
O994_RS08875		1.7	hypothetical protein	1.80E-03
O994_RS11760		2.2	type I restriction endonuclease subunit R	9.70E-12
O994_RS14595		3.7	hypothetical protein	2.40E-27
O994_RS14700		1.9	hypothetical protein	1.70E-03
O994_RS14860		2.1	hypothetical protein	6.60E-06
O994_RS14955		3.4	hypothetical protein	2.30E-32
O994_RS15000		2.0	hypothetical protein	8.00E-04
O994_RS01705		-2.4	tRNA-Ser	2.10E-04
O994_RS01800		-2.3	tRNA-Ala	9.00E-04
O994_RS01805		-1.6	16S ribosomal RNA	4.50E-02
O994_RS03080		-2.9	α/β hydrolase	5.00E-07
O994_RS03850		-1.5	type I restriction-modification system subunit M	1.70E-02
O994_RS04000		-4.4	hypothetical protein	5.50E-62
O994_RS08365		-2.7	tRNA-Ala	8.40E-15
O994_RS08370		-2.8	tRNA-Ala	9.80E-15
O994_RS09570		-1.9	hypothetical protein	1.80E-02
O994_RS11420		-3.1	MFS transporter	3.70E-08
O994_RS11450		-2.4	tRNA-Met	1.70E-04
O994_RS12890		-2.6	hypothetical protein	2.00E-10
O994_RS12895		-2.8	hypothetical protein	5.70E-06
O994_RS12900		-2.6	IS30-like element IS6770 family transposase	1.60E-07
O994_RS14175	*truB*	-3.7	tRNA pseudouridine(55) synthase	5.20E-09
O994_RS14340		-1.6	NAD(P)/FAD-dependent oxidoreductase	5.80E-03
O994_RS14445		-2.0	hypothetical protein	1.30E-06
O994_RS14525		-2.1	hypothetical protein	9.50E-04
O994_RS14530		-2.2	hypothetical protein	5.00E-05

O994_RS14575		-4.6	hypothetical protein	2.10E-67
O994_RS14615		-1.8	hypothetical protein	4.30E-02
O994_RS14660		-3.7	MFS transporter	1.40E-12
O994_RS14750		-1.7	hypothetical protein	6.30E-03
O994_RS14820		-4.7	hypothetical protein	9.80E-15
O994_RS14865		-1.8	hypothetical protein	6.30E-04
O994_RS14970		-2.1	hypothetical protein	2.70E-09
O994_RS14975		-2.5	recombinase family protein	3.40E-05
O994_RS15030	*recR*	-3.1	recombination protein	2.60E-08

Table S2. Genes differentially expressed in *E. faecalis* wild-type versus Δ*croRS* in the presence of TXB.

JH2-2 Locus Tag	V583 Gene	Gene Name	F/C (log_2)	Gene Function	P_{adj}
**Amino acid**					
O994_RS13945	EF0037	*proA*	2.5	γ-glutamyl phosphate reductase	5.60E-17
O994_RS13940	EF0038	*proB*	3.2	glutamate-5-kinase	6.60E-31
O994_RS13645	EF0104	*arcA*	4.8	arginine deaminase	2.20E-17
O994_RS13640	EF0105	*argF-1*	5.0	ornithine carbamoyltransferase	5.90E-20
O994_RS13635	EF0106	*arcC-1*	2.6	carbamate kinase I	1.20E-04
O994_RS12655	EF0368		2.6	aspartate kinase	3.00E-09
O994_RS02475	EF0737		1.4	amidase	8.20E-02
O994_RS02700	EF0784	*metK*	2.0	*S*-adenosylmethionine synthetase	2.60E-06
O994_RS03895	EF1037	*aspD*	2.3	aspartate aminotransferase	1.30E-13
O994_RS04455	EF1133	*dapD*	4.3	2,3,4,5-tetrahydropyridine-2,6-dicarboxylate *N*-acetyltransferase	2.10E-115
O994_RS04460	EF1134		3.7	M20/M25/M40 family peptidase	6.30E-61
O994_RS04695	EF1183	*asd*	1.8	aspartate-semialdehyde dehydrogenase	4.50E-04
O994_RS04700	EF1184	*dapA*	1.7	4-hydroxy-tetrahydrodipicolinate synthase	2.60E-05
O994_RS05390	EF1314	*alaT*	4.9	aminotransferase	2.10E-115
O994_RS05865	EF1415	*gdhA*	4.1	glutamate dehydrogenase	9.50E-55
O994_RS06620	EF1561	*aroE*	1.6	shikimate 5-dehydrogenase	4.40E-02
O994_RS06625	EF1562		1.8	3-deoxy-7-phosphoheptulonate synthase	3.20E-04
O994_RS06630	EF1563	*aroB*	2.1	3-dehydroquinate synthase	8.30E-06
O994_RS06635	EF1564	*aroC*	2.4	chorismate synthase	4.40E-10
O994_RS06640	EF1565		1.7	prephenate dehydrogenase	1.30E-02
O994_RS06875	EF1618	*eutH*	2.5	ethanolamine utilization protein	5.90E-11
O994_RS06975	EF1638	*pduV*	2.4	propanediol utilization protein	4.90E-03
O994_RS07300	EF1706		1.7	aromatic amino acid aminotransferase	2.10E-04
O994_RS07490	EF1743	*pepQ-2*	1.5	proline dipeptidase	6.00E-02
O994_RS09355	EF2214		4.4	glyoxylase	1.10E-55
O994_RS09970	EF2420	*thrB*	1.4	homoserine kinase	7.80E-02
O994_RS09975	EF2421	*thrC*	2.6	threonine synthase	1.70E-18

O994_RS09980	EF2422	*hom*	2.6	homoserine dehydrogenase	1.00E-18
O994_RS09990	EF2424	*proC*	2.3	pyrroline-5-carboxylate reductase	2.80E-11
O994_RS11210	EF2721	*sdhb-2*	3.9	L-serine dehydratase, iron-sulfur dependent subunit β	2.30E-44
O994_RS11215	EF2722	*sdha-2*	3.1	iron-sulfur-dependent L-serine dehydratase subunit α	1.20E-19
O994_RS03510	EF0961	*proC*	-2.3	pyrroline-5-carboxylate reductase	3.60E-05
O994_RS04600	EF1163		-1.9	L-asparaginase	1.40E-05
O994_RS05990	EF2340	*dcm*	-1.9	C-5 cytosine-specific DNA methylase	4.20E-02
**Autolysis**					
O994_RS13030	EF0252		2.6	*N*-acetylmuramoyl-L-alanine amidase	7.50E-17
O994_RS12300	EF0443		3.8	LysM domain-containing protein	2.80E-31
O994_RS02775	EF0799		1.8	autolysin	5.90E-04
O994_RS06420	EF1518		5.1	soluble lytic murein transglycosylase	5.30E-88
**Carbohydrate metabolism**					
O994_RS13020	EF0255	*ldh-1*	2.2	L-lactate dehydrogenase	2.40E-09
O994_RS03215	EF0900	*adhE*	2.5	bifunctional acetaldehyde-CoA/alcohol dehydrogenase	1.20E-06
O994_RS03490	EF0956	*pgmB*	1.5	β-phosphoglucomutase	2.10E-02
O994_RS03495	EF0957		2.3	maltose phosphorylase	1.30E-09
O994_RS04160	EF1071	*galT*	1.7	galactose-1-phosphate uridylyltransferase	6.80E-03
O994_RS05535	EF1349		1.9	α-glucosidase	1.20E-03
O994_RS06865	EF1613	*pflB*	4.9	formate acetyltransferase	7.80E-07
O994_RS07000	EF1644	*lacX*	2.5	aldose-1-epimerase	6.40E-17
O994_RS09090	EF2158	*nifJ*	1.8	pyruvate ferredoxin/flavodoxin oxidoreductase family protein	4.10E-03
O994_RS09995	EF2425		2.6	phospho-sugar mutase	2.90E-27
O994_RS10030	EF2432		1.8	metallo-β-lactamase superfamily protein	1.10E-06
O994_RS10155	EF2456	*pycA*	1.4	pyruvate carboxylase	7.80E-02
O994_RS10350	EF2500		3.9	GcvH family protein	1.40E-38
O994_RS10605	EF2589	*manA*	1.7	mannose-6-phosphate isomerase	5.20E-03
O994_RS10940	EF2664		1.9	histidine phosphatase family protein	3.70E-03
O994_RS11675	EF2877	*accC*	1.4	acetyl-CoA carboxylase biotin carboxylase subunit	4.90E-02
O994_RS11885	EF2916		1.9	HAD family hydrolase	1.00E-04

O994_RS00300	EF3235		3.2	gluconokinase	3.30E-37
O994_RS02385	EF0718	*fruk-2*	-2.9	1-phosphofructokinase	3.10E-11
O994_RS06325	EF1503	*fbp*	-1.5	fructose-1,6-bisphosphatase	9.60E-02
O994_RS06830	EF1606		-2.2	glycosyl hydrolase	4.70E-05
O994_RS14270	EF3304		-2.2	fructose-6-phosphate aldolase	3.10E-06
**Cellular community**					
O994_RS00880	EF3106	*opp2A*	3.0	oligopeptide ABC transporter substrate-binding protein	3.90E-30
O994_RS00875	EF3107	*opp2C*	3.7	oligopeptide ABC transporter permease	5.90E-41
O994_RS00860	EF3110	*opp2D*	3.5	oligopeptide ABC transporter ATP-binding protein	3.30E-26
**Drug resistance: antimicrobial**					
O994_RS02970	EF0849	*alr*	-2.6	alanine racemase	1.30E-17
O994_RS06490	EF1534		-3.4	cyclophilin type peptidyl-prolyl cis-trans isomerase	2.40E-29
**Energy Metabolism**					
O994_RS06855	EF1611	*ppaC*	2.0	manganese-dependent inorganic pyrophosphatase	2.30E-07
O994_RS08875	EF2055		-2.3	pyridine nucleotide-disulfide family oxidoreductase	2.50E-12
**Folding, sorting and degradation**					
O994_RS02185	EF0685	*prsA*	1.4	peptidyl-prolyl cis-trans isomerase	6.20E-02
O994_RS03205	EF0897	*yajC*	1.7	preprotein translocase subunit	3.00E-03
O994_RS07010	EF1646	*hslU*	3.1	heat shock protein	3.30E-30
O994_RS07015	EF1647	*hslV*	2.6	heat shock protein	2.60E-15
O994_RS00900	EF3094	*ftsY*	1.7	signal recognition particle-docking protein	6.30E-04
O994_RS05675	EF1377		-1.8	DEAD/DEAH box helicase	7.10E-05
O994_RS10750	EF2620	*secG*	-1.7	preprotein translocase subunit	4.50E-03
**Glycan biosynthesis and metabolism**					
O994_RS02160	EF0680		2.7	penicillin-binding protein 2A	1.50E-13
O994_RS02520	EF0746	*pbp(6)*	5.4	penicillin binding protein	1.50E-77
O994_RS03665	EF0991	*pbpC*	2.8	penicillin-binding protein C	2.00E-22

O994_RS03670	EF0992	*mraY*	2.1	phospho-*N*-acetylmuramoyl-pentapeptide-transferase	1.70E-07
O994_RS03675	EF0993	*murD*	1.9	UDP-*N*-acetylmuramoyl-L-alanyl-D-glutamate synthetase	5.90E-04
O994_RS03680	EF0994	*murG*	2.1	undecaprenyldiphospho-muramoylpentapeptide β-*N*-acetylglucosaminyltransferase	1.50E-07
O994_RS04525	EF1148	*pbp1A*	2.6	penicillin-binding protein 1A	2.60E-24
O994_RS05270	EF1300		2.9	putative lipid II flippase	5.70E-10
O994_RS06105	EF1460		3.0	LysM peptidoglycan-binding domain-containing protein	5.10E-08
O994_RS06550	EF1546		1.9	putative teichoic acid transport protein	1.20E-08
O994_RS07470	EF1740	*pbp1B*	2.2	penicillin-binding protein 1B	2.00E-12
O994_RS07505	EF1746	*galU*	2.6	UTP-glucose-1-phosphate uridylyltransferase	8.10E-20
O994_RS09050	EF2150		2.1	FemAB family protein	2.30E-11
O994_RS09150	EF2170	*epaX*	2.1	putative glycosyl transferase family 2	7.30E-07
O994_RS09155	EF2171	*epaW*	2.8	dTDP-4-dehydro-6-deoxy-D-glucose	3.70E-21
O994_RS09160	EF2172	*ispD*	2.9	2-C-methyl-D-erythritol 4-phosphate	1.40E-20
O994_RS09170	EF2174		3.0	putative L-alanyl-D-glutamate peptidase	1.20E-11
O994_RS09190	EF2178	*epaQ*	1.5	EpaQ family protein	9.40E-03
O994_RS09240	EF2191	*epaH*	1.4	dTDP-4-dehydrorhamnose reductase	6.20E-02
O994_RS09245	EF2192	*epaG*	2.2	dTDP-glucose 4,6-dehydratase	5.30E-13
O994_RS09250	EF2193	*epaF*	2.7	dTDP-4-dehydrorhamnose 3,5-epimerase	7.30E-27
O994_RS09255	EF2194	*epaE*	2.6	glucose-1-phosphate thymidylyltransferase	4.00E-22
O994_RS09260	EF2195	*epaD*	1.9	α-1,3-L-rhamnosyltransferase	4.50E-06
O994_RS09265	EF2196	*epaC*	1.9	glycosyl transferase family 2	7.50E-06
O994_RS09270	EF2197	*epaB*	1.9	putative α-D-GlcNAc-pyrophosphate polyprenol, α-3-L-rhamnosyl transferase	2.40E-05
O994_RS10235	EF2476	*pbp4(5)*	4.2	penicillin-binding protein 4	2.20E-79
O994_RS10585	EF2585	*murT*	3.3	mur ligase	1.50E-56
O994_RS10590	EF2586	*gatD*	3.0	glutamine amidotransferase	6.40E-34
O994_RS10920	EF2658		2.8	FemAB family protein	6.00E-25
O994_RS11265	EF2733	*murB*	2.7	UDP-*N*-acetylenolpyruvoylglucosamine reductase	3.60E-19
O994_RS11580	EF2857	*pbp2B*	4.8	penicillin-binding protein 2B	3.00E-85
O994_RS11595	EF2860	*pbp*	2.5	putative peptidoglycan transpeptidase	1.10E-18
O994_RS01055	EF3060	*salA*	3.2	peptidoglycan DL-endopeptidase	2.90E-13

O994_RS13605	EF0114		-2.0	glycosyl hydrolase	4.70E-03
O994_RS02085	EF0666		-1.5	glyoxalase	2.40E-02
O994_RS10285	EF2487	*glf*	-1.9	UDP-galactopyranose mutase	3.40E-03
**Lipid metabolism**					
O994_RS12800	EF0283	*fabF-1*	1.6	3-oxoacyl-ACP synthase	7.90E-03
O994_RS12670	EF0365		3.4	acyl-ACP thioesterase	3.30E-17
O994_RS05090	EF1264		1.5	sulfatase	2.90E-03
O994_RS05585	EF1360	*dhaK*	1.6	dihydroxyacetone kinase	7.40E-02
O994_RS06665	EF1570		1.6	DegV family protein	1.00E-02
O994_RS07510	EF1747	*gpsA*	3.2	NAD(P)H-dependent glycerol-3-phosphate dehydrogenase	2.10E-39
O994_RS07815	EF1813		3.4	lipoteichoic acid synthase	1.30E-15
O994_RS08160	EF1907		1.8	enoyl-CoA hydratase	6.50E-08
O994_RS10320	EF2494	*cdsA*	2.9	phosphatidate cytidylyltransferase	1.50E-19
O994_RS11690	EF2880	*fabF-2*	1.5	3-oxoacyl-ACP synthase	1.60E-03
O994_RS11695	EF2881	*fabG*	1.8	3-oxoacyl-[acyl-carrier-protein] reductase	1.40E-08
O994_RS11700	EF2882	*fabD*	1.4	ACP S-malonyltransferase	1.30E-02
O994_RS01770	EF0521		-2.0	choloylglycine hydrolase	1.10E-06
O994_RS06840	EF1608	*cls*	-1.8	cardiolipin synthetase	1.50E-03
**Membrane transport**					
O994_RS07555	EF1756	*pstB*	1.7	phosphate ABC transporter ATP-binding protein	8.90E-04
O994_RS13970	EF0032		1.7	putative copper transporter	5.80E-05
O994_RS13375	EF0176		1.4	hypothetical protein	3.10E-02
O994_RS13100	EF0238	*cbiO*	1.6	cobalt transporter ATP-binding protein	2.30E-02
O994_RS13090	EF0239		1.5	cobalt transport family protein	3.50E-02
O994_RS03250	EF0907		1.6	peptide ABC transporter peptide-binding protein	3.30E-03
O994_RS03275	EF0912		1.6	peptide ABC transporter ATP-binding protein	3.80E-03
O994_RS03420	EF0941		1.9	ABC transporter ATP-binding protein	2.30E-03
O994_RS03425	EF0942		1.6	ABC transporter ATP-binding protein/permease	9.20E-02
O994_RS04770	EF1198		3.2	ABC transporter permease	2.60E-28

O994_RS04775	EF1199		3.2	phosphate ABC transporter: substrate-binding protein	1.10E-27
O994_RS05035	EF1253		1.8	ABC transporter substrate-binding protein	3.90E-02
O994_RS05085	EF1263		2.3	ftsX-like permease	1.20E-07
O994_RS06690	EF1575		1.4	ABC transporter ATP-binding protein	9.30E-02
O994_RS07295	EF1705		2.7	phosphate-binding protein	9.00E-21
O994_RS07580	EF1761	*ftsE*	1.5	cell division ATP-binding protein	1.50E-02
O994_RS07820	EF1814		3.6	MFS transporter	4.60E-13
O994_RS07480	EF2074		4.4	ABC transporter ATP-binding protein	5.30E-17
O994_RS09855	EF2394	*sufC*	1.4	ABC transporter ATP-binding protein	4.60E-02
O994_RS10825	EF2639		1.6	ABC transporter ATP-binding protein	4.60E-03
O994_RS01460	EF2985		1.5	ABC transporter permease	2.80E-02
O994_RS01455	EF2986		2.1	ABC transporter ATP-binding protein	4.80E-08
O994_RS01450	EF2987		2.4	HlyD family efflux transporter periplasmic adaptor subunit	9.80E-11
O994_RS01150	EF3041		2.2	peptide ABC transporter substrate-binding protein	1.30E-08
O994_RS00870	EF3108	*opp2B*	3.7	oligopeptide ABC transporter permease	2.20E-35
O994_RS00865	EF3109	*opp2F*	4.1	oligopeptide ABC transporter ATP-binding protein	2.40E-33
O994_RS12740	EF0295		-2.1	V-type ATPase subunit J	1.70E-07
O994_RS12735	EF0296	*napA*	-2.3	Na^+/H^+ antiporter	1.70E-15
O994_RS01540	EF0420		-1.6	drug resistance transporter EmrB/QacA family protein	5.50E-02
O994_RS02130	EF0674		-1.8	glycine betaine/carnitine/choline ABC transporter ATP-binding protein	9.50E-04
O994_RS02135	EF0675		-1.7	glycine betaine/carnitine/choline ABC transporter ATP-binding protein	5.50E-03
O994_RS02380	EF0717		-3.3	PTS system fructose-specific transporter subunit IIABC	7.90E-03
O994_RS02395	EF0720		-1.9	voltage-gated chloride channel family protein	1.00E-03
O994_RS02485	EF0739		-1.7	nicotinamide mononucleotide transporter	1.00E-03
O994_RS02510	EF0744		-2.3	sodium/dicarboxylate symporter family protein	9.70E-06
O994_RS02705	EF0785		-1.7	drug resistance transporter EmrB/QacA family protein	1.30E-03
O994_RS03085	EF0872		-3.2	KUP/HAK/KT family potassium transporter	3.80E-45
O994_RS03415	EF0940		-1.6	folate family ECF transporter S component	3.80E-03
O994_RS03920	EF1042		-1.9	multidrug efflux MFS transporter	2.50E-05
O994_RS05615	EF1365		-2.0	AI-2E family transporter	3.70E-04

O994_RS06380	EF1516		-2.2	PTS system transporter subunit IIABC	3.80E-05
O994_RS06525	EF1541		-1.6	riboflavin transporter	2.20E-02
O994_RS06835	EF1607		-2.2	PTS glucose transporter subunit IIA	1.10E-05
O994_RS07425	EF1732		-2.3	ABC transporter ATP-binding protein/peptidase	1.30E-08
O994_RS07430	EF1733		-2.1	ABC transporter ATP-binding protein/peptidase	3.00E-07
O994_RS07745	EF1798		-1.7	AI-2E family transporter	4.90E-03
O994_RS13495	EF1878		-4.1	ATP/GTP-binding protein	8.00E-09
O994_RS09390	EF2221		-2.2	ABC transporter substrate-binding protein	7.00E-04
O994_RS09395	EF2222		-2.4	ABC transporter permease	7.70E-05
O994_RS09415	EF2226		-5.0	ABC transporter ATP-binding protein/permease	1.70E-42
O994_RS09420	EF2227		-5.2	ABC transporter ATP-binding protein/permease	1.00E-39
O994_RS10190	EF2463		-2.8	voltage-gated chloride channel family protein	2.00E-08
O994_RS10280	EF2486	*cpsJ*	-1.7	ABC transporter ATP-binding protein	2.90E-02
O994_RS10395	EF2509		-1.7	AzlC family ABC transporter permease	2.30E-02
O994_RS10835	EF2641		-2.1	glycine betaine/L-proline ABC transporter ATP-binding protein	4.10E-03
O994_RS10840	EF2642		-2.0	glycine betaine/L-proline-binding permease	3.80E-03
O994_RS10875	EF2649		-4.3	spermidine/putrescine ABC transporter	2.10E-44
O994_RS10880	EF2650		-4.5	spermidine/putrescine ABC transporter permease	6.30E-58
O994_RS10885	EF2651		-4.4	spermidine/putrescine ABC transporter permease	1.20E-44
O994_RS10890	EF2652		-4.2	spermidine/putrescine ABC transporter ATP-binding protein	4.30E-46
O994_RS10960	EF2668	*mgtE*	-1.7	magnesium transporter	1.70E-04
O994_RS11015	EF2680		-2.5	ABC transporter ATP-binding protein/permease	4.30E-16
O994_RS11430	EF2768		-3.6	hypothetical protein	2.50E-09
O994_RS11435	EF2769		-3.2	ABC transporter ATP-binding protein	7.40E-16
O994_RS11440	EF2770		-3.4	energy-coupling factor transport system permease protein	1.00E-15
O994_RS11450	EF2772		-2.2	multidrug efflux MFS transporter	1.40E-06
O994_RS01240	EF3022		-2.4	sodium:dicarboxylate symporter family protein	9.90E-06
O994_RS01090	EF3053	*thiT*	-1.8	energy-coupled thiamine transporter	1.10E-04
**<u>Metabolism of cofactors and vitamins</u>**					
O994_RS12285	EF0446	*menE*	1.6	2-succinylbenzoate-CoA ligase	2.00E-03
O994_RS12080	EF0517		1.8	2-dehydropantoate 2-reductase	2.00E-02

O994_RS02965	EF0848	*acpS*	1.8	holo-ACP synthase	2.90E-04
O994_RS03600	EF0978	*folD*	1.5	bifunctional 5,10-methylene-tetrahydrofolate dehydrogenase	2.90E-02
O994_RS03905	EF1039		2.6	Cof-type HAD-IIB family hydrolase	6.40E-18
O994_RS06970	EF1637	*eutT*	2.0	cobalamin adenosyltransferase	8.00E-02
O994_RS07395	EF1725	*fhs*	2.2	formate--tetrahydrofolate ligase	2.00E-12
O994_RS10130	EF2451	*coaD*	2.9	pantetheine-phosphate adenylyltransferase	7.50E-20
O994_RS08535	EF1989	*hemH*	-1.9	ferrochelatase	1.70E-03
O994_RS08880	EF2056	*ubiA*	-2.2	1,4-dihydroxy-2-naphthoate octaprenyltransferase	2.80E-10
O994_RS11425	EF2767		-3.5	transcriptional regulator	3.60E-09
**Metabolism of other amino acids**					
O994_RS04395	EF1121	*racE*	2.0	glutamate racemase	1.40E-05
O994_RS11290	EF2738		2.4	thioredoxin reductase/glutathione-like protein	7.90E-06
**Metabolism of terpenoids and polyketides**					
O994_RS03220	EF0901	*fni*	2.7	isopentenyl pyrophosphate isomerase	1.40E-11
O994_RS03225	EF0902	*mvaK*	2.9	phosphomevalonate kinase	1.30E-14
O994_RS03230	EF0903	*mvaD*	3.7	mevalonate diphosphate decarboxylase	1.10E-22
O994_RS03235	EF0904	*mvk*	3.1	mevalonate kinase	1.80E-20
O994_RS05600	EF1363	*mvaS*	4.4	hydroxymethylglutaryl-CoA synthase	7.80E-96
O994_RS14705	EF1364	*mvaE*	4.6	acetyl-CoA acetyltransferase/hydroxymethylglutaryl-CoA reductase	5.50E-93
O994_RS10325	EF2495	*uppS*	3.3	UDP-diphosphate synthase	3.60E-39
O994_RS00245	EF3245	*uppP*	5.2	cell-envelope associated acid phosphatase	1.60E-102
**Nucleotide metabolism**					
O994_RS13935	EF0039		1.6	deoxyuridine 5`-triphosphate nucleotidohydrolase	4.10E-03
O994_RS13380	EF0175	*cdd*	1.5	cytidine deaminase	6.70E-02
O994_RS12975	EF0264	*hpt*	1.4	hypoxanthine-guanine phosphoribosyltransferase	6.10E-02
O994_RS03890	EF1036	*ndk*	2.8	nucleoside-diphosphate kinase	1.70E-07
O994_RS06555	EF1547	*cmk*	2.1	cytidylate kinase	3.00E-10
O994_RS09560	EF2361	*purB*	1.5	adenylosuccinate lyase	4.60E-02
O994_RS09565	EF2362	*purK-2*	1.9	5-(carboxyamino)imidazole ribonucleotide synthase	1.10E-04

O994_RS11360	EF2754	*nrdD*	1.9	anaerobic ribonucleoside triphosphate reductase	1.10E-04
O994_RS14060	EF0014	*purA*	-2.3	adenylosuccinate synthetase	1.10E-08
O994_RS04520	EF1147	*pyrG*	-3.5	CTP synthetase	2.50E-24
**Other**					
O994_RS13770	EF0076		1.7	short chain dehydrogenase/reductase family oxidoreductase	1.90E-04
O994_RS12660	EF0367		2.2	haloacid dehalogenase	3.10E-05
O994_RS12535	EF0394	*salB*	1.7	serine protease	3.40E-02
O994_RS01630	EF0706		2.6	ATP-dependent Clp protease ATP-binding subunit	2.00E-13
O994_RS02975	EF0850		2.1	type II toxin-antitoxin system PemK/MazF family toxin	4.00E-06
O994_RS03685	EF0995	*ftsQ*	2.2	cell division protein	1.10E-09
O994_RS03690	EF0996	*ftsA*	1.5	cell division protein	3.20E-02
O994_RS03695	EF0997	*ftsZ*	1.5	cell division protein	4.50E-02
O994_RS03720	EF1002	*divIVA*	2.3	cell division protein	6.10E-14
O994_RS04245	EF1091	*ebpA*	3.8	endocarditis and biofilm-associated pilus tip protein	9.70E-05
O994_RS04570	EF1157		1.8	Sapep family $Mn(2^{+})$-dependent dipeptidase	6.10E-03
O994_RS04795	EF1203	*ruvX*	1.4	Holliday junction resolvase-like protein	7.70E-02
O994_RS04915	EF1227		2.1	NADPH-dependent oxidoreductase	6.00E-03
O994_RS05190	EF1284		2.0	putative phage structural protein	1.70E-03
O994_RS05195	EF1285		1.9	phage major tail protein	1.70E-02
O994_RS05275	EF1301		2.7	cell cycle protein	1.50E-07
O994_RS05645	EF1371		1.4	metallo-β-lactamase	6.40E-02
O994_RS06080	EF1455	*terL*	1.9	phage terminase large subunit	8.20E-03
O994_RS06505	EF1537	*xerD*	1.7	site-specific tyrosine recombinase	5.00E-03
O994_RS06570	EF1550	*hup*	1.5	DNA-binding protein	5.10E-02
O994_RS06745	EF1586	*nox*	2.5	NADH oxidase	6.60E-12
O994_RS07205	EF1685	*hlyIII*	1.9	hemolysin III: type II toxin	1.40E-07
O994_RS09370	EF2217		2.6	glycoside hydrolase family 92 protein	1.30E-15
O994_RS10160	EF2457		2.0	FtsW/RodA/SpoVE family cell cycle protein	2.00E-06
O994_RS10360	EF2502		4.5	putative cell division protein	1.00E-58
O994_RS10610	EF2590		3.3	serine hydrolase: β-lactamase transpeptidase family	5.40E-23

O994_RS01165	EF3037	*pepA*	1.7	glutamyl-aminopeptidase	4.90E-03
O994_RS00885	EF3097	*rnc*	2.4	ribonuclease III	8.20E-10
O994_RS13715	EF0090		-1.8	diacylglycerol kinase catalytic subunit	1.50E-05
O994_RS12785	EF0286		-2.8	fibronectin-binding protein	1.40E-09
O994_RS12125	EF0479		-1.6	phage integrase family site specific recombinase	4.70E-03
O994_RS02445	EF0730		-2.6	thioredoxin family protein	1.10E-04
O994_RS02490	EF0740		-1.5	deoxynucleoside kinase	7.20E-02
O994_RS02635	EF0771	*clpP*	-1.9	ATP-dependent Clp protease proteolytic subunit	3.20E-07
O994_RS04290	EF1099		-2.5	LPXTG cell wall anchor domain-containing protein	4.60E-09
O994_RS05710	EF1384	*mprF*	-2.2	Phosphatidylglycerol lysyltransferase	2.90E-05
O994_RS06755	EF1590		-2.6	protease synthase and sporulation negative regulatory protein	7.90E-06
O994_RS08675	EF2015		-1.6	minor head protein	9.60E-02
O994_RS10215	EF2472	*gcp*	-1.6	*O*-sialoglycoprotein endopeptidase	1.40E-02
O994_RS11800	EF2899		-2.8	pyridine nucleotide-disulfide family oxidoreductase	4.00E-08
O994_RS14305	EF3296	*ychF*	-1.9	GTP-dependent nucleic acid-binding protein	7.60E-07
**Poorly characterised**					
O994_RS13925	EF0041		1.6	PilT domain-containing protein	9.20E-04
O994_RS12705	EF0302		1.9	C1 family peptidase	1.00E-07
O994_RS12665	EF0366		3.1	DUF72 domain-containing protein	7.10E-15
O994_RS12255	EF0452		2.5	acyl-CoA synthetase	5.40E-06
O994_RS02165	EF0681		2.0	YlbF family regulator	2.40E-05
O994_RS02175	EF0683		1.4	AAA family ATPase	7.50E-02
O994_RS02180	EF0684		2.4	HD domain-containing protein	1.50E-13
O994_RS02340	EF0708		3.5	hypothetical protein	2.10E-32
O994_RS02525	EF0747		1.9	DUF1003 domain-containing protein	1.00E-05
O994_RS02695	EF0783		3.2	acetyltransferase	5.30E-33
O994_RS02785	EF0802		8.8	DUF3955 domain-containing protein	1.30E-110
O994_RS02820	EF0819		3.2	DUF960 domain-containing protein	1.30E-24
O994_RS03575	EF0972		4.6	metallophosphoesterase	5.40E-66
O994_RS03660	EF0990	*ftsL*	3.7	cell division protein	5.20E-39

O994_RS03740	EF1006		3.7	DUF4828 domain-containing protein	1.20E-50
O994_RS03750	EF1008		1.5	Gfo/Idh/MocA family oxidoreductase	7.50E-02
O994_RS04250	EF1092	*ebpB*	2.8	endocarditis and biofilm-associated pilus minor subunit	6.30E-02
O994_RS04450	EF1132		4.9	CBS-domain containing hypothetical protein	9.90E-76
O994_RS05050	EF1200		2.3	threonine/serine exporter family protein	7.30E-04
O994_RS04800	EF1204		1.5	DUF1292 domain-containing protein	4.10E-03
O994_RS04910	EF1226		2.9	NAD(P)H-dependent oxidoreductase	6.40E-07
O994_RS04935	EF1231		6.3	metallophosphoesterase	2.60E-107
O994_RS05020	EF1249	*efba*	1.7	fibronectin/fibrinogen-binding protein	2.30E-03
O994_RS05060	EF1258		4.7	hypothetical protein	1.80E-52
O994_RS05080	EF1262		2.7	hypothetical protein	3.50E-12
O994_RS05465	EF1327		4.4	2-hydroxyacyl-CoA dehydratase	1.20E-34
O994_RS06085	EF1456		2.5	DUF1073 domain-containing protein	1.70E-07
O994_RS06110	EF1461		2.8	DUF2213 domain-containing protein	3.40E-13
O994_RS06115	EF1462		2.5	hypothetical protein	8.40E-07
O994_RS06120	EF1463		2.2	DUF2184 domain-containing protein	1.90E-04
O994_RS06150	EF1469		1.6	DUF3383 family protein	3.70E-02
O994_RS06440	EF1523		4.2	hypothetical protein	1.30E-66
O994_RS06575	EF1552		2.2	hypothetical protein	4.70E-08
O994_RS06580	EF1553		1.9	tetratricopeptide repeat protein	3.20E-06
O994_RS06670	EF1571		2.6	hypothetical protein	3.10E-11
O994_RS06675	EF1572		2.2	hypothetical protein	8.20E-09
O994_RS06715	EF1580		2.6	HAD superfamily hydrolase	8.30E-12
O994_RS06720	EF1581	*tkt*	2.0	transketolase	5.30E-06
O994_RS06850	EF1610		2.4	DUF1803 domain-containing protein	4.00E-09
O994_RS08155	EF1906		1.8	hypothetical integral membrane protein	2.80E-08
O994_RS08170	EF1909		2.1	hypothetical protein	5.80E-10
O994_RS09045	EF2149		1.6	hypothetical protein	7.20E-04
O994_RS09075	EF2155		2.2	phosphoglucosamine mutase	1.60E-11
O994_RS09080	EF2156		3.5	hypothetical protein	7.50E-48
O994_RS09360	EF2215		4.3	hypothetical protein	1.10E-52

O994_RS09365	EF2216		2.0	FUSC family protein	8.80E-04
O994_RS09620	EF2373		1.6	DUF5590 domain-containg protein	4.10E-03
O994_RS09850	EF2393	*sufD*	1.7	Fe-S cluster assembly protein	2.30E-03
O994_RS10035	EF2433		1.9	phosphoglycerate mutase family protein	2.00E-06
O994_RS10125	EF2450		2.4	PDZ domain-containing protein	2.70E-11
O994_RS10165	EF2458		2.5	YlaN family protein	2.80E-09
O994_RS10205	EF2470		4.1	putative metal-dependent phosphohydrolase	1.00E-54
O994_RS10240	EF2477		1.9	SPFH domain-containing protein	9.30E-04
O994_RS10245	EF2478		1.7	hypothetical protein	3.20E-03
O994_RS11270	EF2734		3.8	Gfo/Idh/MocA family oxidoreductase	6.90E-58
O994_RS11505	EF2784		2.9	DUF3042 domain containing protein	6.60E-16
O994_RS11605	EF2862		5.5	hypothetical protein	1.60E-76
O994_RS11625	EF2867	*tmcAL*	1.4	nucleotidyltransferase	6.10E-02
O994_RS11790	EF2896		6.1	DUF3955 domain-containing protein	3.30E-31
O994_RS11880	EF2915	*mltG*	2.9	mltG-like protein	2.10E-32
O994_RS11945	EF2929		1.7	hypothetical protein	5.30E-03
O994_RS11950	EF2930		1.8	conserved integral membrane protein	3.40E-03
O994_RS01170	EF3036		1.7	thioredoxin family protein	7.80E-04
O994_RS00990	EF3074		1.6	WxL domain-containing protein	8.30E-02
O994_RS00895	EF3095		2.6	Cof-type HAD-IIB family hydrolase	5.00E-13
O994_RS00655	EF3149		2.1	helix-turn-helix domain-containing protein	3.50E-10
O994_RS00650	EF3150		4.3	M16 family peptidase	2.30E-71
O994_RS00645	EF3151		4.2	hypothetical protein	1.60E-58
O994_RS00520	EF3179		1.6	DUF3298 domain-containing protein	6.20E-02
O994_RS00475	EF3188		1.7	hypothetical protein	6.70E-04
O994_RS00305	EF3234		2.5	DUF5105 domain-containing protein	1.00E-13
O994_RS14110	EF0003		-2.3	RNA binding protein	4.00E-12
O994_RS13710	EF0091		-1.7	helix-turn-helix domain-containing protein	6.10E-05
O994_RS12745	EF0294		-1.4	dihydrofolate reductase	7.20E-02
O994_RS12130	EF0477		-1.9	hypothetical protein	4.60E-02

O994_RS01945	EF0638		-2.8	DUF1307 domain-containing protein	1.10E-12
O994_RS02215	EF0690		-2.3	phosphotransferase family protein	1.80E-07
O994_RS02215	EF0690		-2.3	phosphotransferase family protein	1.80E-07
O994_RS03305	EF0918		-2.1	DUF1304 domain-containing protein	2.90E-04
O994_RS03560	EF0969		-1.8	ribosomal-processing cysteine protease	2.90E-06
O994_RS03570	EF0971	*psiE*	-1.6	phosphate-starvation-inducible protein	5.70E-02
O994_RS03945	EF1047		-1.5	DUF177 domain-containing protein	2.00E-02
O994_RS04205	EF1082		-1.5	hypothetical protein	8.50E-02
O994_RS04235	EF1089		-2.3	DUF805 domain-containing protein	9.90E-08
O994_RS04305	EF1102		-1.8	YxeA family protein	1.60E-03
O994_RS04510	EF1145		-1.8	DUF1934 domain-containing protein	2.00E-04
O994_RS04605	EF1164		-2.0	HD domain-containing protein	3.30E-04
O994_RS05010	EF1247		-2.6	iron-sulfur cluster biosynthesis family protein	1.60E-09
O994_RS05015	EF1248		-3.2	hypothetical protein	1.80E-35
O994_RS05260	EF1298		-1.9	DUF1048 domain-containing protein	6.20E-06
O994_RS05620	EF1366		-1.8	hypothetical protein	2.50E-03
O994_RS05670	EF1376		-2.0	VanZ family protein	4.90E-04
O994_RS05705	EF1383		-1.9	DUF1294 domain-containing protein	1.70E-03
O994_RS05920	EF1425		-2.2	helix-turn-helix domain-containing protein	7.60E-03
O994_RS05925	EF1427		-2.3	hypothetical protein	7.30E-03
O994_RS05930	EF1428		-2.5	hypothetical protein	2.60E-03
O994_RS05940	EF1430		-1.8	AAA family ATPase	3.10E-02
O994_RS06845	EF1609		-3.4	DUF2179 domain-containing protein	2.60E-12
O994_RS07210	EF1686		-1.8	hypothetical protein	6.60E-02
O994_RS07435	EF1734		-1.8	YneF family protein	8.90E-04
O994_RS07635	EF1774		-2.3	QueT transporter family protein	1.60E-07
O994_RS08115	EF1897		-4.8	hypothetical protein	4.90E-16
O994_RS08490	EF1980		-3.2	hypothetical protein	1.20E-14
O994_RS08925	EF2065		-1.5	DUF1033 family protein	5.10E-02
O994_RS10005	EF2427		-2.3	hypothetical protein	8.00E-12
O994_RS10195	EF2467		-3.1	hypothetical protein	9.30E-17

O994_RS10400	EF2511		-1.7	AzlD domain-containing protein	8.60E-02
O994_RS10415	EF2548		-1.7	hypothetical protein	2.90E-02
O994_RS10925	EF2659		-2.5	QueT transporter family protein	9.50E-09
O994_RS10930	EF2661		-2.0	diacylglycerol kinase catalytic subunit	1.20E-04
O994_RS10980	EF2672		-1.7	CYTH domain-containing protein	4.20E-03
O994_RS10985	EF2673		-1.5	DsbA family protein	5.00E-02
O994_RS00225	EF2692		-1.7	WxL domain-containing protein	1.40E-03
O994_RS11775	EF2893		-1.8	hypothetical protein	1.40E-04
O994_RS01260	EF3018		-1.8	hypothetical protein	6.40E-05
O994_RS00670	EF3146		-2.5	hypothetical protein	3.60E-08
O994_RS00240	EF3247		-3.4	putative holin-like toxin	3.00E-15
O994_RS00235	EF3249		-3.2	putative holin-like toxin	3.90E-08
O994_RS14315	EF3294		-3.3	hypothetical protein	7.20E-27
O994_RS14310	EF3295		-2.1	DUF1129 domain-containing protein	6.90E-10
O994_RS14300	EF3297		-1.8	DUF951 domain-containing protein	2.40E-04
O994_RS14220	EF3314		-3.0	cell wall surface anchor family protein	5.90E-11
**Replication and repair**					
O994_RS13930	EF0040	*radA*	1.8	DNA repair protein	8.10E-06
O994_RS03630	EF0984	*recN*	1.7	DNA repair protein	1.60E-03
O994_RS05810	EF1404	*mutS2*	2.4	MutS2 family protein	1.30E-22
O994_RS06750	EF1587	*mutT*	4.4	DNA mismatch repair protein	6.10E-48
O994_RS07020	EF1648	*xerC*	2.5	tyrosine recombinase: DNA repair and recombination	1.60E-14
O994_RS11060	EF2689	*sbcC*	1.6	exonuclease	1.30E-02
O994_RS11065	EF2690	*sbcD*	1.9	exonuclease	2.50E-03
O994_RS00890	EF3096	*smc*	3.4	chromosome segregation protein	4.70E-31
O994_RS00765	EF3125	*priA*	2.2	primosomal protein	6.50E-08
O994_RS00585	EF3166	*mutL*	1.5	DNA mismatch repair protein	6.90E-02
O994_RS14085	EF0008	*ssb-1*	-1.9	single-strand binding protein	9.80E-07
O994_RS14105	EF0004	*recF*	-1.7	recombination protein F	3.90E-04
O994_RS04595	EF1162		-2.5	helicase	7.40E-22

**Signal transduction**					
O994_RS05070	EF1260	*yclR*	2.7	metal-induced stress response regulator	4.70E-24
O994_RS05075	EF1261	*yclK*	2.8	metal-induced sensor kinase	3.40E-23
O994_RS01215	EF3027	*htrA*	1.7	serine protease	4.00E-04
O994_RS01065	EF3058		4.4	phosphotyrosine protein phosphatase	2.30E-71
O994_RS00790	EF3120	*ireK*	2.1	serine/threonine protein kinase IreK	8.70E-10
O994_RS00450	EF3194	*lrgA*	2.0	negative regulator of murein hydrolase activity	3.50E-02
O994_RS14335	EF3289	*croR*	7.2	ompR/phoB type DNA-binding response regulator	5.10E-148
O994_RS14330	EF3290	*croS*	7.7	sensor kinase	6.00E-164
O994_RS08900	EF2060	*cydB*	-1.6	cytochrome d ubiquinol oxidase subunit II	3.60E-04
O994_RS08905	EF2061	*cydA*	-1.8	cytochrome d ubiquinol oxidase subunit I	4.00E-07
O994_RS09375	EF2218		-2.8	AraC family transcriptional regulator	3.70E-13
O994_RS09380	EF2219	*yesM*	-2.6	sensor histidine kinase	4.10E-13
O994_RS14215	EF3315	*citG*	-1.6	triphosphoribosyl-dephospho-CoA synthase	7.70E-03
**Transcriptional regulators**					
O994_RS12190	EF0465		2.0	LytR family transcriptional regulator	7.90E-09
O994_RS02225	EF0692		2.1	RpiR family phosphosugar-binding transcriptional regulator	1.30E-03
O994_RS03125	EF0881		1.8	transcriptional repressor NrdR	4.60E-05
O994_RS03210	EF0899		1.8	post-transcriptional regulator	5.90E-03
O994_RS03625	EF0983		2.1	ArgR family transcriptional regulator	1.80E-05
O994_RS03650	EF0988	*mraZ*	3.8	division/cell wall cluster transcriptional repressor	5.30E-36
O994_RS04565	EF1156		2.3	GntR family transcriptional regulator	1.30E-05
O994_RS04835	EF1212		4.5	LytR family transcriptional regulator	2.90E-71
O994_RS05280	EF1302		4.9	transcriptional regulator	1.30E-40
O994_RS05285	EF1303		5.6	LysR family transcriptional regulator	1.70E-53
O994_RS05460	EF1326		1.9	TetR family transcriptional regulator	8.50E-03
O994_RS06660	EF1569	*psr*	3.5	putative transcriptional regulator psr	7.90E-30
O994_RS07005	EF1645	*codY*	2.8	GTP-binding transcriptional repressor	9.10E-27
O994_RS09985	EF2423		2.2	winged helix-turn-helix transcriptional regulator	7.90E-11
O994_RS11140	EF2703		5.4	LytR family transcriptional regulator	7.80E-30

O994_RS11965	EF2933	*rex*	1.6	redox-sensing transcriptional repressor	8.20E-02
O994_RS01385	EF3002	*sylA*	2.0	transcriptional regulator	3.00E-04
O994_RS01060	EF3059		4.7	TetR/AcrR family transcriptional regulator	4.90E-60
O994_RS12400	EF0421		-2.1	MerR family transcriptional regulator	5.40E-03
O994_RS12395	EF0422		-2.5	IclR family transcriptional regulator	6.90E-10
O994_RS02390	EF0719		-3.4	DeoR family transcriptional regulator	2.30E-13
O994_RS03070	EF0869		-2.2	Cro/CI family transcriptional regulator	2.80E-05
O994_RS14630	EF0966		-1.9	MerR family transcriptional regulator	3.00E-04
O994_RS05255	EF1297		-1.9	PadR family transcriptional regulator	2.70E-05
O994_RS05300	EF1306	*hrcA*	-1.8	heat-inducible transcription repressor	4.00E-03
O994_RS05635	EF1369		-2.0	Cro/CI family transcriptional regulator	4.10E-03
O994_RS05905	EF1423		-2.1	Cro/CI family transcriptional regulator	1.00E-03
O994_RS06375	EF1515		-2.2	BlgG family transcription antiterminator	1.40E-03
O994_RS07265	EF1699		-1.9	MerR family transcriptional regulator	1.40E-03
O994_RS08415	EF1965		-1.9	SorC family transcriptional regulator	8.20E-09
O994_RS09410	EF2225		-2.0	MerR family transcriptional regulator	3.10E-04
O994_RS10010	EF2428		-2.4	PadR family transcriptional regulator	8.00E-12
O994_RS10200	EF2469		-2.0	Cro/CI family transcriptional regulator	1.40E-05
O994_RS10820	EF2638	*rex*	-2.2	redox-sensing transcriptional repressor	2.30E-11
O994_RS10895	EF2653		-4.1	Cro/CI family transcriptional regulator	1.20E-42
O994_RS00275	EF3261		-2.0	AbrB family transcriptional regulator	2.10E-08
O994_RS00280	EF3262		-1.9	AbrB family transcriptional regulator	1.40E-07
O994_RS14150	EF3328	*citO*	-1.6	GntR family transcriptional regulator	8.30E-02
**Translation**					
O994_RS12980	EF0263	*tilS*	1.8	tRNA(Ile)-lysidine synthase	4.0E-06
O994_RS02825	EF0820	*ryIY*	1.6	50S ribosomal protein L25	1.6E-03
O994_RS03465	EF0950		1.9	tRNA (adenosine(37)-*N*(6)-threonylcarbamoyltransferase complex	1.0E-03
O994_RS03655	EF0989	*rsmH*	4.0	16S rRNA (cytosine(1402)-*N*(4))-methyltransferase	2.6E-56
O994_RS03725	EF1003	*ileS*	1.8	isoleucine-tRNA ligase	3.50E-04
O994_RS06445	EF1524		2.3	S1 RNA-binding protein	1.4E-09

O994_RS07585	EF1762	*prfB*	1.7	peptide chain release factor 2	9.2E-04
O994_RS08445	EF1971	*hisS*	1.5	histidyl-tRNA synthetase	9.3E-02
O994_RS10135	EF2452	*rsmD*	2.8	16S rRNA (guanine(966)-*N(*2))-methyltransferase	4.0E-19
O994_RS00780	EF3122	*sun*	1.5	16S rRNA (cytosine(967)-*C*(5))-methyltransferase	5.5E-02
O994_RS00770	EF3123	*fmt*	1.8	methionyl-tRNA formyltransferase	2.0E-03
O994_RS00515	EF3180		1.8	σ-70 family RNA polymerase σ factor	2.8E-02
O994_RS14090	EF0007	*rpsF*	-1.7	30S ribosomal protein S6	6.0E-05
O994_RS14080	EF0009	*rpsR*	-1.7	30S ribosomal protein S18	3.7E-05
O994_RS02220	EF0691	*trmB*	-2.3	tRNA (guanine-*N*(7)-)-methyltransferase	2.6E-05
O994_RS02830	EF0821		-1.7	16S rRNA pseudouridine(516) synthase	3.3E-03
O994_RS03285	EF0914	*infC*	-3.4	translation initiation factor IF-3	1.0E-29
O994_RS03290	EF0915	*rpmI*	-3.3	50S ribosomal protein L35	4.8E-30
O994_RS03295	EF0916	*rplT*	-3.1	50S ribosomal protein L20	3.9E-32
O994_RS03555	EF0968	*rplU*	-1.8	50S ribosomal protein L21	1.4E-07
O994_RS03565	EF0970	*rpmA*	-1.7	50S ribosomal protein L27	1.0E-03
O994_RS05095	EF1265		-2.3	23S rRNA (cytosine1962-C5)-methyltransferase	1.0E-15
O994_RS08120	EF1898	*rplS*	-1.4	50S ribosomal protein L19	3.0E-02
O994_RS09875	EF2398	*rpsB*	-1.7	30S ribosomal protein S2	2.5E-05
O994_RS10945	EF2665	*trmL*	-2.2	tRNA (cytidine/uridine-2'-*O*-)-methyltransferase	9.7E-11
O994_RS01210	EF3028		-1.5	tRNA-binding domain-containing protein	1.7E-02
O994_RS01030	EF3065	*rpsO*	-2.0	30S ribosomal protein S15	1.1E-06
O994_RS01005	EF3070	*rpsD*	-1.6	30S ribosomal protein S4	1.3E-03
O994_RS00390	EF3207		-2.3	dihydrouridine synthase	2.3E-10
O994_RS14235	EF3311	*gidA*	-2.3	RNA uridine 5-carboxymethylaminomethyl modification protein	8.6E-11
O994_RS14230	EF3312	*trmE*	-2.1	tRNA modification GTPase	1.5E-06
O994_RS14125	EF3333	*rpmH*	-2.0	50S ribosomal protein L34	3.2E-03
**Unclassified genetic information processing**					
O994_RS11005	EF2678	*spxA*	1.6	hypothetical protein	2.2E-02
O994_RS02680	EF0781		-1.6	cold shock domain-contain protein	3.2E-02

O994_RS08550	EF1991	*cspC*	-1.4	cold shock protein	4.9E-02
**Unclassified: metabolism**					
O994_RS13095	EF0237	*ecfA1*	1.9	energy-coupling factor ABC transporter ATP-binding protein	1.4E-04
O994_RS03385	EF0934		1.5	TatD family hydrolase	3.6E-02
O994_RS04830	EF1211	*npr*	1.4	NADH peroxidase	4.4E-02
O994_RS04905	EF1225	*apbE*	3.6	thiamin biosynthesis	2.8E-15
O994_RS05105	EF1268		2.5	ATPase P: cation transporter E1-E2 family	6.0E-23
O994_RS05290	EF1304	*mgtA-2*	2.8	magnesium-translocating P-type ATPase	2.0E-19
O994_RS05370	EF1311		1.8	serine/threonine protein phosphatase	2.1E-04
O994_RS05530	EF1348		1.6	glucan 1,6-α-glucosidase	4.9E-02
O994_RS06860	EF1612	*pflA*	3.3	pyruvate formate lyase-activating protein	5.0E-04
O994_RS07520	EF1749	*hprK*	1.4	HPr kinase/phosphorylase	9.6E-02
O994_RS09085	EF2157		3.5	DisA-like protein: putative cyclic di-AMP synthase	9.7E-41
O994_RS10355	EF2501		4.3	arsenate reductase: oxidative stress	5.1E-57
O994_RS11295	EF2739	*ahpC*	2.6	peroxiredoxin	3.4E-07
O994_RS01390	EF3001		1.7	protease synthase and sporulation negative regulatory protein	1.9E-02
O994_RS00210	EF3255		2.3	FAD:protein FMN transferase	5.0E-06
O994_RS14275	EF3303		4.0	oleate hydratase	1.7E-54
O994_RS05545	EF1352	*mgtA*	-3.6	magnesium-translocating P-type ATPase	1.9E-76
O994_RS02575	EF0758		-3.2	cadmium-translocating P-type ATPase	4.3E-22
O994_RS01740	EF1352	*mgtA*	-2.3	magnesium-translocating P-type ATPase	3.5E-04
O994_RS05790	EF1400		-3.3	cadmium-translocating P-type ATPase	2.0E-21
O994_RS01025	EF3066	*def*	-2.3	peptide deformylase	9.1E-09
**Xenobiotics biodegradation and metabolism**					
O994_RS10615	EF2591		2.6	glyoxalase	9.7E-13
O994_RS02835	EF0822		-1.8	HAD superfamily hydrolase	3.3E-03
**JH2-2 Specific Genes**					
O994_RS03270			2.0	hypothetical protein	1.7E-05
O994_RS04005			1.5	hypothetical protein	8.9E-03

O994_RS04850	1.7	hypothetical protein	1.5E-02
O994_RS05055	2.9	GW domain-containing glycosaminoglycan-binding protein	6.6E-11
O994_RS06245	2.2	hypothetical protein	1.8E-06
O994_RS09570	5.0	hypothetical protein	2.1E-43
O994_RS13820	1.5	ATP-binding cassette domain-containing protein	4.1E-02
O994_RS14560	1.9	hypothetical protein	2.0E-05
O994_RS14570	2.1	hypothetical protein	1.9E-04
O994_RS14595	2.1	GNAT family N-acetyltransferase	7.1E-07
O994_RS14745	2.1	hypothetical protein	4.5E-06
O994_RS14580	-3.7	hypothetical protein	1.1E-14
O994_RS00785	-1.5	23S ribosomal RNA	2.3E-02
O994_RS00820	-1.8	hypothetical protein	5.2E-02
O994_RS01545	-1.4	IS30-like element IS6770 family transposase	4.2E-02
O994_RS01650	-2.4	hypothetical protein	2.8E-09
O994_RS03080	-3.3	hypothetical protein	1.1E-07
O994_RS03970	-2.6	hypothetical protein	1.8E-12
O994_RS05795	-3.3	RusA family crossover junction endodeoxyribonuclease	9.1E-08
O994_RS06415	-2.0	tRNA-Ile	6.2E-03
O994_RS08540	-1.9	hypothetical protein	3.8E-03
O994_RS08720	-3.4	hypothetical protein	1.1E-19
O994_RS09165	-2.0	type IA DNA topoisomerase	7.0E-03
O994_RS12105	-1.5	multicopper oxidase domain-containing protein	2.4E-02
O994_RS12845	-1.5	IS30-like element IS6770 family transposase	2.0E-02
O994_RS14575	-1.9	ABC transporter permease	1.6E-02
O994_RS14615	-1.8	hypothetical protein	6.9E-03
O994_RS14655	-1.6	IS30-like element IS6770 family transposase	7.8E-04
O994_RS14660	-2.9	IS30-like element IS6770 family transposase	1.0E-20
O994_RS14775	-1.6	IS30-like element IS6770 family transposase	5.4E-04

Table S3. Genes differentially expressed in *E. faecalis* wild-type in the presence versus absence of TXB.

JH2-2 Locus Tag	V583 Gene	Gene Name	F/C (log_2)	Gene Function	P_{adj}
**Amino acid metabolism**					
O994_RS13945	EF0037	*proA*	3.5	γ-glutamyl phosphate reductase	1.20E-38
O994_RS13940	EF0038	*proB*	5.8	γ-glutamyl kinase	8.50E-47
O994_RS12655	EF0368		3.0	aspartate kinase	1.20E-10
O994_RS01925	EF0634	*tdc*	4.8	decarboxylase	2.40E-84
O994_RS03895	EF1037	*aspD*	4.1	aspartate 4-decarboxylase	1.40E-52
O994_RS04125	EF1063		3.2	hypothetical protein	4.10E-11
O994_RS04455	EF1133	*dapD*	4.7	2,3,4,5-tetrahydropyridine-2,6-dicarboxylate *N*-acetyltransferase	1.00E-105
O994_RS04460	EF1134		4.9	M20/M25/M40 family peptidase	2.40E-74
O994_RS05390	EF1314	*alaT*	3.4	alanine aminotransferase	5.80E-41
O994_RS06330	EF1504	*lysA*	2.1	diaminopimelate decarboxylase	2.80E-06
O994_RS06890	EF1618	*eutH*	2.6	ethanolamine utilization protein	7.80E-02
O994_RS07420	EF1731	*aroD*	2.0	3-dehydroquinase	3.30E-06
O994_RS07725	EF1793	*ilvE*	3.0	branched-chain amino acid aminotransferase	2.60E-23
O994_RS09355	EF2214		6.2	glyoxylase	1.10E-70
O994_RS09615	EF2372	*aspB*	1.9	aspartate aminotransferase	7.50E-06
O994_RS11210	EF2721	*sdhb-2*	3.8	L-serine dehydratase, iron-sulfur-dependent subunit β	5.10E-35
O994_RS11215	EF2722	*sdha-2*	3.7	iron-sulfur-dependent L-serine dehydratase subunit α	1.90E-26
O994_RS13675	EF0098	*sdhB-1*	-4.8	L-serine dehydratase, iron-sulfur-dependent subunit β	5.10E-70
O994_RS13670	EF0099	*sdhA-1*	-5.1	iron-sulfur-dependent L-serine dehydratase subunit α	1.20E-69
O994_RS13645	EF0104	*arcA*	-5.6	arginine deaminase	2.20E-44
O994_RS13640	EF0105	*argF-1*	-5.1	ornithine carbamoyltransferase	2.60E-35
O994_RS13635	EF0106	*arcC-1*	-4.8	carbamate kinase	3.20E-32
O994_RS06645	EF1566	*aroA*	-1.7	3-phosphoshikimate 1-carboxyvinyltransferase	2.90E-04
O994_RS06650	EF1567	*aroK*	-2.1	shikimate kinase	5.00E-07
O994_RS06655	EF1568		-2.5	prephenate dehydratase	2.10E-08
O994_RS06960	EF1634	*pduU*	-1.8	propanediol utilization protein	4.50E-02

O994_RS06975	EF1638	*pduV*	-1.8	propanediol utilization protein	3.40E-02
O994_RS10505	EF2568		-5.3	class V aminotransferase	1.40E-36
O994_RS00525	EF3178		-1.9	succinyl-diaminopimelate desuccinylase	7.90E-06
**Autolysis**					
O994_RS13030	EF0252		2.5	*N*-acetylmuramoyl-L-alanine amidase	2.70E-13
O994_RS12300	EF0443		8.6	LysM domain-containing protein	3.50E-71
O994_RS06415	EF1518		8.3	soluble lytic murein transglycosylase	1.60E-16
O994_RS06420	EF1518		7.8	soluble lytic murein transglycosylase	1.00E-60
O994_RS06730	EF1583		1.9	*N*-acetylmuramoyl-L-alanine amidase	8.50E-05
O994_RS02775	EF0799		-1.9	autolysin	9.30E-08
O994_RS11610	EF2863		-2.9	endo-*β*-*N*-acetylglucosaminidase	6.70E-13
**Carbohydrate metabolism**					
O994_RS13020	EF0255	*ldh-1*	1.7	L-lactate dehydrogenase	8.40E-05
O994_RS12670	EF0365		1.7	acyl-ACP thioesterase	1.10E-02
O994_RS03355	EF0928		3.7	glucose uptake protein	1.30E-16
O994_RS03730	EF1004	*zwf*	1.8	glucose-6-phosphate 1-dehydrogenase	3.30E-03
O994_RS05845	EF1411		2.2	6-phospho-*β*-glucosidase	2.50E-08
O994_RS06790	EF1597	*katA*	4.2	catalase/peroxidase	5.80E-34
O994_RS07000	EF1644	*lacX*	2.8	aldolase 1 epimerase	3.10E-19
O994_RS08160	EF1907		2.6	enoyl-CoA hydratase	9.90E-26
O994_RS10350	EF2500		2.8	GcvH family protein	1.10E-12
O994_RS00300	EF3235		1.8	gluconate kinase	8.70E-08
O994_RS14030	EF0020	*mptAB*	-2.9	PTS system mannose-specific transporter subunit IIAB	4.50E-11
O994_RS13795	EF0069	*nanE*	-2.9	*N*-acetylmannosamine-6-phosphate 2-epimerase	1.90E-13
O994_RS13605	EF0114		-5.4	glycosyl hydrolase	2.50E-67
O994_RS13385	EF0174	*deoC*	-2.5	2-deoxyribose-5-phosphate aldolase	4.30E-17
O994_RS13345	EF0185	*deoB*	-1.7	phosphopentomutase	3.40E-05
O994_RS12860	EF0271	*arb*	-3.3	6-phospho-*β*-glucosidase	2.60E-13
O994_RS12855	EF0272		-2.5	glycosyl hydrolase	6.00E-05

O994_RS12440	EF0413	*mtlD*	-7.5	mannitol-1-phosphate 5-dehydrogenase	3.60E-14
O994_RS09760	EF0423	*eda-1*	-3.2	keto-hydroxyglutarate-aldolase	9.80E-10
O994_RS12380	EF0425	*kduI-1*	-3.3	5-keto-4-deoxyuronate isomerase	1.60E-10
O994_RS01960	EF0641	*ldh2*	-1.5	L-lactate dehydrogenase	4.30E-02
O994_RS02145	EF0677		-3.7	phosphoglucomutase/phosphomannomutase	1.40E-23
O994_RS03485	EF0955		-1.7	aldose 1-epimerase	2.90E-07
O994_RS03490	EF0956	*pgmB*	-1.5	β-phosphoglucomutase	1.40E-03
O994_RS03495	EF0957		-1.4	maltose phosphorylase	2.20E-02
O994_RS03505	EF0960		-1.6	endonuclease/exonuclease/phosphatase	2.90E-04
O994_RS04145	EF1068	*galM*	-6.1	aldose 1-epimerase	5.70E-69
O994_RS04150	EF1069	*galK*	-5.8	galactokinase	1.20E-62
O994_RS04155	EF1070	*galE-1*	-5.2	UDP-glucose 4-epimerase	6.40E-46
O994_RS04160	EF1071	*galT*	-1.9	galactose-1-phosphate uridylyltransferase	1.80E-04
O994_RS04330	EF1108	*lutA*	-3.5	oxidoreductase	2.70E-52
O994_RS04335	EF1109	*lutB*	-3.5	iron-sulfur cluster binding protein	1.20E-53
O994_RS04340	EF1110	*lutC*	-3.4	lactate utilisation protein C	2.30E-41
O994_RS09520	EF1213	*alsS*	-4.0	acetolactate synthase	2.40E-12
O994_RS01725	EF1243		-2.7	glycosyl hydrolase	4.60E-05
O994_RS05525	EF1347		-1.6	glycosyl hydrolase	7.30E-04
O994_RS05535	EF1349		-2.3	glycosyl hydrolase	1.30E-12
O994_RS05550	EF1353	*pdhA*	-1.6	pyruvate dehydrogenase complex E1 component subunit α	1.10E-02
O994_RS05555	EF1354	*pdhB*	-1.5	pyruvate dehydrogenase complex, E1 component subunit β	2.50E-02
O994_RS05560	EF1355	*aceF*	-1.5	dihydrolipoamide acetyltransferase	3.30E-02
O994_RS06325	EF1503	*fbp*	-2.1	fructose-1,6-bisphosphatase	3.00E-04
O994_RS06810	EF1602		-5.4	glycosyl hydrolase	1.10E-08
O994_RS07090	EF1661	*bkdD*	-4.6	branched-chain α-keto acid dehydrogenase	1.80E-12
O994_RS07780	EF1805		-4.5	glycosyl hydrolase	1.20E-15
O994_RS07785	EF1806		-2.4	hexose kinase	1.70E-05
O994_RS01700	EF1834	*lacB*	-6.5	galactose-6-phosphate isomerase subunit	6.70E-70
O994_RS01695	EF1835	*lacA*	-6.5	galactose-6-phosphate isomerase subunit	2.60E-84
O994_RS08210	EF1918	*pglA*	-1.6	hypothetical protein	1.30E-02

O994_RS08345	EF1950		-4.6	MocD family protein	3.20E-21
O994_RS08350	EF1951		-7.6	phosphosugar-binding protein	7.30E-17
O994_RS08400	EF1962	*tpiA*	-1.7	triosephosphate isomerase	4.00E-04
O994_RS08405	EF1963	*pgk*	-1.5	phosphoglycerate kinase	8.50E-03
O994_RS08410	EF1964	*gap-2*	-1.8	glyceraldehyde-3-phosphate dehydrogenase	1.10E-06
O994_RS09055	EF2151	*glmS*	-3.0	glucosamine--fructose-6-phosphate aminotransferase	5.00E-29
O994_RS10045	EF2435		-2.3	PTS system transporter subunit IIBC	2.20E-05
O994_RS10285	EF2487	*glf*	-4.8	UDP-galactopyranose mutase	5.00E-79
O994_RS10460	EF2559		-5.2	pyruvate flavodoxin/ferredoxin oxidoreductase family protein	3.50E-45
O994_RS12005	EF2961	*rbsK*	-3.8	ribokinase	4.30E-27
O994_RS01365	EF3006		-3.4	glucose-6-phosphate 1-dehydrogenase family protein	2.50E-13
O994_RS01165	EF3037	*pepA*	-2.0	glutamyl aminopeptidase	8.70E-08
O994_RS00720	EF3135	*uxuA*	-4.9	mannonate dehydratase	4.80E-21
O994_RS00690	EF3141		-7.5	D-isomer specific 2-hydroxyacid dehydrogenase family protein	3.40E-10
O994_RS00685	EF3142	*gnd*	-8.7	6-phosphogluconate dehydrogenase	2.40E-13
O994_RS00620	EF3157		-3.8	glycosyl hydrolase	1.00E-29
O994_RS00615	EF3158	*pgmB*	-3.5	β-phosphoglucomutase	6.70E-15
O994_RS00375	EF3210		-8.2	PTS system transporter subunit IIA	2.20E-50
O994_RS14240	EF3310		-8.7	sorbitol-6-phosphate 2-dehydrogenase	3.30E-18
O994_RS14170	EF3324		-6.3	sodium ion-translocating decarboxylase subunit β	6.60E-101
O994_RS14165	EF3325		-6.8	acetyl-CoA carboxylase biotin carboxyl carrier protein subunit	1.90E-90
**Cellular Community**					
O994_RS01930	EF0635		4.6	amino acid permease	1.50E-54
O994_RS01150	EF3041		3.6	pheromone binding protein	2.30E-31
O994_RS00205	EF3256		1.6	pheromone cAD1 lipoprotein	2.60E-03
O994_RS07790	EF1807	*lacD*	-1.7	tagatose 1,6-diphosphate aldolase	9.80E-02
O994_RS01710	EF1807	*lacD-2*	-5.6	tagatose 1,6-diphosphate aldolase	4.80E-17
O994_RS00880	EF3106	*opp2A*	-3.2	oligopeptide ABC transporter substrate-binding protein	2.00E-50
O994_RS00875	EF3107	*opp2C*	-2.8	oligopeptide ABC transporter permease	7.90E-31
**Drug Resistance: antimicrobial**					

O994_RS06490	EF1534		3.3	cyclophilin type peptidyl-prolyl cis-trans isomerase	2.50E-29
O994_RS11105	EF2698	*telA*	3.8	tellurite resistance protein	1.20E-58
O994_RS06320	EF1502		-2.4	β-lactamase	1.50E-05
**Energy Metabolism**					
O994_RS06270	EF1492		3.6	V-type ATPase subunit F	9.20E-51
O994_RS06275	EF1493		3.1	V-type ATP synthase subunit I	2.70E-32
O994_RS06280	EF1494		2.5	V-type ATP synthase subunit K	8.00E-16
O994_RS06285	EF1495		2.4	V-type ATPase subunit E	8.70E-14
O994_RS06290	EF1496		2.2	V-type ATPase subunit C	6.10E-12
O994_RS06295	EF1497		1.9	V-type ATP synthase subunit F	7.20E-07
O994_RS06300	EF1498		1.9	V-type ATP synthase subunit A	9.70E-08
O994_RS06305	EF1499		1.7	V-type ATP synthase subunit B	1.60E-05
O994_RS06310	EF1500		1.7	V-type ATP synthase subunit D	1.90E-04
O994_RS07325	EF1711		2.4	carbonic anhydrase	6.90E-08
O994_RS10465	EF2560	*gltA*	-5.0	oxidoreductase	1.10E-34
O994_RS10685	EF2607	*atpC*	-1.9	F_0F_1 ATP synthase subunit ε	1.10E-06
O994_RS10690	EF2608	*atpD*	-1.8	F_0F_1 ATP synthase subunit β	2.10E-05
O994_RS10695	EF2609	*atpG*	-1.4	F_0F_1 ATP synthase subunit γ	6.70E-02
**Folding,sorting and degradation**					
O994_RS04345	EF1111		2.7	signal peptidase I	2.70E-13
O994_RS06545	EF1545	*recQ-1*	2.8	ATP-dependent DNA helicase	2.90E-17
O994_RS07010	EF1646	*hslU*	3.4	ATP-dependent protease	5.10E-35
O994_RS07015	EF1647	*hslV*	3.9	ATP-dependent protease	1.00E-40
O994_RS07170	EF1678		1.5	signal peptidase I	4.40E-02
O994_RS07270	EF1700	*ffh*	1.6	signal recognition particle protein	7.90E-04
O994_RS07385	EF1723	*lspA*	1.5	lipoprotein signal peptidase	3.50E-02
O994_RS07590	EF1763	*secA*	1.8	preprotein translocase subunit	5.00E-06
O994_RS08950	EF2070	*mnmA*	2.1	tRNA-specific 2-thiouridylase	7.10E-08
O994_RS09650	EF2380	*rseP*	2.5	regulator of σ E protease	1.80E-16

O994_RS11250	EF2730	*secE*	3.5	preprotein translocase subunit	9.40E-12
O994_RS11970	EF2934	*thiI*	1.8	thiazole biosynthesis protein	1.60E-02
O994_RS13155	EF0227	*secY*	-3.5	preprotein translocase subunit	9.70E-28
O994_RS08395	EF1961	*eno*	-1.5	enolase	2.00E-02
O994_RS14135	EF3331		-1.4	pheromone cCF10 percursor/lipoprotein	3.10E-02
O994_RS08395	EF1961	*eno*	-1.5	enolase	2.00E-02
O994_RS14135	EF3331		-1.4	pheromone cCF10 percursor/lipoprotein	3.10E-02
**Glycan biosynthesis and metabolism**					
O994_RS02100	EF0668	*murE*	2.2	UDP-*N*-acetylmuramoylalanyl-D-glutamate--L-lysine ligase	9.10E-08
O994_RS02105	EF0669		3.2	polysaccharide biosynthesis protein	5.70E-28
O994_RS02160	EF0680		5.7	penicillin-binding protein 2A	1.90E-206
O994_RS02520	EF0746	*pbp(6)*	4.9	penicillin binding protein	5.10E-68
O994_RS02760	EF0796		4.2	type 2 phosphatidic acid phosphatase	1.40E-25
O994_RS03665	EF0991	*pbpC*	2.2	penicillin-binding protein C	1.90E-11
O994_RS03845	EF1027	*mprF*	3.4	bifunctional lysylphosphatidylglycerol flippase/synthetase	2.20E-34
O994_RS04525	EF1148	*pbp1A*	3.3	penicillin-binding protein 1A	5.70E-28
O994_RS04630	EF1169	*murAB*	1.7	UDP-*N*-acetylglucosamine 1-carboxyvinyltransferase	4.70E-02
O994_RS05270	EF1300		4.8	putative lipid II flippase	6.10E-21
O994_RS06550	EF1546		2.8	LysM domain-containing protein	6.60E-31
O994_RS07470	EF1740	*pbp1B*	3.1	penicillin-binding protein 1B	3.70E-22
O994_RS08165	EF1908	*murC*	2.0	UDP-*N*-acetylmuramate--L-alanine ligase	6.00E-05
O994_RS08320	EF1945		2.3	putative polyglycerophosphate lipoteichoic acids	7.50E-06
O994_RS09050	EF2150		1.9	FemAB family protein	7.50E-08
O994_RS09150	EF2170	*epaX*	2.3	glycosyl transferase group 2 family protein	2.30E-07
O994_RS09155	EF2171	*epaW*	3.1	dTDP-4-dehydro-6-deoxy-D-glucose	1.20E-15
O994_RS09160	EF2172	*ispD*	4.3	2-C-methyl-D-erythritol 4-phosphate cytidylyltransferase	7.60E-48
O994_RS09245	EF2192	*epaG*	2.3	dTDP-glucose 4,6-dehydratase	8.50E-11
O994_RS09250	EF2193	*epaF*	2.8	dTDP-4-dehydrorhamnose 3,5-epimerase	4.60E-21
O994_RS09255	EF2194	*epaE*	2.9	glucose-1-phosphate thymidylyltransferase	4.80E-27
O994_RS09260	EF2195	*epaD*	3.1	α-1,3-L-rhamnosyltransferase	6.60E-21

O994_RS09265	EF2196	*epaC*	3.4	glycosyl transferase group 2 family protein	7.30E-29
O994_RS09270	EF2197	*epaB*	3.7	α-D-GlcNAc-pyrophosphate polyprenol, α-3-L-rhamnosyl transferase	4.90E-34
O994_RS09275	EF2198	*tagO*	2.4	glycosyl transferase family protein	1.10E-15
O994_RS10065	EF2439	*uppP*	2.0	UDP pyrophosphate phosphatase	1.90E-03
O994_RS10235	EF2476	*pbp4(5)*	2.0	penicillin-binding protein 4	2.20E-08
O994_RS10585	EF2585	*murT*	4.9	mur ligase	7.50E-107
O994_RS10590	EF2586	*gatD*	4.8	glutamine amindotransferase	8.50E-65
O994_RS10675	EF2605	*murAA*	2.2	UDP-*N*-acetylglucosamine 1-carboxyvinyltransferase 1	2.30E-09
O994_RS10780	EF2627		2.3	teichoic acid glycosylation protein	5.50E-04
O994_RS10920	EF2658		4.9	FemAB family protein	5.40E-77
O994_RS11265	EF2733	*murB*	2.4	UDP-*N*-acetylenolpyruvoylglucosamine reductase	3.80E-15
O994_RS11345	EF2750	*dltX*	5.6	teichoic acid D-ala incorporation-associated protein	4.70E-102
O994_RS11580	EF2857	*pbp2B*	3.5	penicillin-binding protein 2B	2.20E-56
O994_RS11595	EF2860	*pbp*	4.6	putative peptidoglycan transpeptidase	2.40E-77
O994_RS11880	EF2915	*mltG*	3.1	mltG-like protein	1.00E-35
O994_RS01055	EF3060	*salA*	2.8	peptidoglycan DL-endopeptidase	2.70E-11
O994_RS07305	EF1707		-1.8	glycosyl hydrolase	3.20E-02
O994_RS10070	EF2440		-4.7	celC-like protein	2.60E-26
O994_RS14400	EF2489	*cspG*	-4.4	MurB family protein	2.00E-63
**Lipid Metabolism**					
O994_RS12805	EF0282	*fabI*	1.5	enoyl-ACP reductase	2.80E-03
O994_RS12800	EF0283	*fabF*	1.5	3-oxoacyl-ACP synthase	4.80E-03
O994_RS05090	EF1264		3.7	sulfatase	9.00E-57
O994_RS06665	EF1570		2.6	DegV family protein	2.00E-11
O994_RS07510	EF1747	*gpsA*	1.7	NAD(P)H-dependent glycerol-3-phosphate dehydrogenase	2.10E-04
O994_RS07815	EF1813		2.9	sulfatase	3.20E-10
O994_RS08150	EF1904		3.0	glycerophosphoryl diester phosphodiesterase family protein	2.40E-13
O994_RS10320	EF2494	*cdsA*	3.0	phosphatidate cytidylyltransferase	3.90E-22
O994_RS11070	EF2691	*plsC*	2.5	1-acyl-sn-glycerol-3-phosphate acyltransferase	1.70E-09
O994_RS11685	EF2879	*accB*	1.4	acetyl-CoA carboxylase, biotin carboxyl carrier protein	2.40E-02

O994_RS11690	EF2880	*fabF-2*	1.6	3-oxoacyl-ACP synthase	5.40E-03
O994_RS11695	EF2881	*fabG*	2.2	3-ketoacyl-ACP reductase	1.60E-08
O994_RS11700	EF2882	*fabD*	2.6	ACP S-malonyltransferase	6.10E-17
O994_RS11705	EF2883	*fabK*	3.3	enoyl-ACP reductase	5.90E-34
O994_RS11710	EF2884	*acpP*	1.9	acyl carrier protein	2.70E-08
O994_RS11715	EF2885	*fabH*	2.3	3-oxoacyl-ACP synthase III	2.00E-16
O994_RS11740	EF2891	*bgsA*	2.1	glycosyl transferase family protein	6.30E-09
O994_RS02430	EF0727		-1.8	lipid kinase	1.10E-06
O994_RS05575	EF1358	*gldA*	-4.6	glycerol dehydrogenase	1.30E-43
O994_RS05580	EF1359	*dhaM*	-4.3	dihydroxyacetone kinase	1.50E-35
O994_RS04675	EF1359		-4.5	hypothetical protein	9.70E-43
O994_RS05585	EF1360	*dhaK*	-4.0	dihydroxyacetone kinase	1.30E-31
O994_RS05590	EF1361	*dhaL*	-4.0	dihydroxyacetone kinase	4.60E-30
O994_RS08255	EF1928	*glpO*	-4.0	α-glycerophosphate oxidase	1.70E-27
O994_RS08260	EF1929	*glpK*	-4.5	glycerol kinase	4.10E-32
O994_RS10860	EF2646		-4.9	glycerate kinase	7.00E-07
O994_RS01370	EF3005		-3.2	choloylglycine hydrolase	7.00E-24
**Membrane transport**					
O994_RS13970	EF0032		2.6	putative copper transporter	1.40E-18
O994_RS13100	EF0238	*cbiO*	1.9	cobalt transporter ATP-binding protein	2.60E-04
O994_RS12425	EF0417		3.2	ABC transporter permease	2.10E-29
O994_RS12420	EF0418		2.3	ABC transporter ATP-binding protein	5.50E-14
O994_RS12290	EF0455		1.6	PTS system transporter subunit IIC	6.70E-03
O994_RS01935	EF0636	*nhaC*	3.9	Na+:H+ antiporter, NhaC family	2.20E-31
O994_RS02265	EF0700		2.2	HlyC/CorC family transporter	9.30E-08
O994_RS02485	EF0739	*pnuC*	3.5	nicotinamide riboside transporter	1.80E-27
O994_RS02590	EF0761		2.6	amino acid ABC transporter amino acid-binding/permease	3.40E-13
O994_RS03025	EF0859		3.2	cation efflux family protein	4.50E-09
O994_RS03085	EF0872		1.8	KUP system potassium uptake protein	7.90E-05
O994_RS04465	EF1135	*mscS*	2.2	moderate conductance mechanosensitive channel	6.00E-08

O994_RS04770	EF1198		5.0	ABC transporter permease	1.40E-41
O994_RS04775	EF1199		4.9	phosphate ABC transporter: substrate-binding protein	7.60E-38
O994_RS05495	EF1341	*macA*	4.0	ABC transporter ATP-binding protein/permease	2.10E-34
O994_RS13465	EF1408		2.7	ABC transporter ATP-binding protein	7.70E-11
O994_RS06525	EF1541		2.2	riboflavin transporter	3.70E-06
O994_RS07140	EF1672		5.3	putative ABC transport system permease protein	3.40E-64
O994_RS07145	EF1673		5.4	putative ABC transport system ATP-binding protein	1.70E-59
O994_RS07560	EF1757	*pstA*	1.6	phosphate ABC transporter permease	1.40E-02
O994_RS07565	EF1758	*pstC*	2.7	phosphate ABC transporter permease	1.70E-13
O994_RS07580	EF1761	*ftsE*	1.7	cell division ATP-binding protein	6.10E-03
O994_RS07820	EF1814		3.9	drug resistance transporter EmrB/QacA family protein	4.70E-12
O994_RS08840	EF2047		3.3	basic amino acid/polyamine antiporter, APA family	3.40E-11
O994_RS08940	EF2068		2.2	multidrug efflux MFS transporter	1.40E-05
O994_RS07480	EF2074		3.6	ABC transporter ATP-binding protein	2.00E-10
O994_RS09215	EF2183		1.9	ABC transporter permease	7.20E-03
O994_RS10060	EF2438		2.8	PTS system transporter subunit IIA	1.10E-17
O994_RS10175	EF2460	*typA*	2.8	GTP-binding protein	7.70E-14
O994_RS10340	EF2498	*metN-2*	2.2	ABC transporter ATP-binding protein	4.00E-06
O994_RS10825	EF2639		2.7	multidrug ABC transporter ATP-binding protein	2.90E-13
O994_RS11205	EF2720		2.0	ABC transporter ATP-binding protein	5.60E-07
O994_RS11855	EF2910	*trk*	1.4	potassium uptake transporter	6.30E-02
O994_RS11975	EF2935		2.6	xanthine/uracil permeases family protein	5.50E-07
O994_RS01460	EF2985		5.2	putative ABC transport system permease protein	2.90E-90
O994_RS01455	EF2986		5.5	putative ABC transport system ATP-binding protein	4.40E-62
O994_RS01450	EF2987		6.3	HlyD family efflux transporter periplasmic adaptor subunit	1.70E-101
O994_RS01280	EF3015	*sstT*	1.9	serine/threonine transporter	7.20E-04
O994_RS01010	EF3069		3.6	formate/nitrite transporter family protein	8.40E-09
O994_RS14035	EF0019	*mptA*	-2.3	PTS system transporter subunit IIB	6.70E-06
O994_RS14025	EF0021	*mptC*	-3.1	PTS system mannose-specific transporter subunit IIC	2.00E-12
O994_RS14020	EF0022	*mptD*	-3.1	PTS system mannose-specific transporter subunit IID	1.20E-12

O994_RS13625	EF0108	*arcD*	-3.7	C4-dicarboxylate transporter	5.90E-15
O994_RS13375	EF0176		-2.5	hypothetical protein	6.40E-17
O994_RS13370	EF0177		-2.7	hypothetical protein	3.40E-28
O994_RS13365	EF0178		-2.9	ABC transporter ATP-binding protein	1.30E-20
O994_RS13360	EF0179		-2.8	ABC transporter permease	7.70E-21
O994_RS13355	EF0180		-2.7	ABC transporter permease	2.40E-19
O994_RS12865	EF0270		-4.3	PTS system β-glucoside-specific transporter subunit IIABC	7.60E-26
O994_RS12575	EF0385		-6.0	major facilitator family transporter	7.60E-13
O994_RS12490	EF0402	*nhaC-1*	-2.2	Na^+/H^+ antiporter	1.10E-04
O994_RS12450	EF0411		-9.1	PTS system mannitol-specific transporter subunit IIBC	4.00E-135
O994_RS12445	EF0412	*mltF*	-8.1	PTS system mannitol-specific transporter subunit IIA	2.90E-17
O994_RS01540	EF0420		-1.7	drug resistance transporter EmrB/QacA family protein	4.70E-02
O994_RS02205	EF0688		-1.4	ABC-2 type transport system ATP-binding protein	7.80E-02
O994_RS02210	EF0689		-1.7	ABC-2 type transport system permease protein	1.40E-03
O994_RS02705	EF0785		-1.8	drug resistance transporter EmrB/QacA family protein	3.40E-04
O994_RS03075	EF0871		-2.4	cation transporter E_1-E_2 family ATPase	4.30E-13
O994_RS03405	EF0938	*ugpC*	-4.8	sugar ABC transporter ATP-binding protein	3.80E-39
O994_RS03500	EF0958		-2.2	PTS system transporter subunit IIABC	1.40E-12
O994_RS03765	EF1012		-5.9	PTS system transporter subunit IIB	1.40E-03
O994_RS03770	EF1013		-6.7	PTS system transporter subunit IIC	7.80E-07
O994_RS03790	EF1017		-3.3	PTS system transporter subunit IIB	9.90E-02
O994_RS03795	EF1018		-3.6	PTS system transporter subunit IIA	6.80E-02
O994_RS01715	EF1018		-4.7	PTS system transporter subunit IIA	3.40E-23
O994_RS03800	EF1019		-3.5	PTS system transporter subunit IIC	9.70E-02
O994_RS04095	EF1057	*mntH2*	-2.3	manganese transport protein	3.50E-06
O994_RS04295	EF1100		-2.3	ABC transporter ATP-binding protein/permease	6.10E-05
O994_RS01720	EF1160	*celB*	-3.4	PTS system cellobiose-specific transporter subunit IIC	2.30E-10
O994_RS04585	EF1160		-3.5	PTS system cellobiose-specific transporter subunit IIC	3.50E-11
O994_RS04940	EF1232		-4.7	ABC transporter permease	7.30E-17
O994_RS04945	EF1233		-4.6	ABC transporter permease	6.90E-15
O994_RS04950	EF1234		-4.2	ABC transporter substrate-binding protein	1.30E-27

O994_RS05835	EF1409		-1.4	ABC transporter permease	7.60E-02
O994_RS06380	EF1516		-2.2	PTS system transporter subunit IIABC	8.70E-07
O994_RS06470	EF1529		-2.6	PTS system transporter subunit IIC	1.80E-07
O994_RS06805	EF1601		-4.6	PTS system transporter subunit IIABC	9.00E-23
O994_RS06980	EF1639		-2.1	iron ABC transporter ATP-binding protein	2.20E-05
O994_RS06985	EF1639	*btuC*	-2.5	iron ABC transporter ATP-binding protein	6.80E-09
O994_RS06990	EF1641		-2.3	iron ABC transporter iron compound-binding protein	2.50E-06
O994_RS07770	EF1803		-3.7	PTS system transporter subunit IIC	1.10E-12
O994_RS09755	EF1836		-3.1	PTS system transporter subunit IIA	3.50E-09
O994_RS09745	EF1838		-2.3	PTS system transporter subunit IIC	4.70E-05
O994_RS08220	EF1920		-5.2	C4-dicarboxylate transporter, DcuC family	2.00E-24
O994_RS08250	EF1927	*glpF*	-3.8	glycerol uptake facilitator protein	6.70E-18
O994_RS08355	EF1952		-6.4	PTS system transporter subunit IID	3.60E-17
O994_RS08360	EF1953		-7.1	PTS system transporter subunit IIC	7.30E-24
O994_RS08890	EF2058	*cydC*	-2.0	transport ATP-binding protein	6.20E-09
O994_RS08895	EF2059	*cydD*	-1.8	transport ATP-binding protein	3.50E-06
O994_RS09350	EF2213		-6.3	PTS system transporter subunit IIBC	3.00E-17
O994_RS09390	EF2221		-6.2	ABC transporter substrate-binding protein	5.80E-65
O994_RS09395	EF2222		-6.7	ABC transporter permease	8.30E-75
O994_RS09400	EF2223		-7.6	ABC transporter permease	1.50E-77
O994_RS10080	EF2442	*PiT*	-5.2	inorganic phosphate transporter, PiT family	2.00E-45
O994_RS10275	EF2485	*cpsK*	-4.4	ABC transporter permease	1.30E-35
O994_RS10280	EF2486	*cpsJ*	-4.5	ABC transporter ATP-binding protein	3.20E-64
O994_RS10665	EF2602		-1.5	PTS glucitol/sorbitol transporter subunit IIA	1.70E-02
O994_RS10865	EF2647	*grtP*	-5.9	gluconate:H^+ symporter, GntP family	4.10E-42
O994_RS10960	EF2668	*mgtE*	-1.5	magnesium transporter	1.70E-02
O994_RS11820	EF2903		-2.4	multiple sugar transport system substrate-binding protein	3.00E-12
O994_RS11830	EF2905		-1.6	multiple sugar transport system permease protein	8.20E-02
O994_RS11995	EF2959	*rbsU*	-2.7	putative ribose uptake protein	8.20E-11
O994_RS12000	EF2960	*rbsD*	-3.5	D-ribose pyranase	6.30E-19
O994_RS12020	EF2964	*ulaA*	-7.3	PTS system ascorbate-specific transporter subunit IIC	2.40E-78

O994_RS12025	EF2965		-8.2	hypothetical protein	9.20E-60
O994_RS09765	EF2986		-4.1	ABC transporter ATP-binding protein	9.40E-27
O994_RS01360	EF3007		-3.2	chloride ion channel protein	4.10E-19
O994_RS01205	EF3029		-4.7	PTS system transporter subunit IID	1.40E-16
O994_RS01200	EF3030		-4.3	PTS system transporter subunit IIC	3.20E-17
O994_RS01195	EF3031		-4.4	PTS system transporter subunit IIB	2.40E-14
O994_RS00870	EF3108		-2.4	oligopeptide ABC transporter permease Opp2B	6.30E-17
O994_RS00865	EF3109		-1.7	oligopeptide ABC transporter ATP-binding protein Opp2F	1.10E-03
O994_RS00715	EF3136		-5.7	PTS system transporter subunit IIA	3.90E-25
O994_RS00710	EF3137		-6.3	PTS sugar transporter subunit IIB	3.30E-10
O994_RS00705	EF3138		-6.5	PTS system transporter subunit IID	4.40E-08
O994_RS00700	EF3139		-6.7	PTS system transporter subunit IIC	3.10E-48
O994_RS00435	EF3198		-2.2	YaeC family lipoprotein	1.90E-03
O994_RS00430	EF3199		-2.1	ABC transporter permease	2.30E-03
O994_RS00370	EF3211		-8.8	PTS system transporter subunit IIB	2.80E-55
O994_RS00365	EF3212		-8.3	PTS system transporter subunit IIC	2.40E-59
O994_RS00360	EF3213		-7.7	PTS system transporter subunit IID	1.40E-52
O994_RS14260	EF3306		-4.3	PTS system sorbitol-specific transporter subunit IIBC	2.70E-14
O994_RS14255	EF3307	*srlA*	-5.1	PTS system sorbitol-specific transporter subunit IIC	5.30E-13
O994_RS14155	EF3327		-9.8	citrate transporter	7.80E-108
**Metabolism of cofactors and vitamins**					
O994_RS12640	EF0371		2.1	class V aminotransferase	8.30E-07
O994_RS02965	EF0848	*acpS*	2.5	holo-ACP synthase	2.70E-13
O994_RS03905	EF1039		3.7	HAD superfamily hydrolase	1.30E-33
O994_RS07395	EF1725	*fhs*	1.7	formate--tetrahydrofolate ligase	1.00E-04
O994_RS08435	EF1969		1.5	phosphomethylpyrimidine kinase	5.00E-02
O994_RS08960	EF2072		1.7	class V aminotransferase	1.90E-03
O994_RS10130	EF2451	*coaD*	1.4	pantetheine-phosphate adenylyltransferase	9.30E-02
O994_RS10770	EF2625	*nadE*	2.1	NAD synthetase	1.50E-08
O994_RS10775	EF2626		2.6	nicotinate phosphoribosyltransferase	1.70E-20
O994_RS00145	EF3269	*folB*	1.8	dihydroneopterin aldolase	3.30E-02

O994_RS13340	EF0186	*deoD-1*	-1.7	purine nucleoside phosphorylase	2.70E-04
O994_RS13025	EF0253		-5.6	aldehyde dehydrogenase	5.40E-67
O994_RS06970	EF1637	*eutT*	-1.8	ATP:cob(I)alamin adenosyltransferase	4.30E-02
O994_RS07920	EF1858	*panD*	-1.4	aspartate α-decarboxylase	8.60E-02
O994_RS07925	EF1859	*panC*	-1.7	pantoate- β-alanine ligase	5.20E-04
O994_RS07930	EF1860	*panB*	-1.6	3-methyl-2-oxobutanoate hydroxymethyltransferase	1.50E-03
O994_RS08535	EF1989	*hemH*	-2.1	ferrochelatase	2.00E-05
O994_RS10510	EF2569		-5.6	hypothetical protein	1.00E-04
**Metabolism of other amino acids**					
O994_RS02970	EF0849	*alr*	2.0	alanine racemase	7.30E-11
O994_RS11340	EF2749	*dltA*	4.5	D-alanine-poly(phosphoribitol) ligase subunit 1	6.60E-88
O994_RS10500	EF2567	*selD*	-5.2	selenide water dikinase	1.60E-41
O994_RS10565	EF2581		-5.7	selenate reductase subunit	2.90E-38
**Metabolism of terpenoids and polyketides**					
O994_RS13875	EF0051	*ipk*	2.1	4-diphosphocytidyl-2-C-methyl-D-erythritol kinase	1.10E-05
O994_RS03225	EF0902	*mvaK*	2.0	phosphomevalonate kinase	2.30E-04
O994_RS03230	EF0903	*mvaD*	2.7	diphosphomevalonate decarboxylase	1.50E-09
O994_RS03235	EF0904	*mvk*	4.3	mevalonate kinase	4.90E-34
O994_RS05600	EF1363	*mvaS*	4.9	hydroxymethylglutaryl-CoA synthase	3.90E-71
O994_RS14705	EF1364	*mvaE*	4.0	acetyl-CoA acetyltransferase/hydroxymethylglutaryl-CoA reductase	3.10E-45
O994_RS10325	EF2495	*uppS*	3.4	UDP-diphosphate synthase	2.50E-36
O994_RS00245	EF3245	*uppP*	5.1	cell-envelope associated acid phosphatase	2.80E-94
**Nucleotide metabolism**					
O994_RS13400	EF0171	*adD*	3.5	adenosine deaminase	1.20E-18
O994_RS12975	EF0264	*hpt*	3.2	hypoxanthine-guanine phosphoribosyltransferase	2.50E-24
O994_RS12790	EF0285	*pyrD-1*	1.7	dihydroorotate dehydrogenase 1A	1.70E-04
O994_RS12155	EF0471	*nrdE*	1.8	ribonucleotide-diphosphate reductase subunit α	3.30E-06
O994_RS12150	EF0472	*nrdI*	2.1	ribonucleotide reductase stimulatory protein	5.70E-09
O994_RS05415	EF1319		1.8	hypothetical protein	3.70E-03

O994_RS06555	EF1547	*cmk*	2.3	cytidylate kinase	1.70E-13
O994_RS09560	EF2361	*purB*	2.3	adenylosuccinate lyase	8.80E-13
O994_RS09565	EF2362	*purK*	3.0	5-(carboxyamino)imidazole ribonucleotide synthase	2.20E-22
O994_RS09575	EF2364		2.0	xanthine permease	2.20E-03
O994_RS10030	EF2432		1.9	metallo-β-lactamase superfamily protein	7.30E-07
O994_RS10115	EF2448		2.3	comE operon protein 2	9.40E-05
O994_RS10445	EF2555	*tdk*	2.1	thymidine kinase	2.10E-06
O994_RS11360	EF2754	*nrdD*	1.6	anaerobic ribonucleoside triphosphate reductase	9.40E-04
O994_RS00755	EF3127	*gmk*	3.0	guanylate kinase	4.60E-22
O994_RS14320	EF3293	*guaB*	2.8	inosine 5'-monophosphate dehydrogenase	9.90E-18
O994_RS13845	EF0062		-2.5	5'-nucleotidase	2.00E-11
O994_RS13390	EF0173	*pyn*	-2.1	pyrimidine-nucleoside phosphorylase	4.50E-10
O994_RS13380	EF0175	*cdd*	-2.4	cytidine deaminase	3.30E-15
O994_RS13335	EF0187	*deoD-2*	-1.8	purine nucleoside phosphorylase	7.50E-05
O994_RS13150	EF0228	*adk*	-3.7	adenylate kinase	2.60E-37
O994_RS12570	EF0386	*arcC-2*	-3.2	carbamate kinase	1.10E-06
O994_RS07345	EF1715	*pyrK*	-1.7	dihydroorotate dehydrogenase electron transfer subunit	4.90E-02
O994_RS07350	EF1716	*carB*	-1.6	carbamoyl phosphate synthase large subunit	1.70E-02
O994_RS09865	EF2396	*pyrH*	-2.4	uridylate kinase	1.60E-09
O994_RS10595	EF2587		-1.6	inosine-uridine preferring nucleoside hydrolase	8.20E-03
O994_RS11815	EF2902		-2.3	2',3'-cyclic-nucleotide 2'-phosphodiesterase	1.50E-08
O994_RS14205	EF3317	*citM*	-4.6	oxaloacetate decarboxylase	6.50E-49
**Other**					
O994_RS12970	EF0265	*ftsH*	1.5	cell division protease	9.50E-03
O994_RS12965	EF0266	*hsp33*	4.5	molecular chaperone	3.00E-29
O994_RS02490	EF0740		2.3	deoxynucleoside kinase	2.30E-04
O994_RS02750	EF0794		3.4	rRNA methyltransferase	1.80E-23
O994_RS03660	EF0990	*ftsL*	2.4	cell division protein	1.00E-14
O994_RS03720	EF1002	*divIVA*	1.6	cell division protein	3.80E-04
O994_RS04540	EF1151	*gpsB*	1.4	cell division protein	7.10E-02

O994_RS05480	EF1338	*trxB*	1.4	thioredoxin reductase	8.50E-02
O994_RS05645	EF1371		2.4	metallo-β-lactamase	3.00E-10
O994_RS06745	EF1586	*nox*	2.5	NADH oxidase	5.50E-21
O994_RS07105	EF1665	*traX*	9.1	conjugal transfer protein	7.90E-11
O994_RS07175	EF1679		1.6	carboxyl-terminal processing protease	4.10E-03
O994_RS07205	EF1685	*hlyIII*	3.2	hemolysin III	2.30E-32
O994_RS08205	EF1917	*clpX*	2.1	Clp protease ATP-binding subunit	5.30E-11
O994_RS09290	EF2201		2.0	flavodoxin	1.60E-04
O994_RS09535	EF2352	*lepA*	1.4	GTP-binding protein	7.10E-02
O994_RS09550	EF2355	*clpB*	2.2	Clp protease ATP-binding subunit	2.50E-12
O994_RS10610	EF2590		2.4	serine hydrolase: β-lactamase transpeptidase family	1.30E-09
O994_RS10940	EF2664		2.7	phosphoglycerate mutase	1.50E-08
O994_RS11270	EF2734		2.4	Gfo/Idh/MocA family oxidoreductase	5.40E-17
O994_RS00080	EF3282	*clpC*	2.9	Clp protease ATP-binding subunit	4.40E-34
O994_RS13900	EF0046		-1.7	ribonuclease III family protein	3.50E-04
O994_RS13680	EF0097	*pfoR*	-4.9	regulatory protein	2.80E-72
O994_RS13425	EF0166		-2.2	phage integrase family site specific recombinase	9.40E-05
O994_RS13280	EF0200	*fusA*	-2.6	elongation factor G	2.70E-10
O994_RS13275	EF0201	*tuf*	-1.5	elongation factor Tu	1.30E-02
O994_RS12830	EF0277	*ogt*	-2.2	methylated-DNA--[protein]-cysteine *S*-methyltransferase	5.20E-06
O994_RS02150	EF0678		-4.2	GNAT family *N*-acetyltransferase	3.70E-25
O994_RS01630	EF0706	*clpE*	-1.9	Clp protease ATP-binding subunit	7.90E-06
O994_RS02855	EF0827		-2.3	Gfo/Idh/MocA family oxidoreductase	3.40E-06
O994_RS04100	EF1058		-2.2	universal stress protein	7.80E-05
O994_RS04575	EF1158		-4.0	N4-(*β*-*N*-acetylglucosaminyl)-L-asparaginase	6.00E-15
O994_RS01615	EF1874		-3.0	IS256 family transposase	1.30E-15
O994_RS08500	EF1982	*uspA*	-2.2	universal stress protein	4.40E-05
O994_RS09325	EF2208	*phzF*	-2.3	PhzF family phenazine biosynthesis protein	2.50E-05
O994_RS10290	EF2488	*cpsH*	-4.5	lipoprotein	8.30E-39
O994_RS10300	EF2490	*cpsF*	-3.4	casular polysaccharide biosynthesis protein	2.30E-17

O994_RS10305	EF2491		-2.7	glycosyl transferase group 2 family protein	5.40E-11
O994_RS10310	EF2492		-1.7	glycosyl transferase group 2 family protein	5.30E-02
O994_RS10475	EF2562		-6.6	flavodoxin	4.20E-18
O994_RS01520	EF2972		-1.7	amidinotransferase	9.00E-03
O994_RS01190	EF3032	*btpA*	-4.6	photosystem I biogenesis protein	1.30E-23
O994_RS01175	EF3035		-1.9	universal stress protein	7.60E-05
**Poorly characterised**					
O994_RS14005	EF0026		5.5	hypothetical protein	1.30E-94
O994_RS13065	EF0245		2.0	decarboxylase	1.30E-03
O994_RS12415	EF0419		2.3	hypothetical protein	1.40E-12
O994_RS12205	EF0462		1.9	hypothetical protein	1.20E-02
O994_RS12170	EF0468		4.6	LemA family protein	6.60E-51
O994_RS12165	EF0469		4.6	TPM domain-containing protein	8.20E-56
O994_RS02125	EF0673		2.2	acyltransferase family protein	2.60E-03
O994_RS02180	EF0684		1.4	HD domain-containing protein	3.90E-02
O994_RS02260	EF0699		1.8	AI-2E family transporter	3.30E-03
O994_RS02340	EF0708		2.2	hypothetical protein	7.90E-10
O994_RS02525	EF0747		3.0	DUF1003 domain-containing protein	8.20E-25
O994_RS02695	EF0783		4.3	acetyltransferase	2.30E-67
O994_RS02755	EF0795		3.5	TIGR01212 family radical SAM protein	4.10E-30
O994_RS02765	EF0797		5.4	DUF4097 family β strand repeat protein	3.60E-158
O994_RS02770	EF0798		5.5	DUF1700 domain-containing protein	3.20E-154
O994_RS02785	EF0802		8.1	DUF3955 domain-containing protein	5.00E-84
O994_RS02820	EF0819		3.0	DUF960 domain-containing protein	1.80E-17
O994_RS03245	EF0906		2.0	FUSC family protein	1.20E-02
O994_RS03370	EF0931		3.6	hypothetical protein	7.50E-12
O994_RS03375	EF0932		7.4	hypothetical protein	7.00E-23
O994_RS03740	EF1006		1.5	DUF4828 domain-containing protein	2.50E-03
O994_RS04140	EF1067		4.8	hypothetical protein	8.80E-63
O994_RS04305	EF1102		5.8	YxeA family protein	5.70E-11
O994_RS04320	EF1105		2.1	hypothetical protein	1.90E-03

O994_RS04450	EF1132		5.0	CBS-domain containing hypothetical protein	7.60E-87
O994_RS04855	EF1215		1.9	hypothetical protein	1.90E-02
O994_RS04935	EF1231		7.8	metallophosphoesterase	3.10E-78
O994_RS05020	EF1249	*efba*	2.3	fibronectin/fibrinogen-binding protein	6.50E-09
O994_RS05025	EF1250		1.8	hypothetical protein	2.20E-03
O994_RS05040	EF1254		1.9	putative tryptophan/tyrosine transport system permease protein	1.60E-03
O994_RS05060	EF1258		5.5	hypothetical protein	5.30E-39
O994_RS05080	EF1262		1.5	hypothetical protein	6.30E-02
O994_RS05085	EF1263		1.7	ftsX-like permease	7.60E-03
O994_RS05150	EF1276		1.5	hypothetical protein	6.80E-02
O994_RS05395	EF1315		2.1	hypothetical protein	2.70E-04
O994_RS05490	EF1340		3.8	pheromone cAM373 lipoprotein	2.90E-14
O994_RS05620	EF1366		1.5	hypothetical protein	9.00E-02
O994_RS05650	EF1372		1.5	CBS domain-containing protein	2.50E-02
O994_RS05800	EF1402		2.4	cell division protein	1.60E-12
O994_RS05805	EF1403		2.3	hypothetical protein	1.10E-07
O994_RS05850	EF1412		5.3	hypothetical protein	4.40E-60
O994_RS05860	EF1414		2.2	hypothetical protein	5.20E-05
O994_RS05890	EF1420		1.6	DUF4950 domain-containing protein	2.00E-02
O994_RS05895	EF1421		2.1	ImmA/IrrE family metallo-endopeptidase	2.30E-04
O994_RS06260	EF1490		1.7	hypothetical protein	1.90E-02
O994_RS06315	EF1501		2.2	hypothetical protein	1.20E-07
O994_RS06440	EF1523		1.6	hypothetical protein	1.60E-03
O994_RS06480	EF1532		9.0	hypothetical protein	9.90E-78
O994_RS06485	EF1533		9.0	hypothetical protein	9.20E-135
O994_RS06500	EF1536		2.7	α/β hydrolase	7.40E-14
O994_RS06530	EF1542		3.2	hypothetical protein	1.10E-14
O994_RS06540	EF1544		2.0	helix-turn-helix domain-containing protein	1.50E-03
O994_RS06575	EF1552		1.8	hypothetical protein	6.40E-05
O994_RS06580	EF1553		1.9	tetratricopeptide repeat protein	3.30E-06
O994_RS06585	EF1554		1.7	YpiB family protein	4.90E-03

O994_RS06680	EF1573		1.5	DUF479 domain-containing protein	7.30E-02
O994_RS11790	EF1665		5.7	DUF3955 domain-containing protein	7.30E-23
O994_RS07180	EF1680		1.8	YozE family protein	1.10E-04
O994_RS07275	EF1701		1.6	DNA-binding protein	5.00E-03
O994_RS07380	EF1722	*rluD*	1.5	RluA family pseudouridine synthase	2.90E-02
O994_RS07390	EF1724		1.6	CBS domain-containing protein	6.20E-03
O994_RS07530	EF1751		4.5	putative membrane protein	2.70E-83
O994_RS07535	EF1752		4.6	hypothetical protein	8.20E-97
O994_RS07540	EF1753	*liaX*	5.5	hypothetical protein	6.80E-182
O994_RS07620	EF1771		1.8	yigZ family protein	7.30E-04
O994_RS08145	EF1903		3.0	hypothetical protein	7.50E-25
O994_RS08155	EF1906		2.5	hypothetical protein	1.30E-17
O994_RS08170	EF1909		1.8	hypothetical protein	7.30E-05
O994_RS08200	EF1916	*engB*	3.0	GTP-binding protein	3.80E-11
O994_RS08275	EF1933		3.3	hypothetical protein	9.50E-11
O994_RS08325	EF1946		2.8	hypothetical protein	1.30E-11
O994_RS08330	EF1947		2.8	hypothetical protein	8.90E-13
O994_RS08420	EF1966		1.9	YitT family protein	1.90E-03
O994_RS08925	EF2065		3.9	DUF1033 family protein	4.30E-12
O994_RS09035	EF2148		1.6	hypothetical protein	4.00E-02
O994_RS09045	EF2149		1.4	hypothetical protein	6.00E-02
O994_RS09075	EF2155		1.5	phosphoglucosamine mutase	5.60E-03
O994_RS09080	EF2156		2.0	hypothetical protein	5.40E-08
O994_RS09145	EF2169		1.5	putative glycosylation ligase	3.00E-02
O994_RS09340	EF2211		5.3	YxeA family protein	2.00E-63
O994_RS09360	EF2215		6.1	hypothetical protein	1.40E-79
O994_RS09365	EF2216		5.0	FUSC family protein	1.40E-24
O994_RS09620	EF2373		2.3	hypothetical protein	8.50E-10
O994_RS10035	EF2433		1.5	phosphoglycerate mutase	1.90E-02
O994_RS10125	EF2450		1.5	PDZ domain-containing protein	2.30E-02
O994_RS10205	EF2470		3.9	putative metal-dependent phosphohydrolase	8.60E-51

O994_RS10240	EF2477		2.0	SPFH domain-containing protein	2.50E-08
O994_RS10245	EF2478		2.1	hypothetical protein	7.10E-11
O994_RS10250	EF2479		2.2	TPM domain-containing protein	4.20E-13
O994_RS10345	EF2499		3.8	hypothetical protein	3.50E-34
O994_RS14875	EF2499		3.4	hypothetical protein	2.60E-21
O994_RS10360	EF2502		3.8	cell cycle protein FtsW	7.00E-40
O994_RS10680	EF2606		3.5	DUF1146 domain-containing protein	4.90E-15
O994_RS11075	EF2692		3.3	hypothetical protein	3.60E-13
O994_RS11100	EF2697	*xpaC*	4.2	putative 5-bromo-4-chloroindoyl phosphate hydrolase	2.70E-87
O994_RS11300	EF2740		2.8	*O*-methyltransferase	2.00E-14
O994_RS11445	EF2771		6.8	traX family protein	1.50E-69
O994_RS11505	EF2784		2.6	DUF3042 domain containing protein	5.40E-11
O994_RS11560	EF2796		1.8	hypothetical protein	3.80E-04
O994_RS11605	EF2862		4.9	hypothetical protein	1.80E-48
O994_RS11650	EF2872		1.5	RNA-binding protein	8.60E-02
O994_RS11660	EF2874		2.6	haloacid dehalogenase (HAD) superfamily	1.30E-10
O994_RS11775	EF2893		2.0	hypothetical protein	7.60E-04
O994_RS11945	EF2929		4.4	hypothetical protein	5.00E-38
O994_RS11950	EF2930		3.4	hypothetical protein	6.30E-13
O994_RS01475	EF2982		1.7	phosphoglycerate mutase	1.20E-04
O994_RS01260	EF3018		3.9	hypothetical protein	2.00E-60
O994_RS01100	EF3051		1.9	peptidase C39 family protein	2.60E-03
O994_RS01095	EF3052		1.8	hypothetical protein	1.20E-04
O994_RS01070	EF3057		3.9	hypothetical protein	4.00E-27
O994_RS00975	EF3078		2.4	DUF1054 domain-containing protein	5.10E-05
O994_RS00970	EF3079		2.6	GNAT family N-acetyltransferase	5.40E-09
O994_RS00655	EF3149		2.4	helix-turn-helix domain-containing protein	6.10E-17
O994_RS00650	EF3150		2.9	M16 family peptidase	6.30E-30
O994_RS00645	EF3151		3.1	insulinase family protein	6.60E-30
O994_RS00535	EF3176		5.4	YccF domain-containing protein	2.20E-26
O994_RS00530	EF3177		2.7	hypothetical protein	2.50E-08

O994_RS00520	EF3179	3.0	DUF3298 domain-containing protein	5.30E-07
O994_RS00475	EF3188	1.6	hypothetical protein	6.40E-03
O994_RS00465	EF3191	2.3	α/β hydrolase	5.50E-06
O994_RS00460	EF3192	2.3	isochorismatase	2.20E-06
O994_RS00305	EF3234	1.7	DUF5105 domain-containing protein	2.70E-04
O994_RS00265	EF3239	4.6	zinc ribbon domain-containing protein	2.10E-44
O994_RS00120	EF3273	2.2	hypothetical protein	1.00E-04
O994_RS14110	EF0003	-2.8	RNA binding protein	1.60E-18
O994_RS14055	EF0016	-1.4	DegV family protein	6.00E-02
O994_RS14015	EF0024	-1.7	DUF956 family protein	6.70E-03
O994_RS13890	EF0048	-2.1	NYN domain-containing protein	3.70E-06
O994_RS13790	EF0071	-3.6	putative lipoprotein	2.80E-16
O994_RS01610	EF0101	-3.2	α/β fold family hydrolase	3.00E-26
O994_RS13600	EF0115	-5.2	endoribonuclease L-PSP	9.70E-39
O994_RS13430	EF0165	-2.3	hypothetical protein	3.00E-04
O994_RS12870	EF0269	-5.4	PRD domain-containing protein	6.80E-57
O994_RS12850	EF0273	-2.0	phosphoglycerate mutase	2.00E-02
O994_RS12820	EF0279	-2.1	HD domain-containing protein	3.20E-04
O994_RS12580	EF0383	-6.7	DUF2877 domain-containing protein	1.40E-41
O994_RS14960	EF0383	-7.0	hypothetical protein	9.50E-85
O994_RS12545	EF0392	-2.3	WxL domain-containing protein	3.10E-05
O994_RS12475	EF0405	-3.8	HAD superfamily hydrolase	2.80E-27
O994_RS02005	EF0651	-2.4	M48 family metallopeptidase	6.80E-06
O994_RS02865	EF0829	-3.9	PRD domain-containing protein	5.50E-10
O994_RS02870	EF0830	-3.6	glycine-rich SFCGS family protein	1.30E-09
O994_RS02880	EF0832	-3.2	DUF4311 domain-containing protein	8.10E-08
O994_RS03105	EF0877	-3.7	aldo/keto reductase family oxidoreductase	6.60E-30
O994_RS03340	EF0925	-2.0	hypothetical protein	6.00E-04
O994_RS03430	EF0943	-1.6	hypothetical protein	8.70E-02
O994_RS03775	EF1014	-5.7	hypothetical protein	5.30E-21

O994_RS03780	EF1015		-5.5	DUF3188 domain-containing protein	2.90E-17
O994_RS03880	EF1034	*uvrA*	-2.1	aminoglycoside phosphotransferase family protein	6.70E-05
O994_RS03885	EF1035		-3.3	DUF4767 domain-containing protein	3.00E-11
O994_RS04175	EF1074		-2.5	hypothetical protein	8.80E-07
O994_RS04180	EF1075		-3.5	*N*-acetyltransferase	1.00E-13
O994_RS04235	EF1089		-1.6	DUF805 domain-containing protein	1.30E-02
O994_RS04470	EF1137		-1.7	hypothetical protein	3.70E-02
O994_RS05210	EF1288		-1.7	hypothetical protein	2.80E-02
O994_RS05540	EF1350		-3.2	TMEM175 potassium channel family protein	4.10E-11
O994_RS05595	EF1362		-1.9	hypothetical protein	2.60E-04
O994_RS07065	EF1657		-4.5	hypothetical protein	3.20E-35
O994_RS07245	EF1693		-2.6	KH domain-containing protein	2.20E-08
O994_RS07310	EF1708		-1.8	putative glycosyl hydrolase	5.20E-02
O994_RS15000	EF1945		-4.7	alkaline phosphatase family protein	8.80E-15
O994_RS09310	EF2205		-5.0	putative 4.5S RNA: signal recognition particle	4.10E-17
O994_RS09330	EF2209		-1.6	hypothetical protein	6.00E-02
O994_RS09385	EF2220		-3.4	hypothetical protein	7.00E-09
O994_RS09480	EF2239		-3.4	hypothetical protein	3.00E-12
O994_RS10075	EF2441		-4.8	DUF47 family protein	1.20E-32
O994_RS10490	EF2465		-4.8	DUF3343 domain-containing protein	1.70E-13
O994_RS10485	EF2564		-4.8	hydroxylase accessory protein	3.90E-20
O994_RS10495	EF2566		-5.0	hypothetical protein	3.60E-23
O994_RS10515	EF2570		-5.4	aldehyde oxidoreductase	1.40E-11
O994_RS10570	EF2582		-6.8	chlorohydrolase/aminohydrolase	1.30E-26
O994_RS01685	EF2681		-2.3	HAD family hydrolase	1.10E-04
O994_RS14905	EF2687		-1.7	hypothetical protein	1.70E-02
O994_RS14900	EF2687		-1.7	hypothetical protein	2.60E-02
O994_RS11055	EF2688		-1.4	SNF2 helicase associated domain-containing protein	4.50E-02
O994_RS11550	EF2793		-2.2	DUF805 domain-containing protein	3.40E-07
O994_RS11565	EF2797		-2.0	hypothetical protein	4.80E-04
O994_RS11615	EF2864		-2.1	hypothetical protein	4.70E-09

O994_RS11825	EF2904		-1.9	MBL fold metallo-hydrolase	4.40E-06
O994_RS01170	EF3036		-1.9	thioredoxin family protein	3.40E-06
O994_RS00905	EF3093		-1.8	cupin domain-containing protein	6.00E-04
O994_RS00085	EF3281		-1.8	FUSC family protein	1.30E-02
O994_RS14160	EF3326		-6.5	hypothetical protein	2.40E-65
O994_RS14140	EF3330		-1.5	spoIIIJ-associated protein	2.20E-02
**Replication and repair**					
O994_RS13810	EF0066	*ruvA*	2.5	Holliday junction DNA helicase	7.00E-07
O994_RS13805	EF0067	*ruvB*	2.3	Holliday junction DNA helicase	2.50E-08
O994_RS12990	EF0261	*divIC*	2.7	cell division protein	1.20E-24
O994_RS03575	EF0972		4.1	DNA repair exonuclease	2.10E-53
O994_RS05275	EF1301	*rodA*	4.1	rod shape determining protein	1.50E-14
O994_RS05810	EF1404	*mutS2*	2.0	MutS2 family protein	1.20E-04
O994_RS04535	EF1435	*recU*	3.2	recombination protein U	1.80E-29
O994_RS06460	EF1527	*obgE*	2.8	GTPase	3.50E-14
O994_RS06750	EF1587	*mutT*	4.3	DNA mismatch repair protein	4.90E-37
O994_RS06875	EF1615	*parE*	1.7	DNA topoisomerase IV subunit B	1.70E-03
O994_RS07020	EF1648	*xerC*	4.3	integrase/recombinase	1.40E-71
O994_RS10935	EF2663		2.2	recombinase D	6.70E-07
O994_RS11420	EF2766	*recR*	3.2	recombination protein	4.00E-09
O994_RS01105	EF3050	*tag-2*	2.8	DNA-3-methyladenine glycosylase I	6.70E-07
O994_RS14105	EF0004	*recF*	-2.9	recombination protein F	2.80E-24
O994_RS14100	EF0005	*gyrB*	-2.6	DNA gyrase B subunit	5.00E-12
O994_RS14095	EF0006	*gyrA*	-2.7	DNA gyrase A subunit	1.70E-16
O994_RS12825	EF0278	*tag-1*	-2.1	DNA-3-methyladenine glycosylase I	1.30E-05
O994_RS08005	EF1874		-2.7	IS256 family transposase	1.50E-19
**Signal transduction**					
O994_RS13980	EF0031	*mprF*	1.4	bifunctional lysylphosphatidylglycerol flippase/synthetase	9.50E-02
O994_RS05070	EF1260	*yclR*	3.6	DNA-binding response regulator	3.40E-44
O994_RS05075	EF1261	*yclK*	3.4	sensor histidine kinase	1.10E-36

O994_RS13475	EF1703	*phoP*	1.6	alkaline phosphatase synthesis transcriptional regulatory protein	1.10E-02
O994_RS07295	EF1705		3.0	phosphate-binding protein	2.70E-23
O994_RS07570	EF1759		1.4	phosphate ABC transporter substrate-binding protein	9.90E-02
O994_RS08850	EF2049		5.0	ABC transporter permease	1.00E-68
O994_RS08855	EF2050		6.3	ABC transporter ATP-binding protein	1.30E-104
O994_RS11325	EF2746	*dltD*	2.3	dltD protein	5.30E-16
O994_RS11330	EF2747	*dltC*	2.7	D-alanine-poly(phosphoribitol) ligase subunit 2	2.70E-21
O994_RS11335	EF2748	*dltB*	3.3	basic membrane protein	1.10E-41
O994_RS11860	EF2911	*liaR*	3.6	LuxR family DNA-binding response regulator	4.90E-29
O994_RS11865	EF2912	*liaS*	4.0	sensor histidine kinase	1.20E-48
O994_RS11870	EF2913	*liaF*	3.9	cell wall stress response regulator	4.10E-41
O994_RS01515	EF2973	*phoZ*	1.7	alkaline phosphatase	9.60E-02
O994_RS01065	EF3058		2.6	phosphotyrosine protein phosphatase	1.10E-20
O994_RS14335	EF3289	*croR*	2.8	DNA-binding response regulator	9.90E-25
O994_RS14330	EF3290	*croS*	2.7	sensor histidine kinase	1.40E-22
O994_RS04810	EF1206		-5.7	malate dehydrogenase	2.30E-35
O994_RS04815	EF1207	*maeP*	-8.1	CCS family citrate carrier protein	4.20E-18
O994_RS04820	EF1209		-2.7	two-component system, CitB family, sensor kinase	1.30E-12
O994_RS04825	EF1210		-2.8	two-component system, CitB family, response regulator	1.20E-08
O994_RS06955	EF1633		-1.9	two-component system, response regulator	2.80E-02
O994_RS08900	EF2060	*cydB*	-2.3	cytochrome D ubiquinol oxidase subunit II	7.90E-13
O994_RS08905	EF2061	*cydA*	-2.8	cytochrome D ubiquinol oxidase subunit I	1.00E-29
O994_RS09380	EF2219	*yesM*	-2.0	sensor histidine kinase	8.40E-04
O994_RS00455	EF3193	*lrgB*	-5.4	antiholin-like protein	3.00E-71
O994_RS00450	EF3194	*lrgA*	-5.3	murein hydrolase regulator	2.50E-79
O994_RS14215	EF3315	*citG*	-1.5	triphosphoribosyl-dephospho-CoA synthase	3.70E-02
O994_RS14210	EF3316	*maeE*	-2.8	malic enzyme family protein	7.90E-17
O994_RS14200	EF3318	*citX*	-4.9	2-(5''-triphosphoribosyl)-3'-dephosphocoenzyme-A synthase	1.40E-51
O994_RS14195	EF3319	*citF*	-5.2	citrate lyase subunit α	9.90E-78
O994_RS14190	EF3320	*citE*	-5.8	citrate lyase subunit β	1.80E-85

O994_RS14185	EF3321	*citD*	-5.9	citrate lyase subunit γ	3.50E-72
O994_RS14180	EF3322	*citC*	-5.9	[citrate(pro-3SO-lyase] ligase	4.00E-99
**Transcription**					
O994_RS11875	EF2914	*greA*	4.1	transcription elongation factor	2.70E-44
O994_RS00760	EF3126	*rpoZ*	2.3	DNA-directed RNA polymerase subunit ω	8.80E-05
O994_RS13125	EF0233	*rpoA*	-2.3	DNA-directed RNA polymerase subunit α	1.20E-09
O994_RS00290	EF3237	*rpoC*	-1.8	DNA-directed RNA polymerase subunit β	3.30E-05
**Transcriptional regulators**					
O994_RS13650	EF0103		1.5	ArgR family transcriptional regulator	5.80E-02
O994_RS13615	EF0110		2.3	ArsR family transcriptional regulator	1.10E-04
O994_RS12395	EF0422		2.4	IclR family transcriptional regulator	5.60E-05
O994_RS12190	EF0465		2.8	polyisoprenyl-teichoic acid--peptidoglycan teichoic acid transferase	4.60E-21
O994_RS01990	EF0647		1.8	helix-turn-helix transcriptional regulator	6.80E-02
O994_RS02225	EF0692		1.8	RpiR family phosphosugar-binding transcriptional regulator	8.80E-02
O994_RS02975	EF0850		2.3	PemK family transcriptional regulator	7.70E-08
O994_RS03650	EF0988	*mraZ*	2.5	division/cell wall cluster transcriptional repressor	1.60E-20
O994_RS03735	EF1005		2.5	iron-dependent transcriptional repressor	8.10E-10
O994_RS04360	EF1114		2.1	helix-turn-helix transcriptional regulator	7.60E-10
O994_RS04835	EF1212		3.3	transcriptional regulator	5.10E-35
O994_RS05155	EF1277		2.0	Cro/CI family transcriptional regulator	4.30E-06
O994_RS05280	EF1302		4.0	transcriptional regulator	1.60E-25
O994_RS05285	EF1303		5.0	LysR family transcriptional regulator	1.50E-36
O994_RS05500	EF1342		2.8	MarR family transcriptional regulator	3.20E-06
O994_RS05900	EF1422		2.3	Cro/CI family transcriptional regulator	8.20E-05
O994_RS06450	EF1525		2.4	Fur family transcriptional regulator, ferric uptake regulator	1.40E-11
O994_RS06660	EF1569	*psr*	3.7	transcriptional regulator	8.30E-30
O994_RS06800	EF1599		4.2	Cro/CI family transcriptional regulator	6.80E-16
O994_RS07005	EF1645	*codY*	3.1	transcriptional repressor	2.40E-28
O994_RS07320	EF1710		1.7	LysR family transcriptional regulator	1.10E-02
O994_RS07485	EF1741	*ccpA*	2.4	catabolite control protein A	5.50E-12

O994_RS09100	EF2160	*glnR*	2.0	glutamine synthetase repressor	9.50E-06
O994_RS10010	EF2428		1.6	PadR family regulatory protein	1.80E-02
O994_RS10200	EF2469		1.7	Cro/CI family transcriptional regulator	3.40E-02
O994_RS10575	EF2583		1.8	transcriptional regulator	1.10E-03
O994_RS10830	EF2640		2.3	GntR family transcriptional regulator	9.40E-06
O994_RS11140	EF2703		4.5	LytR family transcriptional regulator	2.70E-20
O994_RS11460	EF2774		2.2	helix-turn-helix transcriptional regulator	2.70E-05
O994_RS11720	EF2886		2.4	MarR family transcriptional regulator	7.70E-17
O994_RS01385	EF3002		2.1	transcriptional regulator	3.60E-05
O994_RS01110	EF3049		3.6	RpiR family phosphosugar-binding transcriptional regulator	7.30E-17
O994_RS01060	EF3059		2.8	TetR family transcriptional regulator	1.20E-18
O994_RS00540	EF3175		3.8	Rrf2 family transcriptional regulator	4.90E-12
O994_RS00125	EF3272		1.8	zinc-binding Cro/CI family transcriptional regulator	6.40E-03
O994_RS00075	EF3283	*ctsR*	3.3	transcriptional regulator of stress and heat shock response	4.20E-25
O994_RS13630	EF0107		-4.4	arginine deiminase pathway regulator	7.60E-08
O994_RS12635	EF0372		-1.8	DNA-binding response regulator	8.20E-03
O994_RS12595	EF0382		-1.5	purine catabolism regulatory protein	8.60E-02
O994_RS02070	EF0663		-2.7	YebC/PmpR family DNA-binding transcriptional regulator	2.00E-22
O994_RS02140	EF0676	*argR*	-1.9	transcriptional regulator of arginine metabolism	1.90E-05
O994_RS02450	EF0731		-2.7	LuxR family transcriptional regulator	3.30E-11
O994_RS02860	EF0828		-3.8	transcription antiterminator	4.90E-13
O994_RS03480	EF0954		-1.8	LacI family sugar-binding transcriptional regulator	1.90E-07
O994_RS05570	EF1357		-2.8	AraC family transcriptional regulator	7.60E-08
O994_RS06375	EF1515		-2.1	β-glucoside operon transcriptional antiterminator	1.70E-03
O994_RS06760	EF1591		-4.0	AraC family transcriptional regulator	1.60E-29
O994_RS07060	EF1656		-4.7	LysR family transcriptional regulator	5.20E-30
O994_RS14740	EF1709		-1.8	GntR family transcriptional regulator	3.30E-02
O994_RS07315	EF1709		-2.2	GntR family transcriptional regulator	1.20E-07
O994_RS01690	EF1839	*lacR*	-2.5	lactose phosphotransferase system repressor	1.30E-11
O994_RS08375	EF1955		-2.3	σ-54 dependent transcriptional regulator	2.10E-07

O994_RS09320	EF2207		-2.5	Fis family transcriptional regulator	3.20E-08
O994_RS12030	EF2966		-8.9	mltR-like mannitol-operon transcriptional regulator	9.10E-125
O994_RS00680	EF3144		-1.8	RpiR family phosphosugar-binding transcriptional regulator	4.20E-02
O994_RS00355	EF3216		-2.9	σ-54-interacting transcriptional regulator	4.90E-18
O994_RS00100	EF3278		-1.5	PucR family transcriptional regulator	2.20E-02
O994_RS14250	EF3308	*srlR*	-5.4	transcriptional regulator	8.70E-16
O994_RS14245	EF3309	*srlM*	-5.7	lichenan operon transcriptional antiterminator	4.80E-37
O994_RS14150	EF3328	*citO*	-3.7	GntR family transcriptional regulator	1.80E-29
**Translation**					
O994_RS12980	EF0263		3.2	tRNA(Ile)-lysidine synthase	1.10E-21
O994_RS12960	EF0267		3.3	zinc-binding NifR3 family TIM-barrel protein	1.20E-19
O994_RS02110	EF0670		3.2	rRNA pseudouridine synthase	8.60E-13
O994_RS02155	EF0679		2.1	RluA family pseudouridine synthase	1.10E-03
O994_RS02270	EF0701	*prfc*	2.3	peptide chain release factor 3	3.60E-06
O994_RS02530	EF0748	*trhO*	1.8	tRNA uridine(34) hydroxylase	1.10E-03
O994_RS03465	EF0950		4.1	tRNA threonylcarbamoyladenosine biosynthesis protein	2.40E-11
O994_RS03655	EF0989	*rsmH*	2.5	16S rRNA (cytosine(1402)-N(4))-methyltransferase	2.40E-22
O994_RS05095	EF1265		2.8	23S rRNA (cytosine1962-C5)-methyltransferase	1.80E-13
O994_RS07025	EF1649	*gid*	2.3	tRNA (uracil-5-)-methyltransferase	1.60E-07
O994_RS07415	EF1730		1.8	Cys-tRNA(Pro)/Cys-tRNA(Cys) deacylase	1.70E-03
O994_RS09110	EF2162	*miaA*	1.6	tRNA δ(2)-isopentenylpyrophosphate transferase	7.70E-02
O994_RS09610	EF2371	*asnS*	1.5	asparaginyl-tRNA synthetase	4.80E-02
O994_RS09645	EF2379	*proS*	1.5	prolyl-tRNA synthetase	3.10E-02
O994_RS10135	EF2452	*rsmD*	1.6	16S rRNA (guanine(966)-N(2))-methyltransferase	6.40E-03
O994_RS10225	EF2474		1.6	[ribosomal protein S18]-alanine *N*-acetyltransferase	5.90E-02
O994_RS11010	EF2679	*trpS*	2.6	tryptophanyl-tRNA synthetase	1.10E-10
O994_RS11155	EF2706		1.8	23S rRNA (uracil(1939)-C(5))-methyltransferase	1.90E-02
O994_RS11255	EF2731	*rpmG-2*	3.5	50S ribosomal protein L33	1.20E-12
O994_RS11575	EF2856	*rpmG-3*	2.1	50S ribosomal protein L33	1.20E-07
O994_RS11655	EF2873	*yqeH*	2.3	30S ribosome assembly GTPase	3.10E-08
O994_RS00390	EF3207		2.2	dihydrouridine synthase	2.30E-04

O994_RS14285	EF3300	*gidB*	2.3	16S rRNA methyltransferase	2.60E-04
O994_RS13905	EF0045	*cysS*	-1.6	cysteinyl-tRNA synthetase	3.30E-03
O994_RS13895	EF0047	*rlmB*	-1.9	23S rRNA (guanosine(2251)-2'-*O*)-methyltransferase	4.30E-07
O994_RS13665	EF0100	*serS-1*	-5.1	seryl-tRNA synthetase	1.50E-73
O994_RS13290	EF0198	*rpsL*	-1.8	30S ribosomal protein S12	3.60E-03
O994_RS13285	EF0199	*rpsG*	-2.3	30S ribosomal protein S7	1.10E-06
O994_RS13260	EF0205	*rpsJ*	-2.0	30S ribosomal protein S10	4.80E-05
O994_RS13255	EF0206	*rplC*	-2.3	50S ribosomal protein L3	4.90E-08
O994_RS13250	EF0207	*rplD*	-2.5	50S ribosomal protein L4	4.60E-11
O994_RS13245	EF0208	*rplW*	-2.4	50S ribosomal protein L23	3.00E-08
O994_RS13240	EF0209	*rplB*	-2.7	50S ribosomal protein L2	2.50E-12
O994_RS13235	EF0210	*rpsS*	-2.7	30S ribosomal protein S19	8.00E-11
O994_RS13230	EF0211	*rplV*	-2.7	50S ribosomal protein L22	8.10E-12
O994_RS13225	EF0212	*rpsC*	-2.8	30S ribosomal protein S3	1.40E-12
O994_RS13220	EF0213	*rplP*	-2.9	50S ribosomal protein L16	1.20E-16
O994_RS13215	EF0214	*rpmC*	-3.0	50S ribosomal protein L29	1.70E-15
O994_RS13210	EF0215	*rpsQ*	-2.9	30S ribosomal protein S3	2.20E-16
O994_RS13205	EF0216	*rplN*	-3.1	50S ribosomal protein L14	1.20E-18
O994_RS13200	EF0217	*rplX*	-3.2	50S ribosomal protein L24	2.20E-20
O994_RS13195	EF0218	*rplE*	-3.1	50S ribosomal protein L5	6.50E-23
O994_RS13185	EF0220	*rpsH*	-3.3	30S ribosomal protein S8	2.60E-21
O994_RS13180	EF0221	*rplF*	-3.3	50S ribosomal protein L6	9.50E-28
O994_RS13175	EF0223	*rplR*	-3.3	50S ribosomal protein L18	4.00E-33
O994_RS13170	EF0224	*rpsE*	-3.4	30S ribosomal protein S5	4.40E-25
O994_RS13165	EF0225	*rpmD*	-3.3	50S ribosomal protein L30	7.50E-24
O994_RS13160	EF0226	*rplO*	-3.5	50S ribosomal protein L15	8.10E-30
O994_RS13145	EF0229	*infA*	-1.9	translation initiation factor IF-1	1.10E-03
O994_RS13140	EF0230	*rpmJ*	-2.1	50S ribosomal protein L36	3.60E-06
O994_RS13135	EF0231	*rpsM*	-2.2	30S ribosomal protein S13	1.20E-07
O994_RS13130	EF0232	*rpsK*	-2.2	30S ribosomal protein S11	2.30E-07

O994_RS13120	EF0234	*rplQ*	-2.2	50S ribosomal protein L17	4.70E-08
O994_RS02420	EF0725	*gatA*	-1.4	Asp-tRNA/Glu-tRNA amidotransferase	6.30E-02
O994_RS02425	EF0726	*gatB*	-1.7	aspartyl/glutamyl-tRNA amidotransferase	1.90E-05
O994_RS02780	EF0801	*leuS*	-1.5	leucyl-tRNA synthetase	2.60E-03
O994_RS02915	EF0838		-2.1	pyridoxal phosphate-dependent enzyme	3.50E-03
O994_RS05135	EF1274	*infB*	-1.5	translation initiation factor IF-2	2.70E-02
O994_RS05140	EF1275	*rbfA*	-1.5	ribosome-binding factor A	5.00E-02
O994_RS07250	EF1694	*rpsP*	-2.4	30S ribosomal protein S16	2.60E-09
O994_RS07595	EF1764	*yfiA*	-1.6	ribosomal subunit interface protein	9.10E-03
O994_RS09860	EF2395	*frr*	-2.7	ribosome recycling factor	3.70E-16
O994_RS09905	EF2406	*glyS*	-1.6	glycyl-tRNA synthetase subunit β	5.70E-03
O994_RS11185	EF2715	*rplL*	-2.9	50S ribosomal protein L7/L12	8.20E-27
O994_RS11190	EF2716	*rplJ*	-2.7	50S ribosomal protein L10	1.40E-17
O994_RS11195	EF2718	*rplA*	-1.4	50S ribosomal protein L1	4.30E-02
O994_RS11955	EF2931	*valS*	-1.7	valyl-tRNA synthetase	1.20E-04
O994_RS01220	EF3025	*rlmH*	-2.0	23S rRNA (pseudouridine1915-N3)-methyltransferase	1.00E-05
O994_RS00415	EF3202	*rpsN*	-3.2	30S ribosomal protein S14	8.30E-23
O994_RS14325	EF3292	*serS-2*	-2.6	seryl-tRNA synthetase	4.20E-18
O994_RS14235	EF3311	*gidA*	-1.9	tRNA uridine 5-carboxymethylaminomethyl modification protein	4.10E-04
**Unclassified: genetic information processing**					
O994_RS12985	EF0262		2.7	hypothetical protein	1.80E-15
O994_RS12145	EF0473	*nrdH*	2.2	ribonucleoside-diphosphate reductase 2	1.70E-10
O994_RS05375	EF1312		1.4	S1 RNA-binding domain-containing protein	3.50E-02
O994_RS08550	EF1991	*cspC*	1.5	cold shock protein	4.40E-02
O994_RS11000	EF2677	*mecA*	2.1	adaptor protein	2.00E-08
O994_RS10480	EF2563		-5.6	hypothetical protein	7.90E-13
**Unclassified: metabolism**					
O994_RS02575	EF0758		1.8	cadmium-translocating P-type ATPase	4.30E-02
O994_RS04905	EF1225	*apbE*	2.0	thiamin biosynthesis	5.90E-05
O994_RS05105	EF1268		2.8	cation transporter E1-E2 family ATPase	4.40E-28

O994_RS05290	EF1304	*mgtA-2*	4.4	magnesium-translocating P-type ATPase	1.80E-37
O994_RS05370	EF1311		2.1	hypothetical protein	2.50E-08
O994_RS05790	EF1400		1.8	cadmium-translocating P-type ATPase	5.70E-03
O994_RS06425	EF1519		3.4	cation transporter E1-E2 family ATPase	1.60E-27
O994_RS07185	EF1681	*msrA*	1.8	peptide methionine sulfoxide reductase	2.30E-05
O994_RS09085	EF2157		2.1	hypothetical protein	3.30E-09
O994_RS10355	EF2501		3.1	arsenate reductase	8.00E-20
O994_RS10655	EF2601		1.6	acyl carrier protein	3.80E-02
O994_RS01390	EF3001		1.9	protease synthase and sporulation negative regulatory protein	1.40E-03
O994_RS00595	EF3164	*msrB*	3.4	methionine sulfoxide reductase B	1.20E-18
O994_RS14275	EF3303		2.1	myosin-cross-reactive antigen	9.70E-13
O994_RS14075	EF0011		-2.0	DHH family protein	6.30E-09
O994_RS12375	EF0426		-3.0	gluconate 5-dehydrogenase	1.90E-08
O994_RS03095	EF0875		-1.6	copper-translocating P-type ATPase	6.00E-03
O994_RS05530	EF1348		-2.3	glucan 1	5.50E-11
O994_RS09740	EF1511		-2.1	mandelate racemase	4.00E-03
O994_RS07755	EF1800		-1.5	hypothetical protein	8.50E-02
O994_RS08225	EF1921	*rihC*	-5.0	ribonucleoside hydrolase	1.20E-30
O994_RS10470	EF2561		-5.2	ferredoxin-NADP(+) reductase subunit α	2.20E-31
O994_RS11295	EF2739	*ahpC*	-1.5	alkyl hydroperoxide reductase subunit C	5.40E-04
O994_RS00750	EF3129		-1.6	D-alanyl-D-alanine carboxypeptidase	5.80E-03
O994_RS00695	EF3140		-7.1	iron-containing alcohol dehydrogenase	3.90E-25
**Xenobiotics biodegradation and metabolism**					
O994_RS11590	EF2859		2.2	4-oxalocrotonate tautomerase	1.30E-03
O994_RS02475	EF0737		5.5	amidase	4.80E-105
O994_RS11590	EF2859		2.2	4-oxalocrotonate tautomerase	1.30E-03
**JH2-2 Specific Genes**					
O994_RS01040			2.2	ABC transporter permease	6.60E-05
O994_RS01045			5.1	α/β hydrolase	8.60E-39
O994_RS01050			2.0	GNAT family *N*-acetyltransferase	1.20E-05

O994_RS01355		1.8	ABC transporter permease	1.00E-02
O994_RS01705		3.2	hypothetical protein	1.00E-09
O994_RS01800	*uvrB*	3.0	DNA helicase	6.50E-08
O994_RS01805		1.6	tRNA-al	4.40E-02
O994_RS01810		1.6	hypothetical protein	1.70E-02
O994_RS02075		3.0	hypothetical protein	2.30E-06
O994_RS03850		3.9	hypothetical protein	9.40E-11
O994_RS06250		2.2	IS30-like element IS6770 family transposase	7.50E-04
O994_RS06415		3.3	hypothetical protein	2.80E-14
O994_RS07475		2.7	ABC transporter permease	6.70E-08
O994_RS08365		2.3	hypothetical protein	7.40E-07
O994_RS08370	*mreC*	4.4	rod shape-determining protein	7.70E-37
O994_RS08380	*mreD*	4.4	rod shape-determining protein	8.20E-15
O994_RS09570		2.8	hypothetical protein	1.20E-09
O994_RS12175		4.1	hypothetical protein	2.90E-20
O994_RS13455		1.6	type 1 glutamine amidotransferase	7.60E-02
O994_RS14175		6.1	hypothetical protein	1.20E-59
O994_RS14525		3.4	hypothetical protein	4.30E-12
O994_RS14560		2.0	hypothetical protein	2.50E-05
O994_RS14915		3.0	hypothetical protein	1.10E-08
O994_RS14975		5.4	hypothetical protein	2.40E-95
O994_RS14980		8.3	hypothetical protein	1.60E-16
O994_RS15025		3.8	recombinase family protein	5.10E-19
O994_RS00340	*lacC*	-6.2	tagatose-6-phosphate kinase	2.10E-17
O994_RS00345		-7.2	PTS sugar transporter subunit IIB	1.90E-18
O994_RS00660		-7.0	PTS fructose transporter subunit IIA	2.90E-14
O994_RS01230		-1.7	PD-(D/E)XK motif protein	5.40E-04
O994_RS01545		-1.7	AIPR family protein	8.60E-05
O994_RS01815		-2.2	tRNA-Ile	1.90E-04
O994_RS03080		-3.6	type I restriction endonuclease subunit R	2.10E-44

O994_RS04000	-1.6	DUF262 domain-containing protein	5.50E-02
O994_RS04075	-1.9	multicopper oxidase domain-containing protein	4.50E-05
O994_RS06245	-6.5	hypothetical protein	6.20E-74
O994_RS08540	-2.0	hypothetical protein	7.30E-06
O994_RS13460	-5.7	hypothetical protein	1.60E-50
O994_RS13620	-6.3	hypothetical protein	4.00E-54
O994_RS14690	-1.8	DDE-type integrase/transposase/recombinase	3.50E-03
O994_RS14750	-1.4	hypothetical protein	2.20E-02
O994_RS14770	-2.3	hypothetical protein	3.50E-07
O994_RS14805	-3.0	hypothetical protein	1.30E-15
O994_RS14955	-7.9	hypothetical protein	3.20E-10
O994_RS14965	-1.6	hypothetical protein	8.60E-02

Table S4. Genes differentially expressed in *E. faecalis* Δ*croRS* in the presence versus absence of TXB.

JH2-2 Locus Tag	V583 Gene	Gene Name	F/C (log_2)	Gene Function	P_{adj}
**Amino acid Metabolism**					
O994_RS02475	EF0737		2.4	amidase	8.30E-11
O994_RS04600	EF1163		1.5	L-asparaginase	3.90E-02
O994_RS13680	EF0097	*pfoR*	-3.1	regulatory protein	9.50E-10
O994_RS13675	EF0098	*sdhB-1*	-3.4	L-serine dehydratase, iron-sulfur-dependent subunit β	6.10E-10
O994_RS13670	EF0099	*sdhA-1*	-3.5	iron-sulfur-dependent L-serine dehydratase subunit α	2.00E-14
O994_RS13600	EF0115		-2.7	endoribonuclease L-PSP	2.60E-05
O994_RS03180	EF0891		-2.1	aspartate aminotransferase	2.10E-04
O994_RS05390	EF1314	*alaT*	-1.6	aminotransferase	6.90E-03
O994_RS06620	EF1561	*aroE*	-2.3	shikimate 5-dehydrogenase	7.10E-09
O994_RS06625	EF1562		-2.1	3-deoxy-7-phosphoheptulonate synthase	1.70E-07
O994_RS06630	EF1563	*aroB*	-1.8	3-dehydroquinate synthase	8.80E-03
O994_RS06635	EF1564	*aroC*	-2.1	chorismate synthase	5.90E-04
O994_RS06640	EF1565		-2.2	prephenate dehydrogenase	2.10E-05
O994_RS06645	EF1566	*aroA*	-2.1	3-phosphoshikimate 1-carboxyvinyltransferase	7.60E-06
O994_RS06650	EF1567	*aroK*	-2.4	shikimate kinase	4.50E-10
O994_RS06655	EF1568		-2.6	prephenate dehydratase	2.90E-10
O994_RS06885	EF1617		-1.7	hypothetical protein	9.70E-02
O994_RS06930	EF1626	*eutL*	-2.0	ethanolamine utilization protein	1.20E-02
O994_RS06960	EF1634	*pduU*	-2.0	propanediol utilization protein	2.70E-03
O994_RS06975	EF1638	*pduV*	-2.6	propanediol utilization protein	4.70E-06
**Autolysis**					
O994_RS12300	EF0443		4.1	LysM domain-containing protein	1.30E-17
O994_RS02775	EF0799		-2.2	autolysin	6.30E-07
**Carbohydrate Metabolism**					
O994_RS02385	EF0718	*pfkB*	2.1	1-phosphofructokinase	2.80E-06

O994_RS03410	EF0939	*mgsA*	1.6	methylglyoxal synthase	7.20E-03
O994_RS05845	EF1411		3.1	6-phospho-β-glucosidase	3.10E-17
O994_RS06455	EF1526	*gap-1*	1.8	glyceraldehyde 3-phosphate dehydrogenase	1.00E-04
O994_RS06790	EF1597	*katA*	4.3	catalase/peroxidase	2.00E-46
O994_RS14270	EF3304	*mipB*	2.5	fructose-6-phosphate aldolase	3.60E-07
O994_RS13605	EF0114		-1.8	glycosyl hydrolase	4.10E-05
O994_RS13385	EF0174	*deoC*	-1.6	2-deoxyribose-5-phosphate aldolase	7.90E-02
O994_RS13295	EF0197	*rpiA*	-1.6	ribose-5-phosphate isomerase A	1.60E-02
O994_RS13025	EF0253		-2.5	aldehyde dehydrogenase	9.70E-05
O994_RS12860	EF0271	*arb*	-2.1	6-phospho-β-glucosidase	2.30E-02
O994_RS12855	EF0272		-2.3	glycosyl hydrolase	5.10E-05
O994_RS12440	EF0413	*mtlD*	-3.4	mannitol-1-phosphate 5-dehydrogenase	8.30E-15
O994_RS01960	EF0641	*ldh2*	-2.3	L-lactate dehydrogenase	2.60E-05
O994_RS02145	EF0677		-1.5	phosphoglucomutase/ phosphomannomutase	8.50E-02
O994_RS03215	EF0900	*adhE*	-2.5	bifunctional acetaldehyde-CoA/alcohol dehydrogenase	1.20E-06
O994_RS03460	EF0949	*eutD*	-1.9	phosphotransacetylase	8.90E-04
O994_RS04150	EF1069	*galK*	-2.2	galactokinase	3.40E-03
O994_RS04155	EF1070	*galE-1*	-2.1	UDP-glucose 4-epimerase	1.40E-02
O994_RS04330	EF1108	*lutA*	-2.1	oxidoreductase	4.10E-03
O994_RS04335	EF1109		-2.0	iron-sulfur cluster binding protein	4.80E-03
O994_RS04340	EF1110		-1.5	YkgG family protein	8.80E-02
O994_RS01725	EF1243		-2.2	glycosyl hydrolase	2.20E-03
O994_RS05525	EF1347		-1.6	glycosyl hydrolase	9.00E-02
O994_RS05535	EF1349		-2.4	glycosyl hydrolase	6.00E-06
O994_RS05575	EF1358	*gldA*	-2.2	glycerol dehydrogenase	5.90E-07
O994_RS06865	EF1613	*pflB*	-3.4	formate acetyltransferase	7.40E-03
O994_RS06875	EF1618	*eutH*	-2.2	ethanolamine utilization protein	4.20E-06
O994_RS06905	EF1621		-2.1	phosphate propanoyltransferase	1.20E-02
O994_RS06945	EF1630	*eutA*	-2.1	ethanolamine utilisation protein	1.40E-02
O994_RS06965	EF1635	*pduQ*	-2.2	propanol dehydrogenase	6.50E-03

O994_RS01700	EF1834	*lacB*	-3.6	galactose-6-phosphate isomerase subunit	9.80E-19
O994_RS01695	EF1835	*lacA*	-3.6	galactose-6-phosphate isomerase subunit	9.50E-18
O994_RS09055	EF2151	*glmS*	-1.5	glucosamine--fructose-6-phosphate aminotransferase	8.80E-03
O994_RS10285	EF2487	*glf*	-2.3	UDP-galactopyranose mutase	2.80E-07
O994_RS10350	EF2500		-1.7	GcvH family protein	2.60E-02
O994_RS10460	EF2559		-2.1	pyruvate flavodoxin/ferredoxin oxidoreductase family protein	7.00E-06
O994_RS12005	EF2961	*rbsK*	-2.4	ribokinase	5.30E-07
O994_RS00720	EF3135	*uxuA*	-3.2	mannonate dehydratase	6.00E-09
O994_RS00715	EF3136		-3.0	PTS system transporter subunit IIA	8.80E-06
O994_RS00700	EF3139		-3.0	PTS system transporter subunit IIC	1.90E-08
O994_RS00690	EF3141		-2.9	D-isomer specific 2-hydroxyacid dehydrogenase family protein	3.90E-08
O994_RS00685	EF3142	*gnd*	-2.4	6-phosphogluconate dehydrogenase	2.00E-04
O994_RS00615	EF3158	*pgmB*	-3.3	HAD superfamily hydrolase	6.30E-18
O994_RS00600	EF3163	*prsA-2*	-2.0	ribose-phosphate pyrophosphokinase	5.50E-05
O994_RS14205	EF3317	*citM*	-2.4	oxaloacetate decarboxylase	4.40E-06
O994_RS14170	EF3324		-1.9	sodium ion-translocating decarboxylase subunit β	1.30E-02
O994_RS14165	EF3325		-4.3	acetyl-CoA carboxylase biotin carboxyl carrier protein subunit	3.10E-13
**Cellular community**					
O994_RS01710	EF1807	*lacD-2*	-3.4	tagatose 1,6-diphosphate aldolase	1.80E-13
O994_RS00880	EF3106	*opp2A*	-3.1	oligopeptide ABC transporter substrate-binding protein	9.90E-19
O994_RS00875	EF3107	*opp2C*	-3.6	oligopeptide ABC transporter permease	3.70E-23
O994_RS00865	EF3109	*opp2F*	-4.2	oligopeptide ABC transporter ATP-binding protein	2.50E-31
O994_RS00860	EF3110	*opp2D*	-3.9	oligopeptide ABC transporter ATP-binding protein	2.50E-31
**Drug resistance: antimicrobial**					
O994_RS11105	EF2698	*telA*	3.4	tellurite resistance protein	1.80E-19
**Energy Metabolism**					
O994_RS06270	EF1492		2.1	V-type ATPase subunit F	6.70E-07
O994_RS06275	EF1493		1.9	V-type ATP synthase subunit I	4.30E-06
O994_RS06280	EF1494		1.6	V-type ATP synthase subunit K	2.10E-03
O994_RS06285	EF1495		1.6	V-type ATPase subunit E	3.00E-03
O994_RS06290	EF1496		1.6	V-type ATPase subunit C	8.80E-03

O994_RS06310	EF1500		1.6	V-type ATP synthase subunit D	7.40E-03
O994_RS05865	EF1415	*gdhA*	-1.8	glutamate dehydrogenase	1.10E-03
O994_RS10465	EF2560	*gltA*	-2.9	oxidoreductase	6.90E-07
O994_RS00200	EF3257		-1.8	pyridine nucleotide-disulfide family oxidoreductase	2.20E-02
**Folding, sorting and degradation**					
O994_RS06545	EF1545	*recQ-1*	1.8	ATP-dependent DNA helicase	1.30E-02
O994_RS13155	EF0227	*secY*	-1.8	preprotein translocase subunit	5.20E-08
O994_RS03205	EF0897	*yajC*	-1.6	preprotein translocase subunit	5.50E-02
**Glycan biosynthesis and metabolism**					
O994_RS02085	EF0666		1.6	glyoxalase	2.60E-03
O994_RS02160	EF0680		3.1	penicillin-binding protein 2A	2.40E-17
O994_RS11345	EF2750	*dltX*	3.1	teichoic acid D-ala incorporation-associated protein	3.50E-30
O994_RS12535	EF0394	*salB*	-3.0	peptidoglycan DL-endopeptidase	4.80E-14
O994_RS04630	EF1169	*murAB*	-1.8	UDP-*N*-acetylglucosamine 1-carboxyvinyltransferase	1.70E-03
O994_RS04645	EF1172		-2.1	teichoic acid biosynthesis protein	2.50E-05
O994_RS09170	EF2174		-2.5	putative L-alanyl-D-glutamate peptidase	7.50E-07
O994_RS10070	EF2440		-2.3	celC-like protein	1.00E-05
O994_RS14400	EF2489	*cspG*	-2.8	MurB family protein	1.80E-19
O994_RS01055	EF3060		-2.4	peptidoglycan DL-endopeptidase	1.30E-07
**Lipid Metabolism**					
O994_RS06840	EF1608		1.6	cardiolipin synthase	1.40E-02
O994_RS08150	EF1904		2.6	glycerophosphoryl diester phosphodiesterase family protein	1.10E-10
O994_RS11070	EF2691	*plsC*	2.2	1-acyl-sn-glycerol-3-phosphate acyltransferase	4.20E-08
O994_RS00665	EF3148	*pgsA*	1.5	CDP-diacylglycerol--glycerol-3-phosphate 3-phosphatidyltransferase	9.00E-02
O994_RS05580	EF1359	*dhaM*	-2.5	dihydroxyacetone kinase	1.60E-09
O994_RS05585	EF1360	*dhaK*	-2.5	dihydroxyacetone kinase	1.10E-09
O994_RS05590	EF1361	*dhaL*	-2.6	dihydroxyacetone kinase	1.80E-10

O994_RS06935	EF1627	*eutC*	-1.9	ethanolamine ammonia-lyase small subunit	1.70E-02
O994_RS06940	EF1629	*eutB*	-1.8	ethanolamine ammonia-lyase large subunit	4.30E-02
O994_RS08255	EF1928	*glpO*	-1.9	α-glycerophosphate oxidase	7.40E-03
O994_RS10860	EF2646		-2.5	glycerate kinase	3.10E-04
**Membrane transport**					
O994_RS12425	EF0417		2.6	ABC transporter permease	1.40E-05
O994_RS12420	EF0418		2.4	ABC transporter ATP-binding protein	8.60E-05
O994_RS02130	EF0674		2.5	glycine betaine/carnitine/choline ABC transporter	1.00E-10
O994_RS02135	EF0675		2.5	glycine betaine/carnitine/choline ABC transporter	2.80E-09
O994_RS02380	EF0717		2.2	PTS system fructose-specific transporter subunit IIABC	1.00E-08
O994_RS02485	EF0739		1.9	nicotinamide riboside transporter	4.50E-05
O994_RS03025	EF0859		1.7	cation efflux family protein	7.40E-02
O994_RS03085	EF0872		2.9	KUP system potassium uptake protein	1.30E-22
O994_RS03920	EF1042		1.9	multidrug efflux MFS transporter	5.40E-05
O994_RS04740	EF1192		2.3	MIP family channel protein	1.20E-08
O994_RS13465	EF1408		2.5	ABC-2 type transport system ATP-binding protein	8.10E-08
O994_RS06525	EF1541		2.3	riboflavin transporter	6.50E-08
O994_RS07140	EF1672		3.8	putative ABC transport system permease protein	8.50E-24
O994_RS07145	EF1673		3.9	putative ABC transport system ATP-binding protein	1.70E-32
O994_RS07635	EF1774		2.2	QueT transporter family protein	9.80E-06
O994_RS13495	EF1878		3.6	ATP/GTP-binding protein	2.00E-07
O994_RS08940	EF2068		2.0	multidrug efflux MFS transporter	2.50E-07
O994_RS09415	EF2226		4.1	ABC transporter ATP-binding protein/permease	1.60E-32
O994_RS09420	EF2227		4.2	ABC transporter ATP-binding protein/permease	1.50E-27
O994_RS10395	EF2509		1.8	AzlC family ABC transporter permease	2.60E-03
O994_RS10835	EF2641		2.0	glycine betaine/L-proline ABC transporter ATP-binding protein	7.20E-03
O994_RS10840	EF2642		1.9	glycine betaine/L-proline ABC transporter	7.20E-03
O994_RS10875	EF2649		1.9	spermidine/putrescine ABC transporter	2.80E-05
O994_RS10880	EF2650		1.9	spermidine/putrescine ABC transporter permease	9.80E-06
O994_RS10885	EF2651		1.9	spermidine/putrescine ABC transporter permease	4.70E-05
O994_RS10890	EF2652		1.8	spermidine/putrescine ABC transporter ATP-binding protein	1.10E-05

O994_RS11015	EF2680		1.6	ABC transporter ATP-binding protein/permease	3.70E-03
O994_RS11430	EF2768		2.7	hypothetical protein	1.70E-06
O994_RS11435	EF2769		2.7	energy-coupling factor transport system ATP-binding protein	8.80E-12
O994_RS11440	EF2770		2.7	energy-coupling factor transport system permease protein	3.00E-11
O994_RS11975	EF2935		1.5	xanthine/uracil permeases family protein	5.10E-02
O994_RS01460	EF2985		4.1	putative ABC transport system permease protein	5.40E-37
O994_RS01455	EF2986		3.6	putative ABC transport system ATP-binding protein	1.20E-18
O994_RS01450	EF2987		3.2	HlyD family efflux transporter periplasmic adaptor subunit	1.00E-16
O994_RS01090	EF3053	*thiT*	1.5	thiamine transporter	7.80E-02
O994_RS00640	EF3152	*mscL*	1.7	large conductance mechanosensitive channel protein	2.90E-03
O994_RS13840	EF0063		-2.0	pheromone binding protein	3.70E-04
O994_RS13740	EF0082		-2.8	major facilitator family transporter	1.60E-06
O994_RS13695	EF0094		-3.3	formate/nitrite transporter family protein	3.10E-09
O994_RS13375	EF0176		-1.9	hypothetical protein	2.60E-03
O994_RS13370	EF0177		-1.6	hypothetical protein	1.60E-02
O994_RS13365	EF0178		-2.3	ABC transporter ATP-binding protein	2.90E-10
O994_RS13360	EF0179		-2.2	ABC transporter permease	4.70E-08
O994_RS13355	EF0180		-2.1	ABC transporter permease	1.20E-06
O994_RS12865	EF0270		-2.4	PTS system β-glucoside-specific transporter subunit IIABC	7.40E-07
O994_RS12490	EF0402	*nhaC-1*	-1.8	Na^+/H^+ antiporter	2.00E-03
O994_RS12450	EF0411		-4.7	PTS system mannitol-specific transporter subunit IIBC	2.30E-14
O994_RS12445	EF0412	*mltF*	-4.9	PTS system mannitol-specific transporter subunit IIA	1.60E-13
O994_RS12085	EF0516		-1.9	PTS sugar transporter subunit IIC	9.70E-03
O994_RS03250	EF0907		-1.6	peptide ABC transporter peptide-binding protein	5.90E-03
O994_RS03405	EF0938	*ugpC*	-2.2	sugar ABC transporter ATP-binding protein	2.60E-05
O994_RS01715	EF1018		-3.0	PTS system transporter subunit IIA	7.40E-07
O994_RS01720	EF1160	*celB*	-2.4	PTS system cellobiose-specific transporter subunit IIC	4.60E-04
O994_RS04950	EF1234		-2.7	ABC transporter substrate-binding protein	3.60E-06
O994_RS06985	EF1639	*btuC*	-1.9	cobalamin transport system ATP-binding protein	7.70E-03
O994_RS06990	EF1641		-2.3	cobalamin transport system substrate-binding protein	1.50E-04

O994_RS07370	EF1720		-2.1	uracil permease	6.00E-03
O994_RS09350	EF2213		-3.9	PTS system transporter subunit IIBC	5.60E-27
O994_RS09390	EF2221		-2.8	ABC transporter substrate-binding protein	1.20E-12
O994_RS09395	EF2222		-2.3	ABC transporter permease	2.30E-06
O994_RS09400	EF2223		-3.9	putative aldouronate transport system permease protein	2.50E-20
O994_RS10080	EF2442	*PiT*	-2.9	inorganic phosphate transporter, PiT family	1.40E-11
O994_RS10275	EF2485	*cpsK*	-1.8	ABC transporter permease	1.20E-03
O994_RS10280	EF2486	*cpsJ*	-2.1	ABC transporter ATP-binding protein	1.70E-06
O994_RS10865	EF2647	*grtP*	-2.3	gluconate:H^+ symporter, GntP family	1.50E-03
O994_RS11995	EF2959	*rbsU*	-1.6	ribose uptake protein	3.00E-02
O994_RS12000	EF2960	*rbsD*	-2.5	D-ribose pyranase	3.80E-07
O994_RS12020	EF2964	*ulaA*	-4.1	PTS system ascorbate-specific transporter subunit IIC	1.00E-16
O994_RS12025	EF2965		-4.5	hypothetical protein	9.60E-17
O994_RS00870	EF3108	*opp2B*	-3.8	oligopeptide ABC transporter permease	1.10E-30
O994_RS00710	EF3137		-2.7	PTS system transporter subunit IIB	1.20E-05
O994_RS00705	EF3138		-2.8	PTS system transporter subunit IID	3.20E-07
O994_RS00620	EF3157		-3.4	glycosyl hydrolase	1.00E-22
O994_RS00385	EF3208		-1.8	ABC-2 type transport system permease protein	5.80E-02
O994_RS00380	EF3209		-1.7	ABC-2 type transport system permease protein	4.90E-02
O994_RS00190	EF3259		-2.0	Gx transporter family protein	1.10E-04
O994_RS14155	EF3327		-2.3	citrate-$Mg2^+$:H+	1.00E-05
**Metabolism of cofactors and vitamins**					
O994_RS08880	EF2056	*ubiA*	1.4	1,4-dihydroxy-2-naphthoate octaprenyltransferase	9.70E-02
O994_RS11425	EF2767		2.4	transcriptional regulator	1.50E-04
O994_RS06970	EF1637	*eutT*	-2.5	ATP:cob(I)alamin adenosyltransferase	1.20E-04
**Metabolism of other amino acids**					
O994_RS05480	EF1338	*trxB*	1.9	thioredoxin-disulfide reductase	5.20E-08
O994_RS03365	EF0930	*metG*	-1.6	methionyl-tRNA synthetase	2.50E-02
O994_RS07920	EF1858	*panD*	-1.6	aspartate α-decarboxylase	1.40E-02

O994_RS07925	EF1859	*panC*	-1.9	pantoate--β-alanine ligase	8.50E-06
O994_RS11290	EF2738		-2.0	thioredoxin reductase/glutathione-like protein	1.10E-02
**Nucleotide Metabolism**					
O994_RS13400	EF0171	*adD*	2.9	adenosine deaminase	7.40E-24
O994_RS13150	EF0228	*adk*	-2.4	adenylate kinase	7.00E-17
O994_RS07335	EF1713	*pyrF*	-1.5	orotidine 5'-phosphate decarboxylase	2.70E-02
O994_RS07360	EF1718	*pyrC*	-2.0	dihydroorotase	5.70E-03
O994_RS07365	EF1719	*pyrB*	-1.9	aspartate carbamoyltransferase catalytic subunit	1.10E-02
O994_RS07375	EF1721	*pyrR*	-2.5	bifunctional pyrimidine regulatory protein	2.10E-05
O994_RS14325	EF3293	*guaB*	-1.7	inosine 5'-monophosphate dehydrogenase	6.10E-04
**Other**					
O994_RS01430	EF0287		2.7	elongation factor P	4.80E-06
O994_RS02635	EF0771	*clpP*	2.5	ATP-dependent Clp endopeptidase proteolytic subunit	1.50E-13
O994_RS03900	EF1038		3.1	lipoprotein	7.40E-12
O994_RS06755	EF1590		2.9	protease synthase and sporulation negative regulatory protein	1.10E-07
O994_RS07135	EF1671		2.2	oxidoreductase, zinc-binding	6.90E-07
O994_RS07205	EF1685	*hlyIII*	1.6	hemolysin III	2.60E-02
O994_RS11160	EF2708		1.7	GlsB/YeaQ/YmgE family stress response membrane protein	2.70E-04
O994_RS13790	EF0071		-2.1	lipoprotein	2.40E-05
O994_RS13280	EF0200	*fusA*	-1.9	elongation factor G	1.90E-04
O994_RS12255	EF0452		-2.5	acyl-CoA synthetase	4.90E-05
O994_RS02150	EF0678		-1.8	acetyltransferase	2.50E-02
O994_RS03660	EF0990	*ftsL*	-1.8	cell division protein	5.50E-03
O994_RS04910	EF1226		-2.7	oxidoreductase	1.50E-05
O994_RS04915	EF1227		-2.4	NADPH-dependent oxidoreductase	3.20E-04
O994_RS06510	EF1538	*scpA*	-1.6	segregation and condensation protein A	1.30E-02
O994_RS06515	EF1539	*scpB*	-1.6	Segregation and condensation protein B	2.80E-03
O994_RS06925	EF1625		-1.9	BMC domain-containing protein	4.90E-02
O994_RS10300	EF2490	*cpsF*	-2.7	casular polysaccharide biosynthesis protein	2.00E-11

O994_RS10360	EF2502		-2.0	cell cycle protein	9.10E-05
O994_RS10610	EF2590		-2.1	serine hydrolase: β-lactamase transpeptidase family	5.20E-05
O994_RS00890	EF3096	*smc*	-2.0	chromosome segregation protein SMC	2.10E-04
**Poorly Characterised**					
O994_RS14010	EF0025		2.0	DUF308 domain-containing protein	1.10E-06
O994_RS14005	EF0026		4.1	hypothetical protein	5.20E-43
O994_RS12785	EF0286		2.8	fibronectin-binding protein	5.50E-10
O994_RS12415	EF0419		2.6	hypothetical protein	8.00E-06
O994_RS12170	EF0468		1.8	LemA family protein	9.10E-03
O994_RS12165	EF0469		2.2	hypothetical protein	5.90E-05
O994_RS01945	EF0638		3.3	DUF1307 domain-containing protein	1.50E-20
O994_RS02125	EF0673		3.6	acyltransferase family protein	9.90E-19
O994_RS02445	EF0730		2.6	thioredoxin family protein	1.70E-05
O994_RS02525	EF0747		2.0	DUF1003 domain-containing protein	8.20E-06
O994_RS02765	EF0797		3.9	DUF4097 family β strand repeat protein	1.50E-28
O994_RS02770	EF0798		3.5	DUF1700 domain-containing protein	1.90E-25
O994_RS02820	EF0819		1.7	DUF960 domain-containing protein	6.20E-02
O994_RS03060	EF0867		1.8	VOC family protein	7.40E-02
O994_RS03370	EF0931		3.0	hypothetical protein	1.50E-16
O994_RS03375	EF0932		5.2	hypothetical protein	1.80E-53
O994_RS03845	EF1027	*mprF*	1.9	bifunctional lysylphosphatidylglycerol flippase	8.40E-05
O994_RS03855	EF1029		2.0	hypothetical protein	1.40E-03
O994_RS03860	EF1030		1.7	endonuclease/exonuclease/phosphatase	3.40E-02
O994_RS04130	EF1064		1.8	VOC family protein	1.40E-03
O994_RS04140	EF1067		5.1	hypothetical protein	4.20E-105
O994_RS04230	EF1088		2.3	DsbA family protein	1.20E-04
O994_RS04270	EF1096		2.0	DUF378 domain-containing protein	8.90E-04
O994_RS04290	EF1099		2.6	LPXTG cell wall anchor domain-containing protein	1.50E-10
O994_RS04305	EF1102		4.3	YxeA family protein	4.00E-39
O994_RS04680	EF1180		1.9	CsbD family protein	9.90E-09
O994_RS05850	EF1412		3.8	hypothetical protein	3.70E-24

O994_RS06255	EF1489		2.3	hypothetical protein	1.40E-05
O994_RS06260	EF1490		2.3	hypothetical protein	6.70E-07
O994_RS06315	EF1501		1.9	hypothetical protein	2.40E-05
O994_RS06480	EF1532		6.4	hypothetical protein	5.30E-126
O994_RS06485	EF1533		6.5	hypothetical protein	4.50E-119
O994_RS06530	EF1542		1.8	hypothetical protein	7.40E-04
O994_RS06825	EF1605		3.2	hypothetical protein	2.60E-11
O994_RS06845	EF1609		1.7	DUF2179 domain-containing protein	6.30E-02
O994_RS07105	EF1665		3.0	conjugal transfer protein	9.10E-13
O994_RS07165	EF1677		1.9	hypothetical protein	4.80E-03
O994_RS07180	EF1680		1.5	YozE family protein	7.40E-02
O994_RS07530	EF1751		3.6	putative membrane protein	6.30E-22
O994_RS07535	EF1752		3.7	hypothetical protein	3.20E-30
O994_RS07540	EF1753	*liaX*	3.7	hypothetical protein	1.60E-26
O994_RS07705	EF1789		1.7	SPFH domain-containing protein	9.20E-04
O994_RS07730	EF1794		1.9	DUF2188 domain-containing protein	5.10E-06
O994_RS08145	EF1903		3.3	hypothetical protein	4.70E-25
O994_RS08275	EF1933		2.5	hypothetical protein	1.20E-05
O994_RS08325	EF1946		1.9	hypothetical protein	5.90E-06
O994_RS08330	EF1947		1.9	hypothetical protein	3.10E-06
O994_RS08490	EF1980		1.9	hypothetical protein	7.00E-04
O994_RS08935	EF2067		2.2	YbhB/YbcL family Raf kinase inhibitor-like protein	5.10E-05
O994_RS09295	EF2202		2.2	tspO protein	5.80E-04
O994_RS09340	EF2211		3.8	yxeA family protein	6.00E-28
O994_RS10005	EF2427		2.6	hypothetical protein	9.30E-22
O994_RS10195	EF2467		3.7	hypothetical protein	9.90E-26
O994_RS10240	EF2477		1.8	SPFH domain-containing protein	1.40E-02
O994_RS10245	EF2478		1.6	hypothetical protein	4.00E-02
O994_RS10250	EF2479		1.9	TPM domain-containing protein	3.70E-05
O994_RS10265	EF2483		1.5	hypothetical protein	4.30E-02
O994_RS10410	EF2547		2.1	hypothetical protein	7.40E-04

O994_RS11075	EF2692		1.6	hypothetical protein	4.70E-02
O994_RS11100	EF2697		3.6	putative 5-bromo-4-chloroindoyl phosphate hydrolase	4.20E-27
O994_RS11315	EF2743		1.7	DUF975 family protein	5.20E-04
O994_RS11320	EF2744		2.1	M42 family metallopeptidase	3.30E-09
O994_RS11445	EF2771		4.4	traX family protein	5.40E-61
O994_RS11850	EF2909		1.7	putative iron-sulfur cluster biosynthesis protein	2.00E-04
O994_RS11945	EF2929		1.4	hypothetical protein	9.30E-02
O994_RS01260	EF3018		3.7	hypothetical protein	1.90E-28
O994_RS01245	EF3021	*gst*	1.9	DinB family glutathione transferase	2.10E-04
O994_RS01095	EF3052		2.1	hypothetical protein	2.70E-06
O994_RS01070	EF3057		2.8	hypothetical protein	6.70E-09
O994_RS00545	EF3174		1.9	NAD(P)-dependent oxidoreductase	5.30E-03
O994_RS00265	EF3239		3.5	zinc ribbon domain-containing protein	4.60E-24
O994_RS00120	EF3273		1.9	hypothetical protein	1.30E-03
O994_RS14220	EF3314		1.8	cell wall surface anchor protein	5.80E-03
O994_RS13050	EF0248		-1.6	hypothetical protein	4.90E-02
O994_RS12870	EF0269		-2.9	PRD domain-containing protein	8.40E-08
O994_RS14960	EF0383		-2.6	acyl-CoA synthetase	5.60E-06
O994_RS02605	EF0764		-2.3	hypothetical protein	7.50E-04
O994_RS04615	EF1166		-1.6	YitT family protein	3.00E-02
O994_RS04650	EF1173		-2.2	WecB/TagA/CpsF family glycosyltransferase	8.10E-06
O994_RS04920	EF1228		-1.8	helix-turn-helix domain-containing protein	4.40E-02
O994_RS05465	EF1327		-2.6	BadF/BadG/BcrA/BcrD ATPase	1.20E-06
O994_RS06440	EF1523		-2.0	hypothetical protein	9.70E-06
O994_RS06910	EF1622		-2.2	hypothetical protein	3.70E-02
O994_RS07245	EF1693		-2.2	KH domain-containing protein	2.40E-05
O994_RS10075	EF2441		-2.7	DUF47 family protein	4.30E-08
O994_RS10290	EF2488	*cpsH*	-2.8	lipoprotein	1.90E-15
O994_RS10305	EF2491		-2.9	glycosyl transferase group 2 family protein	4.30E-19
O994_RS10310	EF2492		-2.6	glycosyltransferase family 2 protein	8.50E-08

O994_RS10515	EF2570		-1.9	aldehyde oxidoreductase	1.40E-02
O994_RS00895	EF3095		-1.7	HAD superfamily hydrolase	7.30E-03
O994_RS00195	EF3258		-1.8	NusG domain II-containing protein	1.60E-02
O994_RS00185	EF3260		-2.1	heptaprenyl diphosphate synthase, component II	4.70E-05
O994_RS00090	EF3280		-2.1	U32 family peptidase	8.70E-05
O994_RS14160	EF3326		-4.7	hypothetical protein	4.50E-13
**Repair and Replication**					
O994_RS02045	EF0659		2.0	phage SPO1 DNA polymerase-like protein	2.60E-03
O994_RS04595	EF1162		2.2	helicase	2.30E-13
O994_RS04535	EF1435	*recU*	1.8	recombination protein U	1.50E-03
O994_RS14095	EF0006	*gyrA*	-1.8	DNA gyrase subunit A	1.90E-03
O994_RS07045	EF1653	*rnhB*	-1.5	ribonuclease HII	9.70E-02
**Signal Transduction**					
O994_RS13470	EF0373		2.3	HAMP domain-containing histidine kinase	8.10E-09
O994_RS03350	EF0927		1.6	sensor histidine kinase	6.00E-03
O994_RS13475	EF1703	*phoP*	2.6	alkaline phosphatase synthesis transcriptional regulatory protein	2.90E-16
O994_RS08850	EF2049		3.8	ABC transporter permease	2.60E-30
O994_RS08855	EF2050		3.7	ABC transporter ATP-binding protein	1.30E-28
O994_RS11325	EF2746	*dltD*	2.9	dltD protein	2.30E-17
O994_RS11330	EF2747	*dltC*	2.9	D-alanine-poly(phosphoribitol) ligase subunit 2	7.10E-21
O994_RS11335	EF2748	*dltB*	3.1	basic membrane protein	4.30E-26
O994_RS11340	EF2749	*dltA*	3.1	D-alanine-poly(phosphoribitol) ligase subunit 1	6.10E-25
O994_RS11860	EF2911	*liaR*	1.9	LuxR family DNA-binding response regulator	2.10E-05
O994_RS11865	EF2912	*liaS*	1.7	sensor histidine kinase	8.40E-03
O994_RS11870	EF2913	*liaF*	2.0	cell wall stress response regulator	8.20E-05
O994_RS06950	EF1632		-1.8	two-component system, sensor histidine kinase	2.40E-03
O994_RS14200	EF3318	*citX*	-2.7	2-(5''-triphosphoribosyl)-3'-dephosphocoenzyme-A synthase	5.90E-07
O994_RS14195	EF3319	*citF*	-2.9	citrate lyase subunit α	8.40E-08
O994_RS14190	EF3320	*citE*	-3.0	citrate lyase subunit β	3.40E-06

O994_RS14185	EF3321	*citD*	-2.8	citrate lyase subunit γ	4.70E-05
O994_RS14180	EF3322	*citC*	-2.6	[citrate(pro-3SO-lyase] ligase	2.40E-06
**Transcription**					
O994_RS00290	EF3237	*rpoC*	-1.8	DNA-directed RNA polymerase subunit β	6.00E-03
O994_RS00285	EF3238	*rpoB*	-1.8	DNA-directed RNA polymerase subunit β	3.70E-04
**Transcriptional Regulator**					
O994_RS12395	EF0422		1.9	IclR family transcriptional regulator	4.40E-04
O994_RS01975	EF0644		2.5	LysR family transcriptional regulator	2.60E-06
O994_RS02390	EF0719		2.3	DeoR family transcriptional regulator	9.70E-06
O994_RS03345	EF0926		1.7	DNA-binding response regulator	2.60E-03
O994_RS03515	EF0962		1.6	AraC family transcriptional regulator	3.60E-02
O994_RS14630	EF0966		1.9	MerR family transcriptional regulator	1.40E-03
O994_RS07265	EF1699		2.2	MerR family transcriptional regulator	1.40E-05
O994_RS10000	EF2426		1.5	GntR family transcriptional regulator	5.20E-02
O994_RS10010	EF2428		2.6	PadR family transcriptional regulator	1.90E-20
O994_RS10200	EF2469		3.5	Cro/CI family transcriptional regulator	1.60E-26
O994_RS10820	EF2638	*rex*	1.7	redox-sensing transcriptional repressor	8.60E-04
O994_RS10895	EF2653		1.7	Cro/CI family transcriptional regulator	7.40E-05
O994_RS01415	EF2995		1.7	PucR family transcriptional regulator	1.20E-02
O994_RS01110	EF3049		2.2	RpiR family phosphosugar-binding transcriptional regulator	8.10E-08
O994_RS00540	EF3175		1.6	Rrf2 family transcriptional regulator	4.70E-02
O994_RS00275	EF3261		2.2	AbrB family transcriptional regulator	2.60E-09
O994_RS00280	EF3262		2.2	PemK family transcriptional regulator	1.50E-08
O994_RS00125	EF3272		1.8	zinc-binding Cro/CI family transcriptional regulator	1.60E-03
O994_RS13855	EF0058	*purR*	-2.1	purine operon repressor	7.30E-08
O994_RS03210	EF0899		-1.8	post-transcriptional regulator	8.90E-03
O994_RS03835	EF1025		-2.0	catabolite repression regulator	1.70E-03
O994_RS03960	EF1050		-1.8	DNA-binding response regulator	1.40E-03
O994_RS06660	EF1569	*psr*	-1.7	putative transcriptional regulator	7.60E-03
O994_RS06955	EF1633		-2.0	response regulator	1.20E-03

O994_RS11965	EF2933		-2.7	redox-sensing transcriptional repressor	2.80E-08
O994_RS12030	EF2966		-3.7	BglG family transcriptional antiterminator	1.00E-17
**Translation**					
O994_RS00390	EF3207		1.5	dihydrouridine synthase	8.80E-02
O994_RS13665	EF0100	*serS-1*	-3.5	seryl-tRNA synthetase	1.20E-12
O994_RS13290	EF0198	*rpsL*	-1.8	30S ribosomal protein S12	4.80E-05
O994_RS13285	EF0199	*rpsG*	-1.8	30S ribosomal protein S7	1.00E-05
O994_RS13260	EF0205	*rpsJ*	-1.5	30S ribosomal protein S10	5.20E-02
O994_RS13255	EF0206	*rplC*	-1.6	50S ribosomal protein L3	2.30E-02
O994_RS13250	EF0207	*rplD*	-1.8	50S ribosomal protein L4	4.30E-04
O994_RS13240	EF0209	*rplB*	-1.9	50S ribosomal protein L2	1.60E-03
O994_RS13235	EF0210	*rpsS*	-2.1	30S ribosomal protein S19	1.30E-04
O994_RS13230	EF0211	*rplV*	-2.0	50S ribosomal protein L22	1.10E-04
O994_RS13225	EF0212	*rpsC*	-1.9	30S ribosomal protein S3	2.50E-04
O994_RS13220	EF0213	*rplP*	-1.9	50S ribosomal protein L16	7.40E-04
O994_RS13215	EF0214	*rpmC*	-2.2	50S ribosomal protein L29	1.60E-06
O994_RS13210	EF0215	*rpsQ*	-2.0	30S ribosomal protein S3	1.70E-05
O994_RS13205	EF0216	*rplN*	-1.9	50S ribosomal protein L14	2.50E-04
O994_RS13200	EF0217	*rplX*	-2.0	50S ribosomal protein L24	1.60E-06
O994_RS13195	EF0218	*rplE*	-2.1	50S ribosomal protein L5	1.30E-08
O994_RS13185	EF0220	*rpsH*	-2.3	30S ribosomal protein S8	2.80E-11
O994_RS13180	EF0221	*rplF*	-2.3	50S ribosomal protein L6	3.00E-09
O994_RS13175	EF0223	*rplR*	-2.1	50S ribosomal protein L18	5.50E-05
O994_RS13170	EF0224	*rpsE*	-2.3	30S ribosomal protein S5	6.10E-11
O994_RS13165	EF0225	*rpmD*	-2.2	50S ribosomal protein L30	1.70E-10
O994_RS13160	EF0226	*rplO*	-2.3	50S ribosomal protein L15	1.60E-14
O994_RS13140	EF0230	*rpmJ*	-1.6	50S ribosomal protein L36	1.40E-03
O994_RS13135	EF0231	*rpsM*	-1.6	30S ribosomal protein S13	1.90E-03
O994_RS13130	EF0232	*rpsK*	-1.8	30S ribosomal protein S11	5.00E-04
O994_RS01920	EF0633	*tryS-1*	-2.9	tyrosyl-tRNA synthetase	3.60E-11

O994_RS02780	EF0801	*leuS*	-1.5	leucyl-tRNA synthetase	6.50E-02
O994_RS03200	EF0896	*tgt*	-1.6	queuine tRNA-ribosyltransferase	5.10E-02
O994_RS03655	EF0989	*mraW*	-1.5	16S rRNA (cytosine1402-N4)-methyltransferase	6.20E-02
O994_RS04365	EF1115	*pheS*	-1.9	phenylalanine-tRNA ligase subunit α	1.30E-06
O994_RS05685	EF1379	*alaS*	-1.6	alanyl-tRNA synthetase	1.70E-03
O994_RS07250	EF1694	*rpsP*	-2.0	30S ribosomal protein S16	1.90E-04
O994_RS08440	EF1970	*aspS*	-1.8	aspartyl-tRNA synthetase	1.80E-03
O994_RS08445	EF1971	*hisS*	-2.1	histidyl-tRNA synthetase	3.70E-05
O994_RS08845	EF2048	*rlmN*	-2.0	23S rRNA (adenine(2503)-C(2))-methyltransferase	1.90E-03
O994_RS09860	EF2395	*frr*	-1.5	ribosome recycling factor	1.60E-02
O994_RS09905	EF2406	*glyS*	-2.3	glycyl-tRNA synthetase subunit β	7.60E-13
O994_RS09910	EF2407	*glyQ*	-2.2	glycyl-tRNA synthetase subunit α	4.60E-06
O994_RS11185	EF2715	*rplL*	-1.7	50S ribosomal protein L7/L12	7.40E-05
O994_RS11190	EF2716	*rplJ*	-1.7	50S ribosomal protein L10	1.40E-03
O994_RS11195	EF2718	*rplA*	-1.8	50S ribosomal protein L1	5.90E-04
O994_RS11200	EF2719	*rplK*	-1.5	50S ribosomal protein L11	2.30E-02
O994_RS11955	EF2931	*valS*	-1.5	valyl-tRNA synthetase	9.90E-02
O994_RS00415	EF3202	*rpsN*	-2.2	30S ribosomal protein S14	2.80E-10
**Unclassified: genetic information processing**					
O994_RS12145	EF0473	*nrdH*	1.6	ribonucleoside-diphosphate reductase 2	2.20E-03
O994_RS02680	EF0781		2.0	cold-shock protein	1.70E-05
O994_RS08550	EF1991	*cspC*	1.7	cold shock protein	9.30E-04
O994_RS11000	EF2677	*mecA*	1.9	adaptor protein	7.00E-08
O994_RS07040	EF1652	*dprA*	-1.8	DNA processing protein	1.60E-02
**Unclassified: metatbolism**					
O994_RS00255	EF0362		1.8	chitin binding protein	5.40E-04
O994_RS02575	EF0758		2.8	cadmium-translocating P-type ATPase	2.90E-14
O994_RS05105	EF1268		1.6	cation transporter E1-E2 family ATPase	1.90E-03
O994_RS05290	EF1304	*mgtA-2*	2.2	magnesium-translocating P-type ATPase	8.00E-04
O994_RS05370	EF1311		1.7	hypothetical protein	2.70E-02

O994_RS05545	EF1352	*mgtA*	2.4	magnesium-translocating P-type ATPase	4.70E-21
O994_RS05790	EF1400		2.4	cadmium-translocating P-type ATPase	4.10E-10
O994_RS07185	EF1681	*msrA*	1.5	peptide methionine sulfoxide reductase	1.90E-03
O994_RS11800	EF2899		2.0	pyridine nucleotide-disulfide family oxidoreductase	2.30E-03
O994_RS00595	EF3164	*msrB*	3.2	methionine sulfoxide reductase B	8.50E-18
O994_RS03195	EF0895		-2.0	glycerol dehydrogenase	8.90E-03
O994_RS03840	EF1026		-2.3	hypothetical protein	8.40E-05
O994_RS04905	EF1225	*apbE*	-2.7	thiamin biosynthesis	9.70E-06
O994_RS05530	EF1348		-2.1	glucan 1	4.20E-04
O994_RS06860	EF1612	*pflA*	-2.5	pyruvate formate-lyase activating enzyme	6.30E-02
O994_RS10315	EF2493		-2.0	teichoic acid biosynthesis protein	7.20E-03
O994_RS10355	EF2501		-2.0	arsenate reductase	3.70E-05
O994_RS10655	EF2601		-1.9	acyl carrier protein	1.90E-04
O994_RS11295	EF2739	*ahpC*	-2.6	alkyl hydroperoxide reductase subunit C	8.10E-06
O994_RS00695	EF3140		-3.4	iron-containing alcohol dehydrogenase	1.30E-12
O994_RS00210	EF3255		-1.7	thiamin biosynthesis lipoprotein	3.90E-02
**Xenobiotics biodegradation and metabolism**					
O994_RS10615	EF2591		-2.1	glyoxalase	9.50E-05
**JH2-2 Specific Genes**					
O994_RS00820			1.9	hypothetical protein	9.70E-04
O994_RS02090			2.1	hypothetical protein	2.40E-08
O994_RS03080			3.5	MazG-like family protein	2.80E-08
O994_RS03815			2.8	hypothetical protein	3.90E-09
O994_RS03850			3.0	hypothetical protein	6.50E-25
O994_RS04075			2.5	hypothetical protein	1.10E-11
O994_RS04850			1.9	hypothetical protein	6.90E-04
O994_RS05030			2.5	ABC transporter permease	3.80E-07
O994_RS06250			1.9	phospholipase	9.20E-07
O994_RS08720			2.7	IS30-like element IS6770 family transposase	5.00E-17
O994_RS11130			1.6	IS6 family transposase	5.00E-02

O994_RS12845		2.5	RusA family crossover junction endodeoxyribonuclease	1.00E-04
O994_RS12900		1.5	NAD(P)/FAD-dependent oxidoreductase	4.40E-02
O994_RS13445		1.7	IS30-like element IS6770 family transposase	4.10E-04
O994_RS13455		2.5	hypothetical protein	1.50E-07
O994_RS13460		2.7	hypothetical protein	3.30E-12
O994_RS13820		1.8	IS30-like element IS6770 family transposase	3.90E-07
O994_RS14445		2.6	hypothetical protein	2.60E-06
O994_RS14560		2.0	ABC transporter permease	3.80E-05
O994_RS14575		1.8	ABC transporter permease	2.50E-02
O994_RS14580		2.7	α/β hydrolase	5.10E-17
O994_RS14655		2.1	IS30-like element IS6770 family transposase	2.10E-09
O994_RS14980		1.5	helix-turn-helix domain-containing protein	7.80E-02
O994_RS15025		4.1	hypothetical protein	4.40E-45
O994_RS00010		-2.2	hypothetical protein	2.60E-04
O994_RS01555		-2.3	tRNA-Met	3.20E-04
O994_RS01705		-2.2	tRNA-Ser	1.30E-03
O994_RS02075		-1.8	DNA topoisomerase IV subunit A	7.60E-04
O994_RS03975		-2.3	hypothetical protein	2.70E-10
O994_RS03985		-2.1	GW domain-containing glycosaminoglycan-binding protein	7.00E-04
O994_RS05795		-2.2	tRNA-Ile	9.20E-04
O994_RS06725		-1.9	23S ribosomal RNA	9.70E-04
O994_RS06870		-1.6	DDE-type integrase/transposase/recombinase	4.50E-02
O994_RS08725	*lacC*	-3.2	tagatose-6-phosphate kinase	3.40E-14
O994_RS08875		-2.1	16S ribosomal RNA	2.60E-03
O994_RS09165		-2.7	hypothetical protein	4.30E-06
O994_RS12890		-3.9	hypothetical protein	3.90E-17
O994_RS12895		-3.5	hypothetical protein	1.30E-12
O994_RS13685		-3.9	hypothetical protein	2.80E-11
O994_RS14525		-1.9	23S ribosomal RNA	4.00E-03
O994_RS14770		-1.4	hypothetical protein	7.60E-02

Table S5. The TXB-induced CroRS regulon in *E. faecalis* wild-type.†

JH2-2 Locus Tag	V583 Gene	Gene Name	F/C (log_2)	Gene Function	P_{adj}
**Amino acid Metabolism**					
O994_RS13945	EF0037	*proA*	2.5	γ-glutamyl phosphate reductase	5.60E-17
O994_RS03895	EF1037	*aspD*	2.3	aspartate aminotransferase	1.30E-13
O994_RS04455	EF1133	*dapD*	4.3	2,3,4,5-tetrahydropyridine-2,6-dicarboxylate *N*-acetyltransferase	2.10E-115
O994_RS04460	EF1134		3.7	M20/M25/M40 family peptidase	6.30E-61
O994_RS05390	EF1314	*alaT*	4.9	aminotransferase	2.10E-115
O994_RS09355	EF2214		4.4	glyoxylase	1.10E-55
O994_RS10350	EF2500		3.9	GcvH family protein	1.40E-38
O994_RS11210	EF2721	*sdhb-2*	3.9	L-serine dehydratase, iron-sulfur-dependent subunit β	2.30E-44
O994_RS11215	EF2722	*sdha-2*	3.1	iron-sulfur-dependent L-serine dehydratase subunit α	1.20E-19
**Autolysis**					
O994_RS13030	EF0252		2.6	*N*-acetylmuramoyl-L-alanine amidase	7.50E-17
**Carbohydrate metabolism**					
O994_RS07000	EF1644	*lacX*	2.5	aldolase 1 epimerase	6.40E-17
O994_RS08160	EF1907		1.8	enoyl-CoA hydratase	6.50E-08
O994_RS10030	EF2432		1.8	metallo-β-lactamase superfamily protein	1.10E-06
O994_RS06325	EF1503	*fbp*	-1.5	fructose-1,6-bisphosphatase	9.60E-02
**Cellular community**					
O994_RS01150	EF3041		2.2	pheromone binding protein	1.30E-08
**Drug resistance: antimicrobial**					
O994_RS02970	EF0849	*alr*	2.6	alanine racemase	1.30E-17
O994_RS06490	EF1534		3.4	cyclophilin type peptidyl-prolyl cis-trans isomerase	2.40E-29
**Folding, sorting and degradation**					
O994_RS07015	EF1647	*hslV*	-2.6	ATP-dependent protease	2.60E-15
**Glycan biosynthesis and metabolism**					
O994_RS02520	EF0746	*pbp(6)*	5.4	penicillin binding protein	1.50E-77
O994_RS03665	EF0991	*pbpC*	2.8	penicillin-binding protein C	2.00E-22
O994_RS04525	EF1148	*pbp1A*	2.6	penicillin-binding protein 1A	2.60E-24

O994_RS05270	EF1300		2.9	putative lipid II flippase	5.70E-10
O994_RS06550	EF1546		1.9	LysM domain-containing protein	1.20E-08
O994_RS07470	EF1740	*pbp1B*	2.2	penicillin-binding protein 1B	2.00E-12
O994_RS09150	EF2170	*epaX*	2.1	glycosyl transferase group 2 family protein	7.30E-07
O994_RS09155	EF2171	*epaW*	2.8	dTDP-4-dehydro-6-deoxy-D-glucose	3.70E-21
O994_RS09245	EF2192	*epaG*	2.2	dTDP-glucose 4,6-dehydratase	5.30E-13
O994_RS09250	EF2193	*epaF*	2.7	dTDP-4-dehydrorhamnose 3,5-epimerase	7.30E-27
O994_RS09255	EF2194	*epaE*	2.6	glucose-1-phosphate thymidylyltransferase	4.00E-22
O994_RS09260	EF2195	*epaD*	1.9	glycosyl transferase group 2 family protein	4.50E-06
O994_RS09265	EF2196	*epaC*	1.9	glycosyl transferase group 2 family protein	7.50E-06
O994_RS09270	EF2197	*epaB*	1.9	glycosyl transferase group 2 family protein	2.40E-05
O994_RS10585	EF2585	*murT*	3.3	mur ligase	1.50E-56
O994_RS10590	EF2586	*gatD*	3.0	glutamine amidotransferase	6.40E-34
O994_RS11265	EF2733	*murB*	2.7	UDP-*N*-acetylenolpyruvoylglucosamine reductase	3.60E-19
O994_RS11580	EF2857	*pbp2B*	4.8	penicillin-binding protein 2B	3.00E-85
O994_RS11595	EF2860	*pbp*	2.5	putative peptidoglycan transpeptidase	1.10E-18
O994_RS11880	EF2915	*mltG*	2.9	mltG-like protein	2.10E-32
**Lipid metabolism**					
O994_RS12800	EF0283	*fabF-1*	1.6	3-oxoacyl-ACP synthase	7.90E-03
O994_RS05090	EF1264		1.5	lipoteichoic acid synthase	2.90E-03
O994_RS07815	EF1813		3.4	lipoteichoic acid synthase	1.30E-15
O994_RS10320	EF2494	*cdsA*	2.9	phosphatidate cytidylyltransferase	1.50E-19
O994_RS11690	EF2880	*fabF-2*	1.5	3-oxoacyl-ACP synthase	1.60E-03
O994_RS11695	EF2881	*fabG*	1.8	3-ketoacyl-ACP reductase	1.40E-08
O994_RS11700	EF2882	*fabD*	1.4	ACP *S*-malonyltransferase	1.30E-02
**Membrane Transport**					
O994_RS13970	EF0032		1.7	putative copper transporter	5.80E-05
O994_RS04770	EF1198		3.2	ABC transporter permease	2.60E-28
O994_RS04775	EF1199		3.2	phosphate ABC transporter: substrate-binding protein	1.10E-27
O994_RS07580	EF1761	*ftsE*	1.5	cell division ATP-binding protein	1.50E-02
O994_RS07820	EF1814		3.6	drug resistance transporter EmrB/QacA family protein	4.60E-13

O994_RS07480	EF2074		4.4	ABC transporter ATP-binding protein	5.30E-17
O994_RS10825	EF2639		1.6	multidrug ABC transporter ATP-binding protein	4.60E-03
O994_RS01540	EF0420		-1.6	drug resistance transporter EmrB/QacA family protein	5.50E-02
O994_RS02705	EF0785		-1.7	drug resistance transporter EmrB/QacA family protein	1.30E-03
O994_RS06380	EF1516		-2.2	PTS system transporter subunit IIABC	3.80E-05
**Metabolism of cofactors and vitamins**					
O994_RS03905	EF1039		2.6	HAD superfamily hydrolase	6.40E-18
O994_RS08535	EF1989	*hemH*	-1.9	ferrochelatase	1.70E-03
**Metabolism of terpenoids and polyketides**					
O994_RS03225	EF0902	*mvaK*	2.9	phosphomevalonate kinase	1.30E-14
O994_RS03230	EF0903	*mvaD*	3.7	diphosphomevalonate decarboxylase	1.10E-22
O994_RS05600	EF1363	*mvaS*	4.4	hydroxymethylglutaryl-CoA synthase	7.80E-96
O994_RS14705	EF1364	*mvaE*	4.6	acetyl-CoA acetyltransferase/HMG-CoA reductase	5.50E-93
O994_RS10325	EF2495	*uppS*	3.3	UDP-diphosphate synthase	3.60E-39
O994_RS00245	EF3245	*uppP*	5.2	cell-envelope associated acid phosphatase	1.60E-102
**Nucleotide metabolism**					
O994_RS06555	EF1547	*cmk*	2.1	cytidylate kinase	3.00E-10
O994_RS11360	EF2754	*nrdD*	1.9	anaerobic ribonucleoside triphosphate reductase	1.10E-04
**Other**					
O994_RS03660	EF0990	*ftsL*	3.7	cell division protein	5.20E-39
O994_RS05645	EF1371		1.4	metallo-β-lactamase	6.40E-02
O994_RS06745	EF1586	*nox*	2.5	NADH oxidase	6.60E-12
O994_RS11270	EF2734		3.8	Gfo/Idh/MocA family oxidoreductase	6.90E-58
**Poorly characterised**					
O994_RS02340	EF0708		3.5	hypothetical protein	2.10E-32
O994_RS02785	EF0802		8.8	DUF3955 domain-containing protein	1.30E-110
O994_RS02820	EF0819		3.2	DUF960 domain-containing protein	1.30E-24
O994_RS04450	EF1132		4.9	CBS-domain containing hypothetical protein	9.90E-76
O994_RS04935	EF1231		6.3	metallophosphoesterase	2.60E-107

O994_RS05020	EF1249	*efba*	1.7	fibronectin/fibrinogen-binding protein	2.30E-03
O994_RS05060	EF1258		4.7	hypothetical protein	1.80E-52
O994_RS06420	EF1518		5.1	hypothetical protein	5.30E-88
O994_RS06580	EF1553		1.9	tetratricopeptide repeat protein	3.20E-06
O994_RS08155	EF1906		1.8	hypothetical protein	2.80E-08
O994_RS09045	EF2149		1.6	hypothetical protein	7.20E-04
O994_RS09360	EF2215		4.3	hypothetical protein	1.10E-52
O994_RS09365	EF2216		2.0	FUSC family protein	8.80E-04
O994_RS09620	EF2373		1.6	hypothetical protein	4.10E-03
O994_RS10035	EF2433		1.9	phosphoglycerate mutase	2.00E-06
O994_RS10205	EF2470		4.1	putative metal-dependent phosphohydrolase	1.00E-54
O994_RS10360	EF2502		4.5	cell cycle protein	1.00E-58
O994_RS11505	EF2784		2.9	DUF3042 domain containing protein	6.60E-16
O994_RS11605	EF2862		5.5	hypothetical protein	1.60E-76
O994_RS11950	EF2930		1.8	hypothetical protein	3.40E-03
O994_RS00655	EF3149		2.1	helix-turn-helix domain-containing protein	3.50E-10
O994_RS00650	EF3150		4.3	M16 family peptidase	2.30E-71
O994_RS00645	EF3151		4.2	insulinase family protein	1.60E-58
O994_RS00520	EF3179		1.6	DUF3298 domain-containing protein	6.20E-02
O994_RS00475	EF3188		1.7	hypothetical protein	6.70E-04
O994_RS14110	EF0003		-2.3	RNA binding protein	4.00E-12
**Replication and repair**					
O994_RS03575	EF0972		4.6	DNA repair exonuclease	5.40E-66
O994_RS05275	EF1301		2.7	rod shape determining protein	1.50E-07
O994_RS06750	EF1587	*mutT*	4.4	DNA mismatch repair protein	6.10E-48
O994_RS07020	EF1648	*xerC*	2.5	integrase/recombinase	1.60E-14
O994_RS14105	EF0004	*recF*	-1.7	DNA replication and repair protein	3.90E-04
**Signal Transduction**					
O994_RS05070	EF1260	*yclR*	2.7	DNA-binding response regulator	4.70E-24

O994_RS05075	EF1261	*yclK*	2.8	sensor histidine kinase	3.40E-23
O994_RS07295	EF1705		2.7	phosphate-binding protein	9.00E-21
O994_RS01065	EF3058		4.4	phosphotyrosine protein phosphatase	2.30E-71
O994_RS08900	EF2060	*cydB*	-1.6	cytochrome D ubiquinol oxidase subunit II	3.60E-04
O994_RS08905	EF2061	*cydA*	-1.8	cytochrome D ubiquinol oxidase subunit I	4.00E-07
O994_RS09380	EF2219	*yesM*	-2.6	sensor histidine kinase	4.10E-13
O994_RS14215	EF3315	*citG*	-1.6	triphosphoribosyl-dephospho-CoA synthase	7.70E-03
**Transcriptional regulators**					
O994_RS12190	EF0465		2.0	polyisoprenyl-teichoic acid--peptidoglycan teichoic acid transferase	7.90E-09
O994_RS02975	EF0850		2.1	PemK family transcriptional regulator	4.00E-06
O994_RS03650	EF0988	*mraZ*	3.8	division/cell wall cluster transcriptional repressor	5.30E-36
O994_RS04835	EF1212		4.5	transcriptional regulator	2.90E-71
O994_RS05280	EF1302		4.9	transcriptional regulator	1.30E-40
O994_RS05285	EF1303		5.6	LysR family transcriptional regulator	1.70E-53
O994_RS06660	EF1569	*psr*	3.5	transcriptional regulator	7.90E-30
O994_RS07005	EF1645	*codY*	2.8	transcriptional repressor	9.10E-27
O994_RS11140	EF2703		5.4	LytR family transcriptional regulator	7.80E-30
O994_RS01385	EF3002	*sylA*	2.0	transcriptional regulator	3.00E-04
O994_RS01060	EF3059		4.7	TetR family transcriptional regulator	4.90E-60
O994_RS06375	EF1515		-2.2	β-glucoside operon transcriptional antiterminator	1.40E-03
**Translation**					
O994_RS12980	EF0263	*tilS*	1.8	tRNA(Ile)-lysidine synthase	4.00E-06
O994_RS03465	EF0950		1.9	tRNA threonylcarbamoyladenosine biosynthesis protein	1.00E-03
O994_RS03655	EF0989	*rsmH*	4.0	16S rRNA (cytosine(1402)-*N*(4))-methyltransferase	2.60E-56
**Unclassified metabolism**					
O994_RS05105	EF1268		2.5	cation transporter E_1-E_2 family ATPase	6.00E-23
O994_RS09085	EF2157	*dacA*	3.5	hypothetical protein	9.70E-41
O994_RS01390	EF3001		1.7	protease synthase and sporulation negative regulatory protein	1.90E-02
O994_RS14275	EF3303		4.0	myosin-cross-reactive antigen	1.70E-54

**JH2-2 Specific**			
O994_RS06245	2.1	GNAT family *N*-acetyltransferase	7.10E-07
O994_RS06415	5.0	hypothetical protein	2.10E-43
O994_RS01545	-1.5	multicopper oxidase domain-containing protein	2.40E-02
O994_RS08540	-2.0	tRNA-Ile	6.20E-03

†Fold-change values are representative of differential gene expression between WT versus Δ*croRS* in the presence of TXB.

Table S6. A comparison of RNAseq and qRT-PCR differential gene expression in the *E. faecalis* WT and Δ*croRS* strains in the presence and absence of TXB.[†]

Gene	WT +/- TXB		Δ*croRS* +/- TXB		Δ*croRS* vs WT -TXB		WT vs Δ*croRS* +TXB	
	RNAseq	qRT-PCR	RNAseq	qRT-PCR	RNAseq	qRT-PCR	RNAseq	qRT-PCR
mvaD	2.7	4.9	<1	-0.4	<1	1.9	3.7	1.7
mvaE	4	5.1	<1	-1.3	<1	0.3	4.6	6.1
**EF1518**	8.3	8.4	<1	2	<1	0.9	5.1	5.5
murT	4.9	5	<1	0.8	<1	0.6	3.3	3.6
**EF2915**	3.1	3.4	<1	-0.2	<1	0.5	2.9	3.1
liaR	3.6	3.9	1.9	0.5	<1	2.5	<1	0.9
liaS	4	5.1	1.7	0.4	<1	2	<1	2.6
liaF	3.9	4.5	2	1.4	<1	2.7	<1	3.7
liaX	5.5	5.4	3.7	3.4	<1	0.9	<1	1.1
**EF0927**	<1	1.2	1.6	1.3	<1	0.03	<1	-0.1
**EF2050**	6.3	6.9	3.7	3.2	2.6	3.5	<1	0.2

[†]Genes differentially expressed below the threshold cutoff of +/- 1-fold $\log_2$ have been designated <1 in the RNAseq data presented.

Table S7. Bacterial strains, plasmids and primers used in this study.

Bacterial strain	Description	Reference/Source
E. faecalis JH2-2	Laboratory strain, plasmid free RifR, FscR	[Jacob et al 1975]
E. faecalis ∆*croRS*	JH2-2 strain harbouring an unmarked deletion of *croRS* (EF3290-89)	[Comenge et al 2003]
E. faecalis JH2-2 + pTCVlac_PliaX	JH2-2 strain harbouring pTCVlac_PliaX	This study
E. faecalis ∆*croRS* + pTCVlac_PliaX	∆*croRS* strain harbouring pTCVlac_PliaX	This study
E. faecalis 4BS + pTCVlac_PliaX	4BS mutant strain harbouring pTCVlac_PliaX	This study
**Plasmid**		
pIMAYZ_EF2057	pIMAYZ + EF2057 knockout construct	
pTCVlac_PliaX	pTCVlac + PliaX_*lacZ* reporter construct	This study
**Primers**		
EF2057_AF	Forward primer, upstream flank to generate the pIMAYZ_EF2057 knockout plasmid<br>CCTCACTAAAGGGAACAAAAGCTGGGTACCCACCACAAACCTATGGCAAA	This study
EF2057_BR	Reverse primer, upstream flank to generate the pIMAYZ_EF2057 knockout plasmid<br>GTTAAAGAACGTTCAACGTCAGGA	This study
EF2057_CD	Forward primer, downstream flank to generate the pIMAYZ_EF2057 knockout plasmid<br>GTTCTTTAACGATATTCAACAATTGCCTGATGGCA	This study
EF2057_DR	Reverse primer, downstream flank to generate the pIMAYZ_EF2057 knockout plasmid<br>CGACTCACTATAGGGCGAATTGGAGCTCCGTCGTTTTAATGAGCTTCCTGA	This study
PliaX_F	Forward primer, *liaX* promoter to generate the pTCVlac_PliaX reporter construct<br>AATTT<u>GAATTC</u>GGATGATCGTACTAATG	This study
PliaX_R	Reverse primer, *liaX* promoter to generate the pTCVlac_PliaX reporter construct<br>AATTT<u>GGATCC</u>CTTTCATGGATATTGC	This study
EF0903_qPCR_L	Left primer, qPCR of EF0903<br>AGG CAG TTT ACA AGC GAT GG	This study
EF0903_qPCR_R	Right primer, qPCR of EF0903<br>AAT TCA ATT CCG GGA CCA GC	This study
EF1364_qPCR_L	Left primer, qPCR of EF1364	This study

	ACGGATGCCTTTAGTGGTCA	
EF1364_qPCR_R	Right primer, qPCR of EF1364 TCTCCACAAGCGTTCCTGAT	This study
EF1518_qPCR_L	Left primer, qPCR of EF1518 GCTGGTCTTGGCTATCTTGG	This study
EF1518_qPCR_R	Right primer, qPCR of EF1518 GACCCCATGTGCGATACTTT	This study
EF2585_qPCR_L	Left primer, qPCR of EF2585 ACGCCAAGAATTTGATGAGG	This study
EF2585_qPCR_R	Right primer, qPCR of EF2585 GGTCGCGGAAAATATTCGTA	This study
EF2915_qPCR_L	Left primer, qPCR of EF2915 GCGGTCTGCTTCTTTGACTC	This study
EF2915_qPCR_R	Right primer, qPCR of EF2915 TGCAACTGCAGAAGGTGTTC	This study
EF2911_qPCR_L	Left primer, qPCR of EF2911 TCGTAGGCGAAGCAGAAAAT	This study
EF2911_qPCR_R	Right primer, qPCR of EF2911 CAATCGCCGGATACACTTTT	This study
EF2912_qPCR_L	Left primer, qPCR of EF2912 AATGGATGGGCAAACAAAAG	This study
EF2912_qPCR_R	Right primer, qPCR of EF2912 CTGATAGGACGCAAGTGCAA	This study
EF2913_qPCR_L	Left primer, qPCR of EF2913 TGCTCGCTCAGCTATATCCA	This study
EF2913_qPCR_R	Right primer, qPCR of EF2913 GGCGTTTTACTTGTGGCCTA	This study
EF1753_qPCR_L	Left primer, qPCR of EF1753 GCAAATGGTACCGTGGTTTT	This study
EF1753_qPCR_R	Right primer, qPCR of EF1753 CTGCTTTCACCCGTTTGTTT	This study

EF0927_qPCR_L	Left primer, qPCR of EF0927 TTTTAACCGACCGAAAATGG	This study
EF0927_qPCR_R	Right primer, qPCR of EF0927 AATGCGTCGTTGATCTTGTG	This study
EF2050_qPCR_L	Left primer, qPCR of EF2050 AAGGATGTGATGGGCTTGTT	This study
EF2050_qPCR_R	Right primer, qPCR of EF2050 TGGTTGCCGTTTTGGATAAT	This study

References

Comenge, Y. *et al.* (2003) The CroRS two-component regulatory system is required for intrinsic β-lactam resistance in *Enterococcus faecalis. J Bacteriol* 185(24), pp. 7184–92. doi: 10.1128/JB.185.24.7184-7192.2003.

Jacob, A. E., Douglas, G. J. and Hobbs, S. J. (1975) Self transferable plasmids determining the hemolysin and bacteriocin of *Streptococcus faecalis* var. zymogenes. *J Bacteriol* 121(3), pp. 863–872.

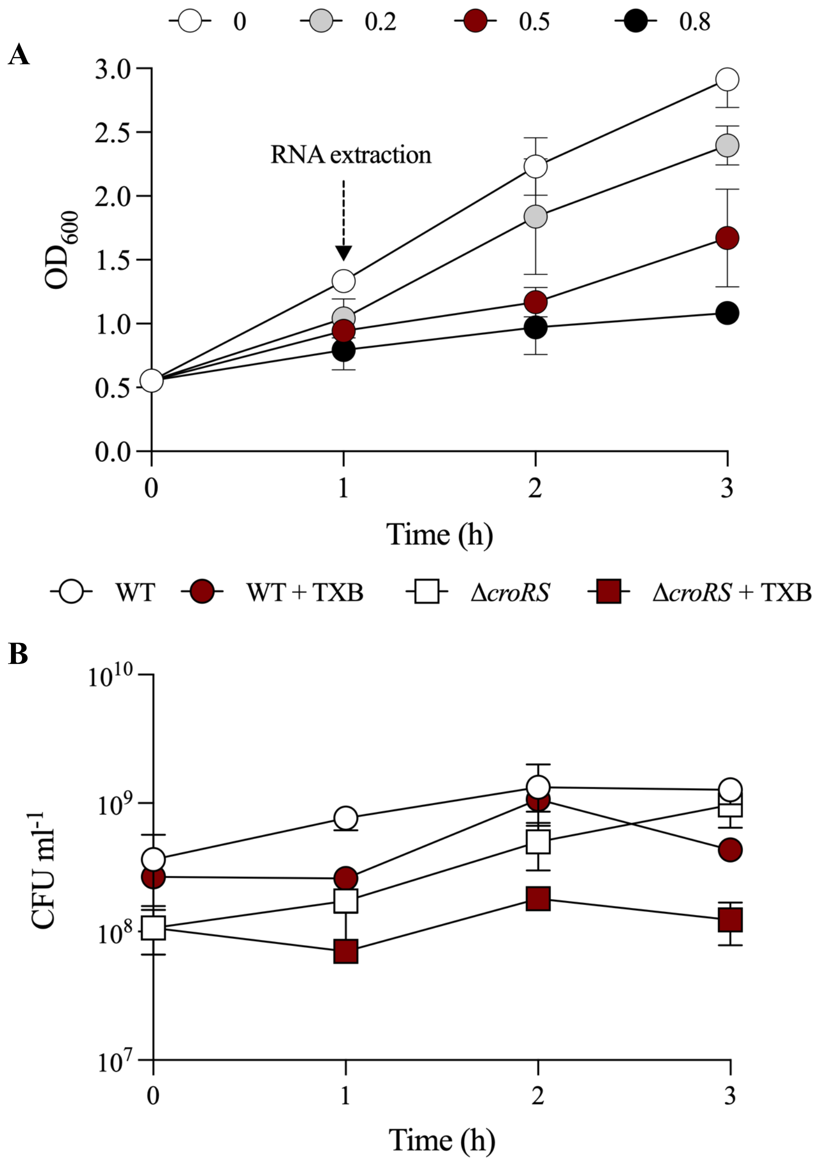

Figure S1. Optimisation of teixobactin concentration for RNA sequencing. *E. faecalis* wild type and Δ*croRS* were grown to mid-exponential phase in BHI broth and challenged with TXB at concentrations of 0, 0.2, 0.5, or 0.8 μg ml^{-1} for 3 h. Growth was measured by OD_{600} at each concentration 1, 2, and 3 h post-challenge for *E. faecalis* Δ*croRS* (**A**) and CFU ml^{-1} for both wild-type and Δ*croRS* at 0.5 μg ml^{-1} (**B**). Following optimisation, RNA was extracted from cultures challenged with and without 0.5 μg ml^{-1} of TXB at 1 h post-challenge. Data is representative of the mean of biological triplicates ± SD.

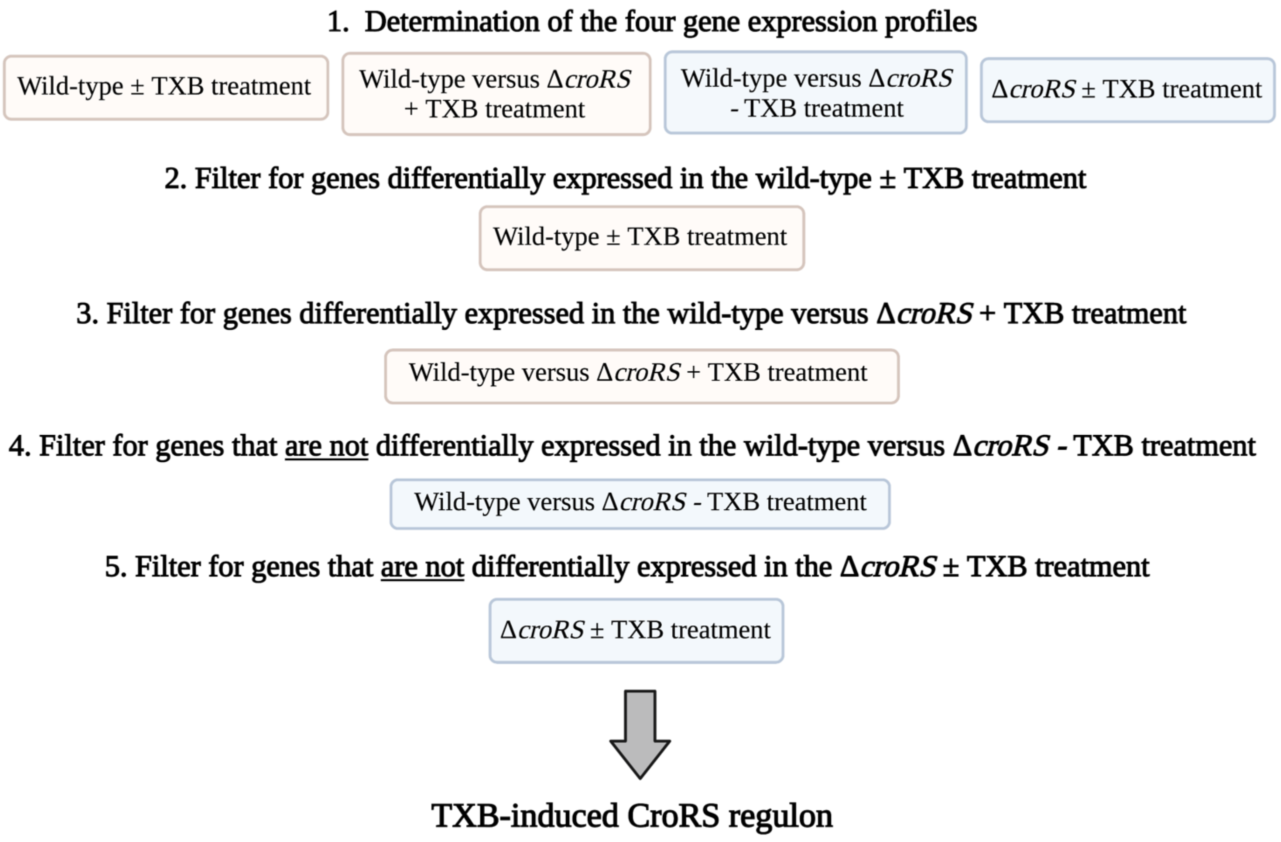

Figure S2. Determing the TXB-induced CroRS regulon. To determine the teixobactin (TXB)-induced CroRS regulon we first determined the four gene expression profiles. Each profile was subsequently filtered using the criteria listed above (Steps 2 – 5) to reach a final set of genes which represented the TXB-induced CroRS regulon.

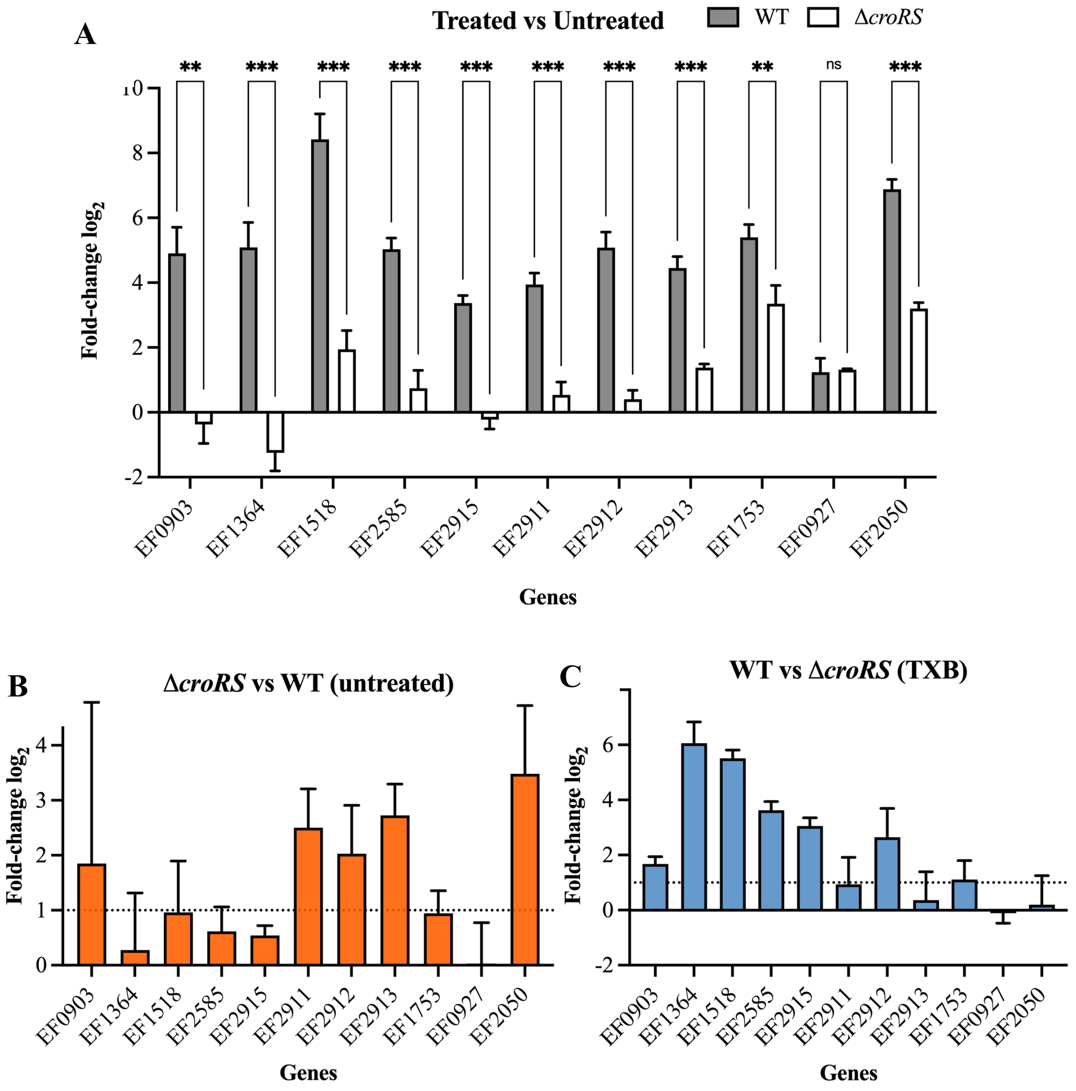

Figure S3. Differential gene expression in the *E. faecalis* WT and Δ*croRS* in the presence and absence of teixobactin. qRT-PCR was carried out for 11 genes using *E. faecalis* WT and Δ*croRS* cDNA to validate changes in gene expression observed during RNA sequencing analyses. Differential gene expression was determined for both *E. faecalis* WT and Δ*croRS* in the presence versus absence of teixobactin (TXB) (**A**), Δ*croRS* versus WT (untreated) (**B**), and WT versus Δ*croRS* (treated) (**C**). Fold-change is represented as the ratio of the mean C_T value normalized to the C_T value for the constitutively expressed EF0013. Results are the mean of biological triplicate ± SD. Statistical significance was determined using multiple unpaired t-tests.

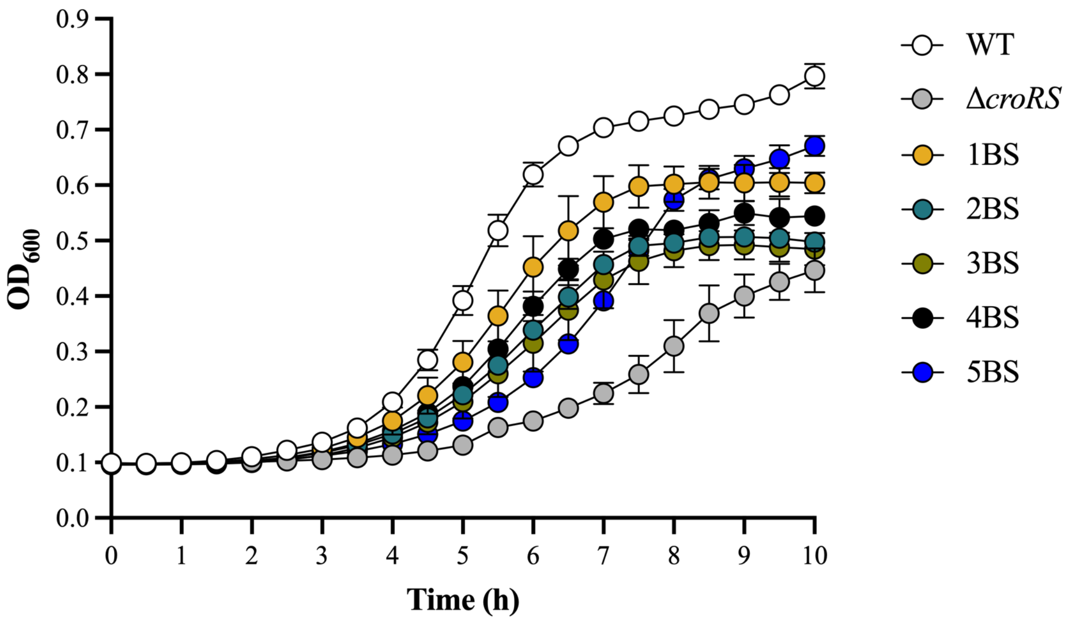

Figure S4. Growth curve of the evolved Δ*croRS* mutants 1BS – 5BS alongside the isogenic Δ*croRS* mutant and WT (JH2-2). Overnight cultures of *E. faecalis* JH2-2, Δ*croRS*, and Δ*croRS*-1BS to 5BS were diluted in fresh BHI and inoculated to an OD_{600} of 0.01 in 96-well plates. Cells were incubated at 37℃ with no aeration and growth was monitored as optical density (OD_{600}) with readings taken every 30 min for 10 h. Results are presented in biological triplicate ± SD.

A

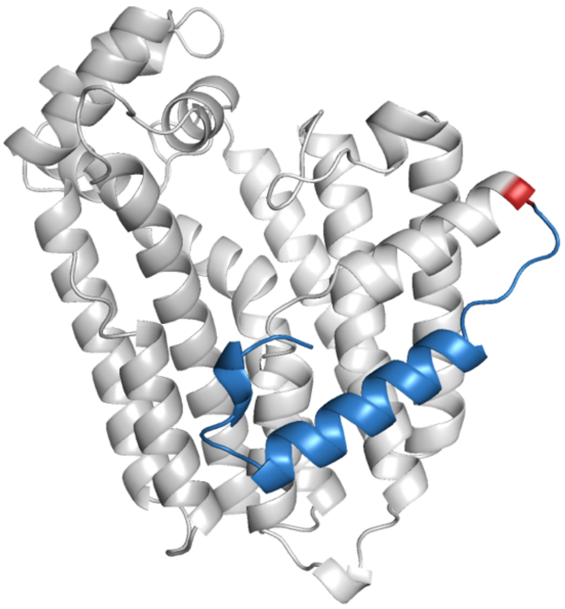

B

```
                                        4BS
  1  MNTFWKSFPDVERSLTETCELIEQTLHTRNEDIEEALVTLARSGGKLLRPAFFFLFAQLGDEEKQE
          1BS
 67  KQQLLKIAASLEILHMATLIHDDIIDDSPLRRGTVTIQSQYGKDVAVYTGDLLFTEFFTLIADAMN
                                                                  2BS
133  GSEFMKINAQGMKRLLLGELDQMSHRFDRRMSIPAYLRSVNGKTAELFSLSCLEGAYFGHSSKEVQ
200  RLAKRIGRHIGIAFQVYDDILDYTADTETLKKPALEDLSQGVYTLPLLFAYQAAPDVFSPYLDKGR
266  AITLEEAAEVAALVNDYHGVTEAQAFAKKVTNKAITDIQQLPDGTAKETLLSLTELLLHRSF
```

Figure S5. A 3D model and amino acid sequence comparison of the putative heptaprenyl diphosphate synthase identifying mutations in the gain of tolerance Δ*croRS* mutants. (A) A 3D model of the truncated 4BS heptaprenyl diphosphate synthase protein. Red indicates the location of the E31* mutation with blue representing the resulting truncated protein expressed in the 4BS mutant. **(B)** Two aspartate-rich motifs (DDXXD) are essential and highly conserved among (E)-prenyl diphosphate synthases (grey). The relative positions of the mutations found within the strains van1, 1BS, 2BS and 4BS are indicated and the amino acid containing the mutation is box. Mutants 2BS and 4BS result in a truncation of the protein at positions Y189 and E31 respectively, due to the introduction of a STOP codon, while in the 1BS mutant, a deletion of 9 bp (216_225) results in a frameshift at K72.

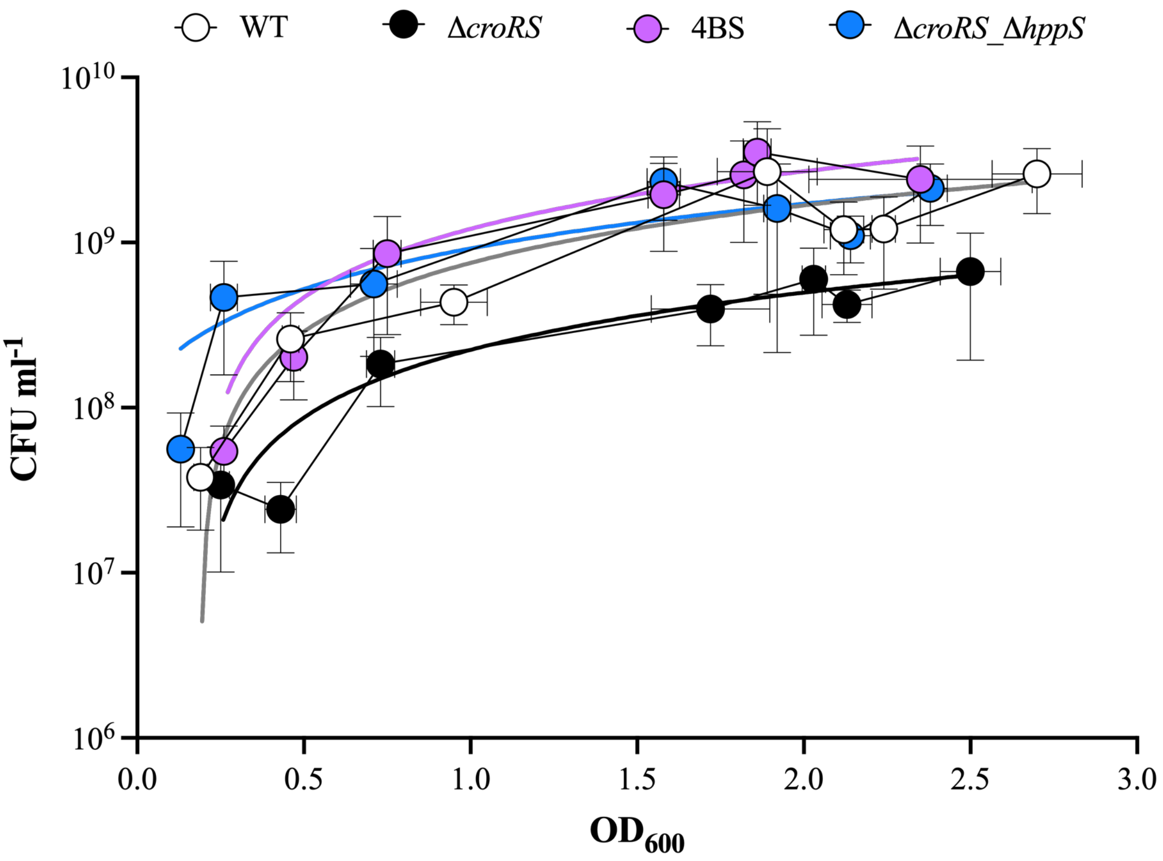

Figure S6. Growth curve of the *E. faecalis* wild-type, Δ*croRS*, 4BS and Δ*croRS*_Δ*hppS* strains. *E. faecalis* strains were normalised to a starting OD_{600} of 0.05 and grown in BHI broth at 37°C with no agitation for 24 h. Growth was measured as both optical density (OD_{600}) and colony forming units per ml (CFU ml^{-1}) every hour for 7 h to reach stationary phase. Results were plotted as OD_{600} against CFU ml^{-1} to observe growth as a ratio over time. Data was analysed for a line of best fit using a simple linear regression model.
